# Addendum: Widespread potential for phototrophy and convergent reduction of lifecycle complexity in the dimorphic order *Caulobacterales*

**DOI:** 10.1038/s41467-026-72348-1

**Published:** 2026-04-24

**Authors:** Joel Hallgren, Jennah E. Dharamshi, Alejandro Rodríguez-Gijón, Julia Nuy, Sarahi L. Garcia, Kristina Jonas

**Affiliations:** 1 Department of Molecular Biosciences, The Wenner-Gren Institute, Science for Life Laboratory, Stockholm University, Stockholm, Sweden; 2 Department of Ecology, Environment and Plant Sciences, Science for Life Laboratory, Stockholm University, Stockholm, Sweden; 3 Institute for Chemistry and Biology of the Marine Environment (ICBM), Carl von Ossietzky Universität Oldenburg, Oldenburg, Germany; 4 Helmholtz Institute for Functional Marine Biodiversity at the University of Oldenburg (HIFMB), Oldenburg, Germany; 5 Present Address: Department of Organismal Biology, Program in Systematic Biology, Uppsala University, Uppsala, Sweden; 6 Present Address: Environmental Metagenomics, Research Center One Health, University of Duisburg-Essen, Essen, Germany

**Keywords:** Phylogenetics, Bacteriology, Microbial ecology, Microbial genetics

Addendum to: *Nature Communications* 10.1038/s41467-025-65642-x, published online 12 December 2025

In a recent article published in *Nature Communications*, we proposed several revisions to the taxonomy of the bacterial order *Caulobacterales*^1^. To meet the criteria for an ‘effective publication’ according to the International Code of Nomenclature of Prokaryotes (ICNP), we here provide protologues for the proposed taxonomic revisions, which were originally included in the electronic supplementary material rather than the main article (see Note 1 to Rule 25a of the ICNP)^2^. Motivations for these proposals are based on our phylogenomic analyses and are found in the ‘Supplementary Note 2’ of Hallgren et al.^1^.

## Descriptions of new taxa

### Description of *Aquidulcibacteraceae* fam. nov

*Aquidulcibacteraceae* (A.qui.dul.ci.bac.ter.a.ce’ae. N.L. masc. n. *Aquidulcibacter*, type genus of the family; -*aceae* ending to denote a family; N.L. fem. pl. n. *Aquildulcibacteraceae* the family of the genus *Aquidulcibacter*).

Gram-negative, rod-shaped bacteria. Do not form spores. Motile by means of a single polar flagellum. Some species form one or multiple prosthecae. Reproduce by binary fission or by prosthecal budding. Some species form rosettes. Colonies are circular, and white, yellow, or pink. Chemotrophic or phototrophic heterotrophs. Strict or facultative aerobes. Grow optimally in absence of NaCl. Q-10 is the major respiratory quinone. Members of the family can be isolated from cyanobacterial aggregates, soil, or water treatment facilities, and occur in algal consortia and freshwater. The G + C content range is 41.4–63.5%. Currently, the family comprises the type genus *Aquidulcibacter* Cai et al.^3^, and the genera *Pseudaquidulcibacter* Liu et al.^4^ and *Vitreimonas* Asem et al.^5^. Belongs to the order *Caulobacterales* and the class *Alphaproteobacteria*.

### Description of *Vitreimonas silvestris* comb. nov

*Vitreimonas silvestris* (sil.ves’tris. L. fem. adj. *silvestris*, belonging to a forest, referring to the forest soil from which the type strain was isolated).

Basonym: *Terricaulis silvestris* Vieira et al.^6^.

The description is as given by Vieira et al.^6^. The type strain is 0127_4^T^ (=DSM 104635^T^ = CECT 9243^T^).

### Description of *Poindextera* gen. nov

*Poindextera* (Poin.dex’te.ra. N.L. fem. n. *Poindextera*, named after Jeanne S. Poindexter, who contributed greatly to the study of the *Caulobacteraceae*).

Gram-negative, rod-shaped bacteria. Do not form spores. Reproduce by binary fission. Colonies are circular, convex, and unpigmented. Aerobic, chemotrophic, mesophilic. Catalase-negative and oxidase-positive. The major respiratory quinone is Q-10. The major fatty acids are C_18:1_ ω7c and C_16:0_. Phosphatidylglycerol, three unidentified glycolipids, and one unidentified phosphoglycolipid are the major polar lipids. Belongs to the family *Caulobacteraceae* in the class *Alphaproteobacteria*. The type species is *Poindextera montana*.

### Description of *Poindextera montana* comb. nov

*Poindextera montana* (mon.ta’na. L. fem. adj. *montana*, of a mountain).

Basonym: *Phenylobacterium montanum* Tang et al.^7^.

The description is as given by Tang et al.^7^. The type strain is S6^T^ (=NBRC 115419^T^ = GCMCC 1.18594^T^).

### Description of *Oceanicaulis satelles* comb. nov

*Alkalicaulis satelles* (sa.tel.’les. L. masc./fem. n. *satelles*, attendant, escort).

Basonym: *Alkalicaulis satelles* Kevbrin et al.^8^.

The description is as given by Kevbrin et al.^8^. The type strain is G-192^T^ (=KCTC 72746^T^ = VKM B-3306^T^).

## Taxonomic opinion matters

### Emended description of the genus *Vitreimonas* Asem et al.^5^

The description is as given by Asem et al.^5^ with the following amendment. Cells are motile by means of a single flagellum and produce one or multiple prosthecae. Some members form branched prosthecae. Cells reproduce by binary fission or by prosthecal budding. Catalase and oxidase reactions, as well as major fatty acid profiles, vary between species. Polar lipids include glycolipids. Phylogenetically belongs to the family *Aquidulcibacteraceae*.

### *Hyphomonas hirschiana* (Weiner et al.^9^) pro synon. *Hyphomonas neptunia* corrig. (Leifson^10^) Moore et al.^11^

*Hyphomonas hirschiana* Weiner et al.^9^ is a later heterotypic synonym of *Hyphomonas neptunia* corrig. (Leifson)^10^ Moore et al.^11^. The description is based on the descriptions of *Hyphomonas neptunia* corrig. (Leifson)^10^ Moore et al.^11^ and *Hyphomonas hirschiana* Weiner et al.^9^. The description of the species *Hyphomonas neptunia* corrig. was given by Moore et al.^11^. Strain VP5^T^ (=ATCC 33886^T^ = CIP 106773^T^ = DSM 5152^T^), the type strain of *Hyphomonas hirschiana*, is a reference strain of *Hyphomonas neptunia*. The type strain is strain 14–15^T^ (=ATCC 15444^T^ = BCRC 10690^T^ = CCRC 10690^T^ = DSM 5154^T^ = IFAM LE-670^T^ = IFO 14232^T^ = LE 670^T^ = NBRC 14232^T^ = NCIMB 2023^T^).

Further, in the version of the article initially published, in the ‘An updated species phylogeny of the *Caulobacterales*’ section, the accession ID for the taxonomic proposals was incomplete and has now been amended to seqco.de/r:9aocwnme in the HTML and PDF versions of the article.
