# Supplementary material for: Widespread potential for phototrophy and convergent reduction of lifecycle complexity in the dimorphic order Caulobacterales
Source: Nat Commun. 2025 Dec 12;16:11003. doi: 10.1038/s41467-025-65642-x (PMC12700997; doi:10.1038/s41467-025-65642-x)
Supplement: Supplementary file 1 — Supplementary Information [file 41467_2025_65642_MOESM1_ESM.pdf]

SUPPLEMENTARY INFORMATION

Widespread potential for phototrophy and convergent reduction of lifecycle complexity within the dimorphic order *Caulobacterales*

Joel Hallgren[1], Jennah E. Dharamshi[2,5], Alejandro Rodríguez-Gijón[2], Julia Nuy[2,6], Sarahi L. Garcia[2,3,4], and Kristina Jonas[1*]

[1] Department of Molecular Biosciences, The Wenner-Gren Institute, Science for Life Laboratory, Stockholm University, Stockholm, Sweden.

[2] Department of Ecology, Environment and Plant Sciences, Science for Life Laboratory, Stockholm University, Stockholm, Sweden.

[3] Institute for Chemistry and Biology of the Marine Environment (ICBM), Carl von Ossietzky University of Oldenburg, Oldenburg, Germany

[4] Helmholtz Institute for Functional Marine Biodiversity at the University of Oldenburg (HIFMB, Oldenburg, Germany

[5] Present address: Department of Organismal Biology, Program in Systematic Biology, Uppsala University, Uppsala, Sweden.

[6] Present address: Environmental Metagenomics, Research Center One Health, University of Duisburg-Essen, Essen, Germany.

*Corresponding author, kristina.jonas@su.se

SUPPLEMENTARY NOTES 1

Supplementary Note 1 | Taxonomic descriptions according to the International Code of Nomenclature of Prokaryotes (ICNP) 1

Description of *Aquidulcibacteraceae* fam. nov. 1

Emended description of the genus *Aquidulcibacter* Cai *et al*. 2017 1

Emended description of the genus *Pseudaquidulcibacter* Liu *et al*. 2022 1

Emended description of the genus *Vitreimonas* Asem *et al*. 2020 1

Description of *Vitreimonas silvestris* comb. nov. 1

Description of *Poindextera* gen. nov. 2

Description of *Poindextera montana* comb. nov. 2

Description of *Oceanicaulis satelles* comb. nov. 2

Hyphomonas neptunia corrig. (Leifson 1964) Moore *et al*. 1984 (syn. *Hyphomonas hirschiana* Weiner *et al*. 1985) 2

Supplementary Note 2 | Additional taxonomic notes 3

Motivation for the description of *Aquidulcibacteraceae* fam. nov. 3

Terricaulis silvestris Vieira *et al*. 2020 belongs to the genus *Vitreimonas* Asem *et al*. 2020 3

Phenylobacterium montanum Tang *et al*. 2025 does not belong to the genus *Phenylobacterium* Lingens *et al*. 1985 3

Alkalicaulis satelles Kevbrin *et al*. 2021 belongs to the genus *Oceanicaulis* Strömpl *et al*. 2003 4

Hyphomonas hirschiana Weiner *et al*. 1985 belongs to the species *Hyphomonas neptunia* corrig. (Leifson 1964) Moore *et al*. 1984 4

Additional taxonomic notes 4

Supplementary Note 3 | Phylogeny, ecology, and physiology of *Acaudatibacter* gen. nov. 6

Phylogeny of *Acaudatibacter* gen. nov. 6

Ecology and putative physiology of *Acaudatibacter* gen. nov. 6

Ecology and physiology of *Ac. aquilonius* sp. nov., *Ac. boreus* sp. nov., and *Ac. lapponiensis* sp. nov. 7

Supplementary Note 4 | Taxonomic descriptions according to the SeqCode code of nomenclature 9

Description of *Acaudatibacter* gen. nov. 9
Description of *Acaudatibacter aquilonius* sp. nov. 9
Description of *Acaudatibacter boreus* sp. nov. 10
Description of *Acaudatibacter lapponiensis* sp. nov. 11

SUPPLEMENTARY FIGURES 13

SUPPLEMENTARY TABLES 53

SUPPLEMENTARY MOVIE LEGENDS 56

SUPPLEMENTARY DATA LEGENDS 57

REFERENCES FOR SUPPLEMENTARY INFORMATION 61

SUPPLEMENTARY NOTES

Supplementary Note 1 | Taxonomic descriptions according to the International Code of Nomenclature of Prokaryotes (ICNP)

Based on our phylogenomic analyses, we make the following taxonomic proposals according to the International Code of Nomenclature of Prokaryotes (ICNP). Motivations for these proposals are found in **Supplementary Note 2**.

Description of *Aquidulcibacteraceae* fam. nov.

Aquidulcibacteraceae (*A.qui.dul.ci.bac ter.a.ce'ae*. N.L. masc. n. *Aquidulcibacter*, type genus of the family; -*aceae* ending to denote a family; N.L. fem. pl. n. *Aquildulcibacteraceae* the family of the genus *Aquidulcibacter*).

Gram-negative, rod-shaped bacteria. Do not form spores. Motile by means of a single polar flagellum. Some species form one or multiple prosthecae. Reproduce by binary fission or by prosthecal budding. Some species form rosettes. Colonies are circular, and white, yellow, or pink. Chemotrophic or phototrophic heterotrophs. Strict or facultative aerobes. Grow optimally in absence of NaCl. Q-10 is the major respiratory quinone. Members of the family can be isolated from cyanobacterial aggregates, soil, or water treatment facilities, and occur in algal consortia and freshwater. The G+C content range is 41.4–63.5%. Currently, the family comprises the type genus *Aquidulcibacter* Cai *et al*. 2017[1], and the genera *Pseudaquidulcibacter* Liu *et al*. 2022[2] and *Vitreimonas* Asem *et al*. 2020[3]. Belongs to the order *Caulobacterales* and the class *Alphaproteobacteria*.

Emended description of the genus *Aquidulcibacter* Cai *et al.* 2017

The description is as given by Cai *et al*. 2017[1] with the following amendment. Phylogenetically belongs to the family *Aquidulcibacteraceae*.

Emended description of the genus *Pseudaquidulcibacter* Liu *et al.* 2022

The description is as given by Liu *et al*. 2022[2] with the following amendment. Phylogenetically belongs to the family *Aquidulcibacteraceae*.

Emended description of the genus *Vitreimonas* Asem *et al.* 2020

The description is as given by Asem *et al*. 2020[3] with the following amendment. Cells are motile by means of a single flagellum and produce one or multiple prosthecae. Some members form branched prosthecae. Cells reproduce by binary fission or by prosthecal budding. Catalase and oxidase reactions, as well as major fatty acid profiles, vary between species. Polar lipids include glycolipids. Phylogenetically belongs to the family *Aquidulcibacteraceae*.

Description of *Vitreimonas silvestris* comb. nov.

Basonym: *Terricaulis silvestris* Vieira *et al*. 2020[4]. *Vitreimonas silvestris* (*sil.ves'tris*. L. fem. adj. *silvestris*, referring to the forest soil from which the type strain was isolated) is closely related to *Vitreimonas flagellata* according to phylogenomics and genomic sequence similarity, but is different from this species by morphology and mode of cell division[3, 4]. Differs from *Vitreimonas flagellata* in the oxidase and catalase tests, by its fatty acid profile, and by its salt tolerance. Further distinguished from *Vitreimonas flagellata* by its lack of activities for the enzymes α-chymotrypsin, esterase (C4),

esterase lipase (C8), α-fucosidase, naphthol-AS-BI phosphohydrolase, and trypsin. The type strain is 0127_4^{T} (= DSM 104635^{T} = CECT 9243^{T}).

Description of *Poindextera* gen. nov.

Poindextera (*Poin.dex'te.ra*. N.L. fem. n. *Poindextera*, named after Jeanne S. Poindexter, who contributed greatly to the study of the *Caulobacteraceae*).

Gram-negative, rod-shaped bacteria. Do not form spores. Reproduce by binary fission. Colonies are circular, convex, and unpigmented. Aerobic, chemotrophic, mesophilic. Catalase-negative and oxidase-positive. The major respiratory quinone is Q-10. The major fatty acids are $C_{18:1}$ ω7c and $C_{16:0}$. Phosphatidylglycerol, three unidentified glycolipids, and one unidentified phosphoglycolipid are the major polar lipids. Belongs to the family *Caulobacteraceae* in the class *Alphaproteobacteria*. The type species is *Poindextera montana*.

Description of *Poindextera montana* comb. nov.

Basonym: *Phenylobacterium montanum* Tang *et al.* 2024[5]. *Poindextera montana* (*mon.ta'na.* L. fem. adj. *montana*, of a mountain) is phylogenetically distinct from members of the genera *Phenylobacterium* and *Caulobacter*. The description is as given by Tang *et al.* 2024[5]. The type strain is S6^{T} (= NBRC 115419^{T} = GCMCC 1.18594^{T}).

Description of *Oceanicaulis satelles* comb. nov.

Basonym: *Alkalicaulis satelles* Kevbrin *et al.* 2021[6]. *Alkalicaulis satelles* is closely related to *Oceanicaulis alexandrii* according to genomic sequence similarity, but is different from this species in the oxidase test, by its fatty acid profile, and by its genetic potential for type II anoxygenic phototrophy. Further distinguished from *Oceanicaulis alexandrii* by its lack of activities for the enzymes lipase (C14), acid phosphatase, and cystine arylamidase, and inability to utilize lactose, lactulose, and maltose. The type strain is G-192^{T} (= KCTC 72746^{T} = VKM B-3306^{T}).

Hyphomonas neptunia corrig. (Leifson 1964) Moore *et al.* 1984 (syn. *Hyphomonas hirschiana* Weiner *et al.* 1985)

The description is based on the descriptions of *Hyphomonas neptunia* corrig. (Leifson 1964)[7] Moore *et al.* 1984[8] and *Hyphomonas hirschiana* Weiner *et al.* 1985[9]. The description of the species *Hyphomonas neptunia* corrig. was given by Moore *et al.* (1984)[8]. Strain VP5^{T} (= ATCC 33886^{T} = CIP 106773^{T} = DSM 5152^{T}), the type strain of *Hyphomonas hirschiana*, is a reference strain of *Hyphomonas neptunia*. The type strain is strain 14-15^{T} (= ATCC 15444^{T} = BCRC 10690^{T} = CCRC 10690^{T} = DSM 5154^{T} = IFAM LE-670^{T} = IFO 14232^{T} = LE 670^{T} = NBRC 14232^{T} = NCIMB 2023^{T}).

Supplementary Note 2 | Additional taxonomic notes

Based on our phylogenomic analyses, we make the following taxonomic notes. Formal taxonomic proposals are found in **Supplementary Note 1**.

Motivation for the description of *Aquidulcibacteraceae* fam. nov.

In phylogenies of concatenated single-copy marker genes conserved among *Alphaproteobacteria*, as well as in phylogenies of concatenated 16S and 23S rRNA genes (16S+23S), type strains of the recently described monotypic genera *Aquidulcibacter*[1], *Pseudaquidulcibacter*[2], and *Terricaulis*[4] assigned to the family *Caulobacteraceae*, as well as *Vitreimonas*[3] assigned to the family *Hyphomonadaceae*, consistently form a cluster separate from other members of *Caulobacteraceae* and *Hyphomonadaceae* (**Fig. 1a**, **Supplementary Figs. S1–S3**). This cluster also includes "*Candidatus* Phycosocius bacilliformis"[10, 11] and "*Candidatus* Viadribacter manganicus"[12] and is placed with good support (98% non-parametric bootstrap proportions [npBP]) sister to *Hyphomonadaceae* in our concatenated single-copy marker gene phylogeny (**Supplementary Fig. S1**), a placement further supported by the 16S+23S phylogeny (**Supplementary Fig. S3**). These results are in line with the current Genome Taxonomy Database (GTDB) taxonomy[13] (R220), which assigns these taxa into the "f__TH1-2" placeholder family separate from *Caulobacteraceae* and *Hyphomonadaceae* (named after *Aquidulcibacter paucihalophilus* TH1-2^{T}, the earliest validly described member of the group). We therefore propose the classification of this cluster as family *Aquidulcibacteraceae* fam. nov., as described in **Supplementary Note 1**. Notably, the placement of *Aquidulcibacteraceae* fam. nov. sister to *Hyphomonadaceae* is perhaps also congruent with the prosthecate budding mode of reproduction of the *Aquidulcibacteraceae* species *Vitreimonas silvestris* comb. nov. (*Terricaulis silvestris*[4]), a trait characteristic of *Hyphomonadaceae* that is not found in *Caulobacteraceae* sensu stricto (**Fig. 1a**).

Terricaulis silvestris Vieira *et al.* 2020 belongs to the genus *Vitreimonas* Asem *et al.* 2020

Terricaulis silvestris 0127_4^{T} [4] and *Vitreimonas flagellata* SYSU XM001^{T} [3] are closely related and should be considered separate species of the same genus (**Supplementary Figs. S1–S2**), given their ANI of 79.6% below the 95% species cutoff[14, 15] (**Supplementary Data 15**) and their high AAI of 74.7% above the 65% genus cutoff[14, 16] (**Supplementary Data 16**). Consistent with this, in GTDB taxonomy releases R202 and R207, they were both classified as genus *Terricaulis*, and in later releases R214 and onward they have been reclassified as genus *Vitreimonas*. Notably, genomic AAI scores also place "*Candidatus* Viadribacter manganicus"[12] within this genus (**Supplementary Data 16**). Given the earlier description of genus *Vitreimonas*[3] than *Terricaulis*[4], we propose the reclassification of *Terricaulis silvestris* as *Vitreimonas silvestris* comb. nov. (type strain 0127_4^{T} = DSM 104635^{T} = CECT 9243^{T}), and the amendment of *Vitreimonas* Asem *et al.* 2020[3], as described in **Supplementary Note 1**.

Phenylobacterium montanum Tang *et al.* 2025 does not belong to the genus *Phenylobacterium* Lingens *et al.* 1985

In our phylogenomic analyses, the recently described species *Phenylobacterium montanum*[5] consistently clusters in the family *Caulobacteraceae*, but outside of its assigned genus *Phenylobacterium* and its sister genus *Caulobacter* (**Supplementary Figs. S1–S3** and **S9**). Consistent with this phylogenetic placement, pairwise AAI comparisons of *Phenylobacterium* and *Caulobacter* genomes to *P. montanum* S6^{T} are very similar between the two genera and are notably around the proposed 65% genus cutoff[14, 16]; for *Phenylobacterium* (n = 41) AAIs range between 64.77–66.63% and

Caulobacter (n = 43) they range between 64.74–66.26% (**Supplementary Data 16**). Additionally, in the GTDB release R207 and onwards, the genome of strain S6^T has been assigned to the uncharacterized genus-level clade "BOG-935", instead of *Phenylobacterium* or *Caulobacter*. Thus, given that strain S6^T is phylogenetically distinct from the genera *Phenylobacterium* and *Caulobacter*, which otherwise comprise its closest described relatives, this strain represents a new genus within the family *Caulobacteraceae*. For this, we propose the name *Poindextera* gen. nov., and the transfer of *Phenylobacterium montanum* Tang *et al.* 2025[5] to this genus as *Poindextera montana* comb. nov. (descriptions in **Supplementary Note 1**).

Additionally, we note that strain S6^T was reported as non-motile under the studied growth conditions[5]. However, strain S6^T has genetic potential for flagellar motility, including extensive suites of flagellar, chemotaxis, and dimorphic development genes (**Fig. 2b**, **Supplementary Data 5b–d**). Additionally, it has genetic potential for type IV pili and polar holdfast adhesin (**Fig. 2b**). Taken together, the genetic potential of strain S6^T suggests that it has a dimorphic developmental program. Moreover, consistent with its gray, pale colonies on modified SSE/HD agar[5], strain S6^T lacks carotenoid biosynthesis genes (**Supplementary Data 8b,c**).

Alkalicaulis satelles Kevbrin *et al.* 2021 belongs to the genus *Oceanicaulis* Strömpl *et al.* 2003

The genome of *Alkalicaulis satelles* G-192^T [6] has an AAI to *Oceanicaulis alexandrii* DSM 11625^T [17] of 69.7% (**Supplementary Data 16**), placing it in the genus *Oceanicaulis* (**Supplementary Figs. S1–S2**). This conclusion is shared with current GTDB taxonomy (R220), and is further supported by its high (68.3%) percentage of conserved proteins compared to *O. alexandrii*[6] (above the proposed 50% genus cutoff[18]). We therefore propose the reclassification of *Alkalicaulis satelles* as *Oceanicaulis satelles* comb. nov. (type strain G-192^T = KCTC 72746^T = VKM B-3306^T), as described in **Supplementary Note 1**. Notably, *Oceanicaulis satelles* comb. nov. has genes for type II anoxygenic phototrophy (**Fig. 5b**, **Supplementary Fig. S15**)[19], genetic potential which it shares with multiple uncharacterized *Oceanicaulis* species (**Fig. 5b**, **Supplementary Fig. S15**).

Hyphomonas hirschiana Weiner *et al.* 1985 belongs to the species *Hyphomonas neptunia* corrig. (Leifson 1964) Moore *et al.* 1984

Hyphomonas hirschiana VP5^T [9] and *Hyphomonas neptunia* 14-15^T [7, 8] should be considered the same species given their ANI of ~100% (**Supplementary Data 1a**). This is likely not the result of genome mislabeling, as EMBOSS Needle alignment[20] of their Sanger-sequenced 16S rRNA genes (GenBank accessions KF863147.1 and KF863145.1, respectively) also reveals 100% 16S rRNA identity (not shown). Given priority of publication, *Hyphomonas hirschiana* is therefore to be considered a later heterotypic synonym of *Hyphomonas neptunia*. Thus, we propose the transfer of *H. hirschiana* strains to *H. neptunia*. An emended description of *Hyphomonas neptunia* is provided in **Supplementary Note 1**.

Additional taxonomic notes

We note that the genera *Litorimonas* and *Algimonas* of the *Maricaulaceae* family are paraphyletic (**Supplementary Figs. S1–S2**), with *L. cladophorae* clustering together with *Al. arctica*, sister to *L. taeanensis.* Given the earlier description of genus *Litorimonas*[21] than *Algimonas*[22], reclassification of *Algimonas arctica* as *Litorimonas arctica* comb. nov. (type strain KCTC 32513^T = MCCC 1K00233^T =

SM1216ᵀ) might be warranted—a conclusion consistent with current GTDB taxonomy. However, the genome of *Al. arctica* KCTC 32513^{T} has only 65.2% and 65.5% AAI to *L. taeanensis* DSM 22008^{T} and *L. cladophorae* KCTC 23968^{T}, respectively. We therefore do not formally propose taxonomic emendation of *Al. arctica*.

We further note that in comparison with *Aquidulcibacter paucihalophilus* TH1-2^{T}, "*Candidatus* Phycosocius bacilliformis" BOTRYCO-2 [10, 11] has an AAI of 81.8% (**Supplementary Data 16**) and "*Candidatus* Phycosocius spiralis" BOTRYCO-1 has an AAI of 76.3%[23], placing them in the genus *Aquidulcibacter*. This conclusion is consistent with current GTDB taxonomy (R220). Thus, all members of the genus *Aquidulcibacter* with sequenced genomes have the genetic potential for type II anoxygenic photoheterotrophy (**Supplementary Data 8**)[23].

Supplementary Note 3 | Phylogeny, ecology, and physiology of *Acaudatibacter* gen. nov.

Here follow discussions on the biology and genomic characteristics of members of *Acaudatibacter* gen. nov. (GTDB taxon "g__Palsa-881") to provide context for their taxonomic description. Formal taxonomic proposals according to the SeqCode code of nomenclature are found in **Supplementary Note 4**.

Phylogeny of *Acaudatibacter* gen. nov.

Concatenated phylogenies of single-copy marker genes conserved among *Alphaproteobacteria* support the inclusion of *Acaudatibacter* gen. nov. within the family *Caulobacteraceae* (**Fig. 1a**, **Supplementary Figs. S1**–**S2** and **S9**). The phylogenies place the genus *Acaudatibacter* gen. nov. as a close relative of the genera *Caulobacter* and *Phenylobacterium*. In phylogenies of the alphaproteobacterial genes included in GToTree[24], *Acaudatibacter* is placed as a sister group of the *Caulobacter–Phenylobacterium* clade (**Supplementary Figs. S2** and **S9**). However, in a more robust tree inferred using 72 previously manually curated alphaproteobacterial genes[25], *Acaudatibacter* is instead placed sister to *Phenylobacterium* with good support (100% non-parametric bootstrap support) (**Supplementary Fig. S1**). Support for the placement of *Acaudatibacter* sister to *Phenylobacterium* is also seen in a phylogeny of concatenated 16S and 23S rRNA genes, inferred for the four current representative *Acaudatibacter* metagenome assembled genomes (MAGs) that include such genes (**Supplementary Fig. S3**). Members of the genus have average amino acid identities (AAIs) to species genome representatives from other *Caulobacteraceae* genera that range between 58.0%–68.0% (**Supplementary Data 16**). Consistent with previously proposed genus delineation cutoff of 65% AAI[14, 16], AAIs between genomes of species belonging to the genus (as assigned by GTDB-Tk v2.1.1; GTDB taxonomy release R207) range between 65.4%–78.3%. AAIs between type genomes of species described here (*Ac. aquilonius*, *Ac. boreus*, and *Ac. lapponiensis*), range between 76.3%–78.3%. Thus, among *Acaudatibacter* gen. nov. species represented in our dataset, these three species are most closely related to each other, given that their genomes only have AAIs between 66.1%–72.5% to other species currently represented in the genus, and between 58.8%–66.1% to species of other *Caulobacteraceae* genera. This conclusion is further supported by phylogenomic analysis (**Supplementary Fig. S9**), their genomic repertoire, and their inferred similar physiology (see below). As a final note on their phylogeny, single-gene phylogenies inferred for PufM (**Supplementary Figs. S17–S19**) and BchY (**Supplementary Figs. S20–S21**) orthologs from *Ac. aquilonius*, *Ac. boreus*, and *Ac. lapponiensis* consistently place them as a distinct group that clusters with sequences from members of the genus-level clade "CAIMFV01", another clade of the *Caulobacter–Phenylobacterium* branch of the *Caulobacteraceae* family.

Ecology and putative physiology of *Acaudatibacter* gen. nov.

Sequences of the aquatic species *Ac. aquilonius*, *Ac. boreus*, *Ac. lapponiensis*, and *Ac.* sp. 23796 have been detected in metagenomes from boreal stratified freshwater bodies of Fennoscandia and Canada (**Fig. 6b**, **Supplementary Fig. S22**). In these habitats, *Ac. aquilonius*, *Ac. boreus*, *Ac.* sp. 23796 have been observed to reach particularly high relative abundances (~0.5%–1.5%) (**Fig. 6c**, **Supplementary Fig. S23b**). *Ac.* sp. SZAS AMP-5, another aquatic species recovered from wastewater (Shenzhen, China) (**Supplementary Fig. 6b**), also mapped to freshwater from the River Narmada in India (**Supplementary Fig. S22b**). Other species of the genus were detected in global terrestrial environments, including permafrost and glacier soil samples from the Arctic (Alaska, USA; Stordalen

Mire and Tarfala, Sweden) and Antarctic (Mackay Glacier, Antarctica), and tropical rhizosphere soil (Minas Gerais, Brazil) (**Supplementary Fig. S7b**).

The genus includes two deep-branching species with genetic potential for flagellar motility, type IV pili (T4P), and holdfast adhesin production, and similar cell development regulation gene suites as *Caulobacter crescentus* (*vibrioides*) CB15 and other dimorphic *Caulobacteraceae* (**Fig. 2b**, **Supplementary Fig. S8**). Since the presence or absence of prosthecae currently cannot be predicted from genome annotations alone, it is unclear whether *Acaudatibacter* gen. nov. includes prosthecate species. However, the largest clade of *Acaudatibacter* gen. nov. species (10/12 species) lack most genes for flagella, chemotaxis, and holdfast production, in contrast to the two deep-branching species. Since these putatively holdfast-lacking, non-flagellated species additionally lack a large number of cell development regulatory genes (**Supplementary Fig. S8**), mirroring the symmetrically reproducing apparently monomorphic species *Phenylobacterium immobile* (**Fig. 3**), these species may have monomorphic lifecycles. These putatively monomorphic *Acaudatibacter* species form a monophyletic group (**Supplementary Fig. S9**). Notably, unlike *P. immobile*, most putatively monomorphic *Acaudatibacter* gen. nov. species do encode T4P (**Supplementary Fig. S8**).

Like other *Caulobacteraceae* members, *Acaudatibacter* gen. nov. genomes contain signature genes for the Entner-Doudoroff Pathway, for respiration using an NADH-quinone oxidoreductase and ubiquinol-cytochrome *c* reductase (cytochrome bc_1), for an F-type ATPase, and for biosynthesis of polyphosphate and polyhydroxybutyrate granules (**Supplementary Fig. S16g**, **Supplementary Data 8i** and **13**). As is also typical of *Caulobacteraceae*, most *Acaudatibacter* gen. nov. species have genes for high-affinity phosphate transporters (*pstABCS*) and phosphonate transporters (*phnDEC*), except rhizosphere species *Ac.* sp. 19682 and *Ac.* sp. 19683 which lack detectable *phnDEC* (**Supplementary Data 11** and **13**).

Ecology and physiology of *Ac. aquilonius* sp. nov., *Ac. boreus* sp. nov., and *Ac. lapponiensis* sp. nov.

MAGs of freshwater species *Ac. aquilonius*, *Ac. boreus*, and *Ac. lapponiensis* have been assembled from slightly acidic (pH ~5.0) freshwater samples, at water temperatures between 9.8–13.8°C, 9.5–16.1°C, and 7.4°C respectively (**Supplementary Fig. S7b**). These three species have been detected at high relative abundance in the upper anoxic layers of stratified freshwater bodies during the summer months, with *Ac. aquilonius* also reaching high relative abundance in oxic layers in some sample series (**Fig. 6c**). Such localization patterns indicate facultative anaerobic lifestyles, which is further supported by their genetic potential for aerobic respiration (**Supplementary Fig. S16g**). As further evidence of their potential adaptation to anoxic low-pH freshwater, genomes of *Ac. aquilonius*, *Ac. boreus*, and *Ac. lapponiensis* encode the ferrous iron transporter FeoB (**Supplementary Data 13**); in low-oxygen, low-pH environments, ferrous (Fe^{2+}) iron is abundant, while ferric (Fe^{3+}) iron is predominant under high-oxygen conditions[26]. At these upper anoxic strata, they likely photosynthesize using their genetic potential for type II anoxygenic photosynthesis (**Supplementary Figs. S16a–f**), as it typical of anoxygenic phototrophs[27]. This genetic potential includes a photosynthetic reaction center (RC), a light-harvesting I complex (LH1), and light-harvesting II complex (LH2), bacteriochlorophyll and carotenoid pigments, and a partial (*Ac. boreus*; potentially due to genome incompleteness, see **Results**) or complete (*Ac. aquilonius* and *Ac. lapponiensis*) Calvin-Benson-Bassham (CBB) cycle for carbon fixation (**Supplementary Data 9b**), which includes the accessory genes for red-type RuBisCO activase (*cbbX*) and XuBP phosphatase (*cbbY*) (**Fig. 5c**). Notably, like other *Caulobacterales* phototrophs

(**Supplementary Data 8**), *Ac. aquilonius*, *Ac. boreus*, and *Ac. lapponiensis* lack the PufC photosynthetic reaction center cytochrome *c* subunit and the PufX reaction center protein present in some other *Alphaproteobacteria* (**Supplementary Figs. S15** and **S16e**).

Additionally, the non-homogenous distribution of *Ac. aquilonius*, *Ac. boreus*, and *Ac. lapponiensis* in the water column, suggests that they are able to regulate their buoyancy, despite lacking flagella. Whereas we could identify gas vesicle proteins (Pfam domains: 3×PF00741, 1×PF05120, 1×PF05121, 1×PF05800, 3×PF06386) in the phototroph *Rhodobacter sphaeroides* 2.4.1^{T} (order *Rhodobacterales*), *Caulobacterales* genomes lacked such genes, indicating that these *Acaudatibacter* species possess other buoyancy regulation mechanisms, which could include T4P-mediated cell aggregation.

Based on KEGG Decoder, *Ac. aquilonius*, *Ac. boreus*, and *Ac. lapponiensis* have complete biosynthesis pathways for all standard amino acids, except valine and isoleucine. However, KEGG Decoder predicts these two pathways as incomplete for all *Caulobacterales* genomes, including the known amino acid prototrophs *Asticcacaulis excentricus* AC48^{T} (CB 48^{T})[28], *C. crescentus* CB15 [28], and *C. segnis* TK0059^{T} [29] (**Supplementary Data 13**). The incorrect prediction stems from KEGG Decoder v1.3 requiring the presence of acetolactate synthase II small subunit IlvM [EC:2.2.1.6] (K11258) for these two pathways to be deemed complete (together with K00826, K01687, K00053, K01652, and K01653). However, this KEGG ortholog (KO) is absent from the vast majority of *Caulobacterales* genomes (**Supplementary Data 11** and **12**). Instead, both large and small subunits of the corresponding enzyme [EC:2.2.1.6] are represented in most *Caulobacterales* genomes as K01652 and K01653. Therefore, since *Ac. aquilonius*, *Ac. boreus*, and *Ac. lapponiensis*—like the aforementioned known amino acids prototrophs—merely lack K11258, but otherwise have predicted complete amino acid pathways, we conclude that these *Acaudatibacter* gen. nov. species are likely amino acid prototrophs.

The genetic potential of *Ac. aquilonius*, *Ac. boreus*, and *Ac. lapponiensis* genomes differs on some points, as follows. *Ac. boreus* and *Ac. lapponiensis* have genetic potential for starch/glycogen biosynthesis and degradation, which *A. aquilonius* lacks (**Supplementary Data 13**). *Ac. boreus* and *Ac. lapponiensis* have genes for cytochrome *bd* terminal oxidase, which *A. aquilonius* lacks (**Supplementary Fig. S16g**). *Ac. aquilonius* and *Ac. boreus* encode thiosulfate dehydrogenase TsdA for thiosulfate oxidation, which *Ac. lapponiensis* lacks (**Supplementary Data 13**). *Ac. boreus* has genetic potential for sulfide oxidation using the Sqr sulfide:quinone oxidoreductase (K17218), which *Ac. lapponiensis* and *Ac. aquilonius* lack (except for the low-quality *Ac. aquilonius* MAG "AM-lipid-02-D3_megahit_metabat_bin-0449"). Lastly, *Ac. boreus* and *Ac. lapponiensis* encode the SsuABC (K15553–K15555) sulfonate transporter, which *Ac. aquilonius* lacks.

Supplementary Note 4 | Taxonomic descriptions according to the SeqCode code of nomenclature

The SeqCode register list for *Acaudatibacter* gen. nov., *Acaudatibacter aquilonius* sp. nov., *Acaudatibacter boreus* sp. nov., and *Acaudatibacter lapponiensis* sp. nov. is available under the accession ID 9aocwnme (https://seqco.de/r:9aocwnme).

Description of *Acaudatibacter* gen. nov.

Acaudatibacter (A.cau.da.ti.bac'ter. Gr. pref. *a*-, not, without [inseparable prefix]; L. masc. adj. *caudatus*, tailed or having a tail; N.L. masc. n. *bacter*, rod; N.L. masc. n. *Acaudatibacter*, tailless rod).

Members of this genus have been identified in freshwater, wastewater, and in soils from permafrost active layers, of glacier regions, and of rhizospheres of *Barbacenia macrantha* and *Vellozia epidendroides*. ANI values among genomes representing separate species within the genus range between < 76.6% and 81.0%. AAI values among genomes representing separate species within the genus range between 65.4% and 78.8%. Genomes of this genus notably contain genes for the Entner-Doudoroff Pathway, for aerobic respiration using an NADH-quinone oxidoreductase, a ubiquinol-cytochrome *c* reductase, and a cytochrome *c* oxidase, an F-type ATPase, and for biosynthesis of polyphosphate and polyhydroxybutyrate. Some members have the genetic potential to produce bacteriochlorophyll and/or carotenoid pigments. Most members encode genes for type IV tight-adhesion pili, with some members further encoding genes for flagellar motility, chemotaxis, and holdfast formation, with genetic potential similar to members of the *Caulobacteraceae* family with obligate dimorphic lifecycles. However, most members likely have a monomorphic cell developmental program, as inferred from their lack genes for flagella, chemotaxis, and polar holdfast adhesin, as well as absence of a large number (> 20) of cell cycle regulation and polar morphogenesis genes present in related dimorphic taxa. The genus includes genomes with chemoheterotrophic, photoheterotrophic, and photoautotrophic genetic potentials. The taxon is supported as a genus-level group by phylogenomics and AAI, and corresponds to the GTDB taxonomy (R220) genus "g__Palsa-881".

The nomenclatural type for the genus is *Acaudatibacter aquilonius* GCA_903872075.1^{Ts}.

SeqCode URL: https://seqco.de/i:49710

Description of *Acaudatibacter aquilonius* sp. nov.

Acaudatibacter aquilonius (a.qui.lo'ni.us. L. masc. adj. *aquilonius*, northern, northerly, referring to the recovery of genomes of the organism from northern freshwater bodies).

Twelve metagenome-assembled genomes representing this species were assembled from sequence data obtained from samples taken from lakes Björntjärnen (Sweden), Alinen Mustajärvi (Finland), Keskinen Rajajärvi (Finland), and Valkea Kotinen (Finland). Completeness estimates for genomes, as determined by CheckM (v1.1.3; 'lineage_wf'), are 69.50–97.53%, with 1.62–4.21% estimated contamination. Genome assemblies range between 3.06–4.56 Mbp in size, comprising 100–973 contigs, with a G+C content of 67.37%–67.58%. Estimated complete genome sizes from CheckM range between 4.40–4.68 Mbp. ANI and AAI values between these genomes are 97.8%–100% and 99.3–100%, respectively, while such pairwise comparisons to closely related taxa are below 81.0% and 78.8%, respectively. Phylogenomic analysis of 72 concatenated conserved alphaproteobacterial single-copy genes places

this species in the genus *Acaudatibacter*, in the family *Caulobacteraceae*. The species corresponds to GTDB taxonomy (R220) species "Palsa-881 sp903872075".

Genomes lack multiple genes for flagellar motility, chemotaxis, holdfast adhesin production, and for the caulobacterial obligate dimorphic cell developmental program. Genomes contain genes for type IV tight-adhesion pili; for carotenoid pigment production; for complete biosynthesis pathways of all standard amino acids; for aerobic respiration using cytochrome *c* oxidases cytochrome aa_3 (*coxABC*) and cytochrome cbb_3 (*ccoNOPQ*); for thiosulfate oxidation using thiosulfate dehydrogenase TsdA; for biosynthesis and degradation of polyphosphate and polyhydroxybutyrate; and for high-affinity PstABCS phosphate and PhnDEC phosphonate transporters. In addition, they have genetic potential for photoautotrophy, containing genes for bacteriochlorophyll synthesis, type II anoxygenic photosynthesis using a light-harvesting II (LH2) complex and a reaction center–light-harvesting I supercomplex (RC–LH1), as well as carbon fixation using the Calvin-Benson-Bassham (CBB) cycle, including the accessory genes for red-type RuBisCO activase (*cbbX*) and XuBP phosphatase (*cbbY*). The species has been detected in both oxic and anoxic strata of stratified freshwater bodies in Finland and Sweden. Likely a psychrophilic/mesophilic facultative anaerobe, based on its genetic repertoire and environmental distribution.

The proposed nomenclatural type for the species is the genome Umea_bin-04329^{Ts}, available under the NCBI WGS assembly accession number GCA_903872075.1^{Ts} (BioProject ID PRJEB38681), recovered as a metagenome coassembly from samples taken in autumn of 2018 from the stratified lakes Björntjärnen (lat. 64.12, long. 18.78) and Nästjärnen (lat. 64.15, long. 18.80), both in Umeå, Sweden (metagenomes ERS4600559–565 and ERS4600568–570). It comprises 100 contigs with a total of 4,361,582 bp, and has an estimated completeness of 95.32% and contamination of 1.62%.

SeqCode URL: https://seqco.de/i:49709

Description of *Acaudatibacter boreus* sp. nov.

Acaudatibacter boreus (bo're.us. L. masc. adj. *boreus*, northern, referring to the recovery of genomes of the organism from northern freshwater bodies).

Four metagenome-assembled genomes representing this species were assembled from sequence data obtained from samples taken from anoxic strata of a lake in Kiruna (Sweden), referred to as Ki1. Completeness estimates for genomes, as determined by CheckM (v1.1.3; 'lineage_wf'), are 93.45%–94.46%, with 2.03–3.83% estimated contamination. Genome assemblies range between 4.23 Mbp–4.36 Mbp, comprising 576–699 contigs, with a G+C content of 67.46%–67.60%. Estimated complete genome sizes from CheckM range between 4.52–4.62 Mbp. ANI and AAI values between these genomes are 99.2%–100% and 99.6–100%, respectively, while such pairwise comparisons to closely related taxa are below 80.9% and 77.9%, respectively. Phylogenomic analysis of 72 conserved alphaproteobacterial single-copy genes places this species in the genus *Acaudatibacter*, in the family *Caulobacteraceae*. The species corresponds to GTDB taxonomy (R220) species "Palsa-881 sp903870555".

Genomes lack multiple genes for flagellar motility, chemotaxis, holdfast adhesin production, and for the caulobacterial obligate dimorphic cell developmental program. Genomes contain genes for type IV tight-adhesion pili; for carotenoid pigment production; for complete biosynthesis pathways of all standard amino acids; for aerobic respiration using cytochrome *c* oxidases cytochrome aa_3 (*coxABC*)

and cytochrome *cbb*$_3$ (*ccoNOPQ*), and ubiquinol oxidase cytochrome *bd* (*cydABX*); for thiosulfate oxidation using the thiosulfate dehydrogenase TsdA; for sulfide oxidation using the Sqr sulfide:quinone oxidoreductase; for biosynthesis and degradation of polyphosphate, polyhydroxybutyrate, and starch/glycogen; and for the high-affinity PstABCS phosphate, PhnDEC phosphonate, and SsuABC sulfonate transporters. In addition, they have partial genetic potential for photoautotrophy, containing genes for type II anoxygenic photosynthesis using a light-harvesting II (LH2) complex and a reaction center–light-harvesting I supercomplex (RC–LH1), as well as partial genetic potential for carbon fixation using the Calvin-Benson-Bassham (CBB) cycle. The species has been detected in both oxic and anoxic strata of stratified freshwater bodies in Canada, Finland, and Sweden. Likely a psychrophilic/mesophilic facultative anaerobe, based on its genetic repertoire and environmental distribution.

The proposed nomenclatural type for the species is the genome Ki1-2-2m_bin-386^{Ts}, available under the NCBI WGS assembly accession number GCA_903870555.1^{Ts} (BioProject ID PRJEB38681), recovered 24 July 2018 from a stratified lake in Kiruna, Sweden (lat. 67.93, long. 20.36; from the metagenome ERS4600419). It comprises 692 contigs with a total of 4,349,168 bp, and has an estimated completeness of 94.45% and contamination of 2.03%.

SeqCode URL: https://seqco.de/i:49711

Description of *Acaudatibacter lapponiensis* sp. nov.

Acaudatibacter lapponiensis (lap.po.ni.en'sis. N.L. masc. adj. *lapponiensis*, pertaining to Lapponia, the Latin name for Lapland, the geographical region from which genomes of the organism were recovered).

Four metagenome-assembled genomes representing this species were assembled from sequence data obtained from samples taken from a lake in Kiruna (Sweden), referred to as Ki2, Swedish lake code: 754378-169136. Completeness estimates for genomes, as determined by CheckM (v1.1.3; 'lineage_wf'), are 94.86%–96.97%, with 1.94%–4.47% estimated contamination. Genomes assemblies range between 4.57–4.64 Mbp, comprising 279–357 contigs, with a G+C content of 68.83%–68.84%. Estimated complete genome sizes from CheckM range between 4.73–4.81 bp. ANI and AAI values between these genomes are 99.9%–100% and 99.9–100%, respectively, while such pairwise comparisons to closely related taxa are below 81.0% and 78.8%, respectively. Phylogenomic analysis of 72 conserved alphaproteobacterial single-copy genes places this species in the genus *Acaudatibacter*, in the family *Caulobacteraceae*. The species corresponds to GTDB taxonomy (R220) species "Palsa-881 sp903923135".

Genomes lack multiple genes for flagellar motility, chemotaxis, holdfast adhesin production, and for the caulobacterial obligate dimorphic cell developmental program. Genomes contain genes for type IV tight-adhesion pili; for carotenoid pigment production; for complete biosynthesis pathways of all standard amino acids; for aerobic respiration using cytochrome *c* oxidases cytochrome *aa*$_3$ (*coxABC*) and cytochrome *cbb*$_3$ (*ccoNOPQ*), and for ubiquinol oxidase cytochrome *bd* (*cydA*; partial genetic potential); for biosynthesis and degradation of polyphosphate, polyhydroxybutyrate, and starch/glycogen; for the high-affinity PstABCS phosphate, PhnDEC phosphonate, and SsuABC sulfonate transporters. In addition, they have genetic potential for photoautotrophy, containing genes for type II anoxygenic photosynthesis using a light-harvesting II (LH2) complex and a reaction center–light-harvesting I supercomplex (RC–LH1), as well as carbon fixation using the Calvin-Benson-

Bassham (CBB) cycle, including the accessory genes for red-type RuBisCO activase (*cbbX*) and XuBP phosphatase (*cbbY*). The species has been detected in both oxic and anoxic strata of stratified freshwater bodies in Finland and Sweden. Likely a psychrophilic/mesophilic facultative anaerobe, based on its genetic repertoire and environmental distribution.

The proposed nomenclatural type for the species is the genome Kiruna2_bin-0871^{Ts}, available under the NCBI WGS assembly accession number GCA_903923135.1^{Ts} (BioProject ID PRJEB38681), recovered as a coassembly from samples taken 27 July 2018 from a stratified lake in Kiruna, Sweden (lat. 67.92, long. 20.37; from the metagenomes ERS4600421–424). It comprises 279 contigs with a total of 4,636,946 bp, and has an estimated completeness of 96.47% and contamination of 2.20%.

SeqCode URL: https://seqco.de/i:49712

SUPPLEMENTARY FIGURES

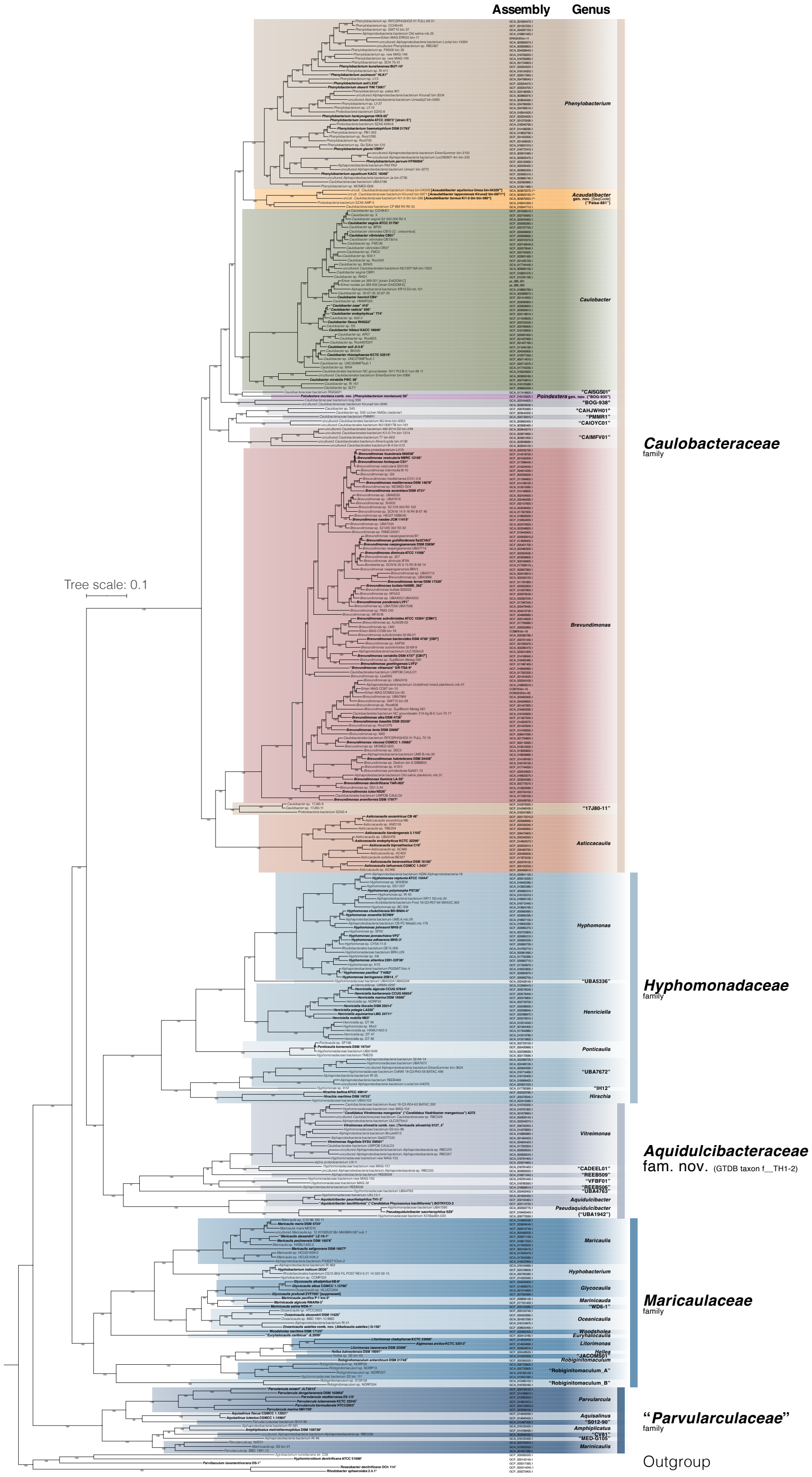

Figure S1 | Legend on next page.

Figure S1 | *Caulobacterales* species phylogeny based on 72 concatenated and manually curated marker genes. Maximum-likelihood (ML) species phylogeny of 72 concatenated and manually curated single-copy marker genes[25] for all 347 *Caulobacterales* species genome representatives, the model organism *C. crescentus* (*C. vibrioides*) CB15, and five outgroup *Alphaproteobacteria* for rooting. Inferred using IQ-TREE[30] with the LG+C60+F+R model of evolution and 100 non-parametric bootstraps (alignment length of 25448 amino acids). The scale bar indicates number of substitutions per site in the phylogeny. Family and genus assignment follows GTDB taxonomy (R207)[31], with minor modifications according to the taxonomic proposals outlined in **Supplementary Notes 1–2**. Same tree as shown in **Fig. 1a**, but with all species labeled.

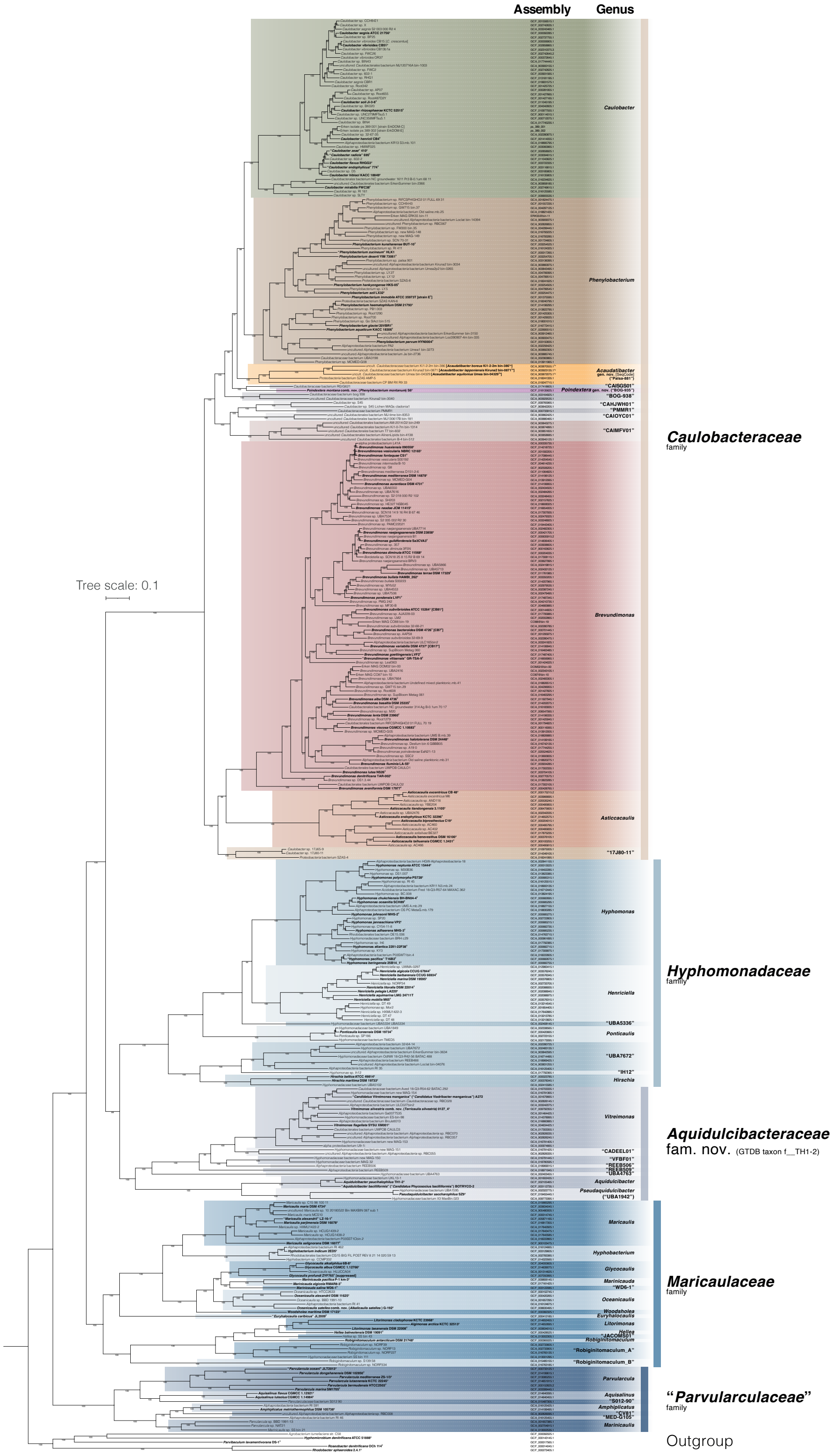

Figure S2 | Legend on next page.

Figure S2 | *Caulobacterales* species phylogeny based on 117 concatenated alphaproteobacterial marker genes. ML species phylogeny of 117 alphaproteobacterial concatenated single-copy marker genes extracted using GToTree[24], for all 347 *Caulobacterales* species genome representatives, the model organism *C. crescentus* (*C. vibrioides*) CB15, and five outgroup *Alphaproteobacteria* for rooting. Inferred using IQ-TREE[30] with the LG+C60+F+G model of evolution and 1000 ultrafast bootstraps (alignment length of 21766 amino acids). The scale bar indicates number of substitutions per site in the phylogeny. Family and genus assignment follows GTDB taxonomy (R207)[31], with minor modifications according to the taxonomic proposals outlined in **Supplementary Notes 1–2**.

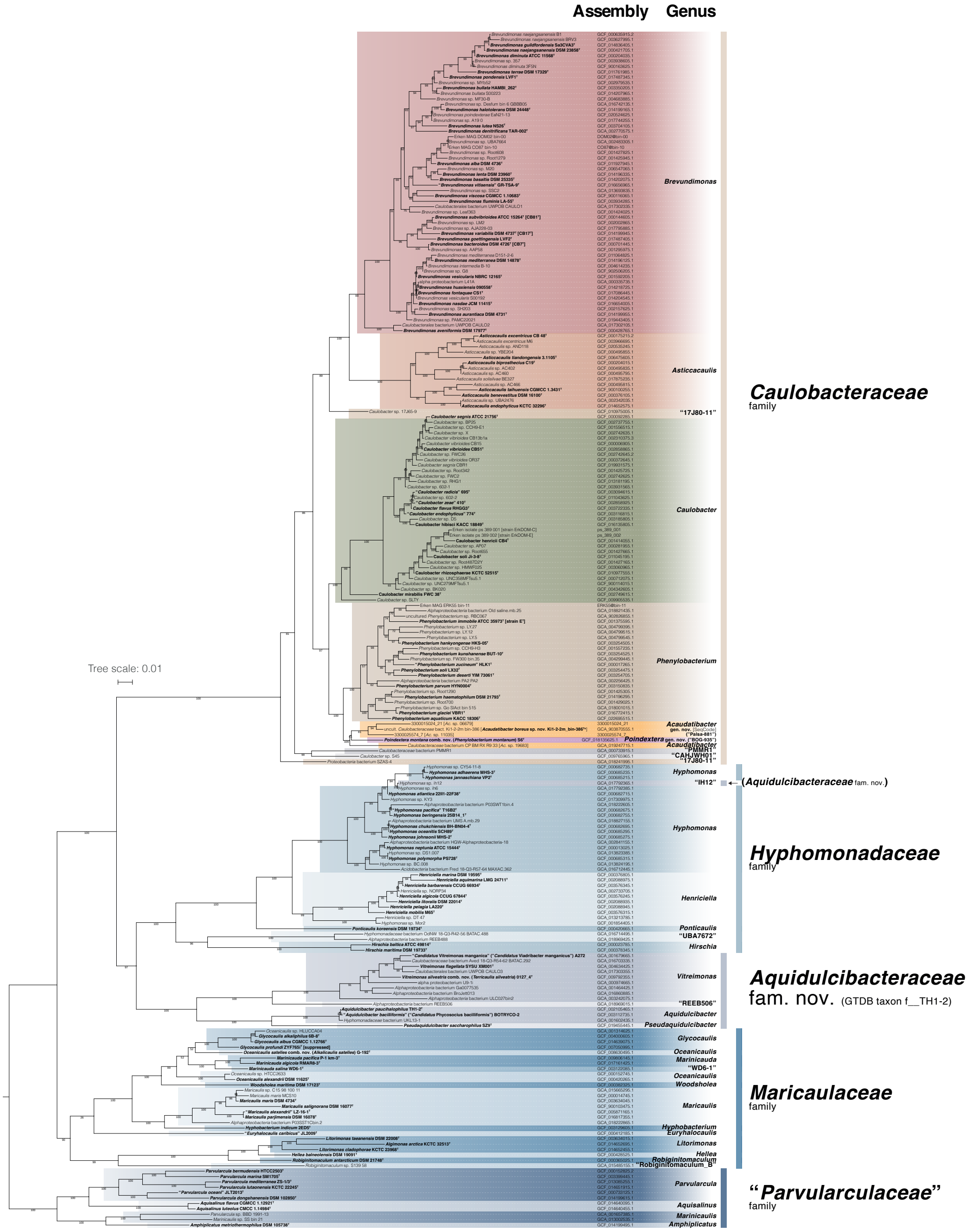

Figure S3 | *Caulobacterales* species phylogeny based on concatenated 16S and 23S rRNA genes. ML species phylogeny of concatenated 16S and 23S rRNA genes, for the model organism *C. crescentus* (*C. vibrioides*) CB15 and 221 *Caulobacterales* species genome representatives (out of the 347 species representatives of the "core dataset" and the additional 7 *Acaudatibacter* species representatives of the "extended dataset"; some species genome representatives lacked both 16S and 23S rRNA genes). Inferred using IQ-TREE[30] with the GTR+I+R7 model of evolution and 1000 ultrafast bootstraps (alignment length of 4266 nucleotides). The scale bar indicates number of substitutions per site in the phylogeny. Family and genus assignment follows GTDB taxonomy (R207)[31], with minor modifications according to the taxonomic proposals outlined in **Supplementary Notes 1–2**.

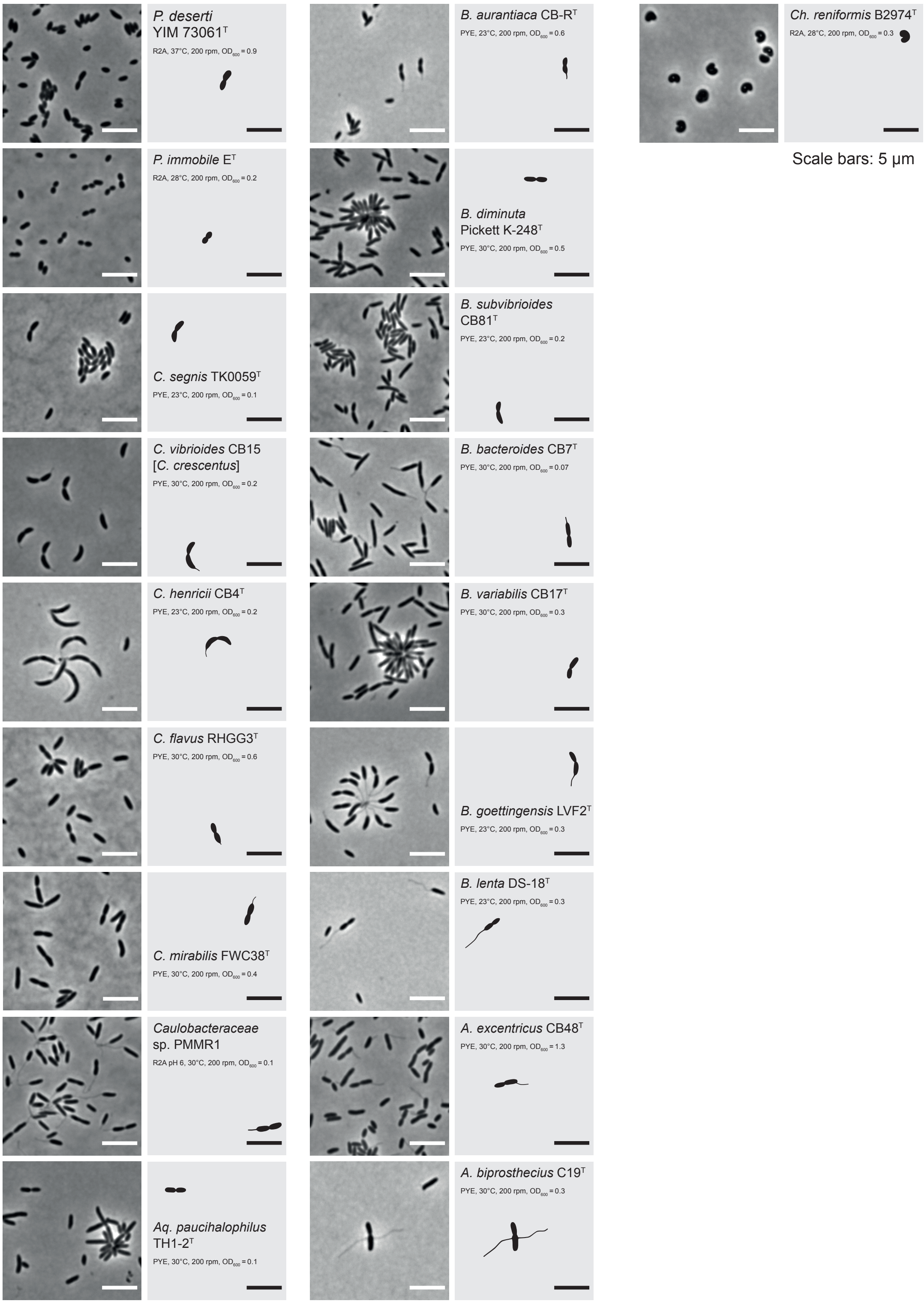

Figure S4 | Micrographs used as reference for cell outline illustrations. Representative micrographs (from one experiment each) used for schematic drawings in **Figs. 1a** and **4d**, of cells sampled at the specified growth conditions. Scale bars: 5 µm.

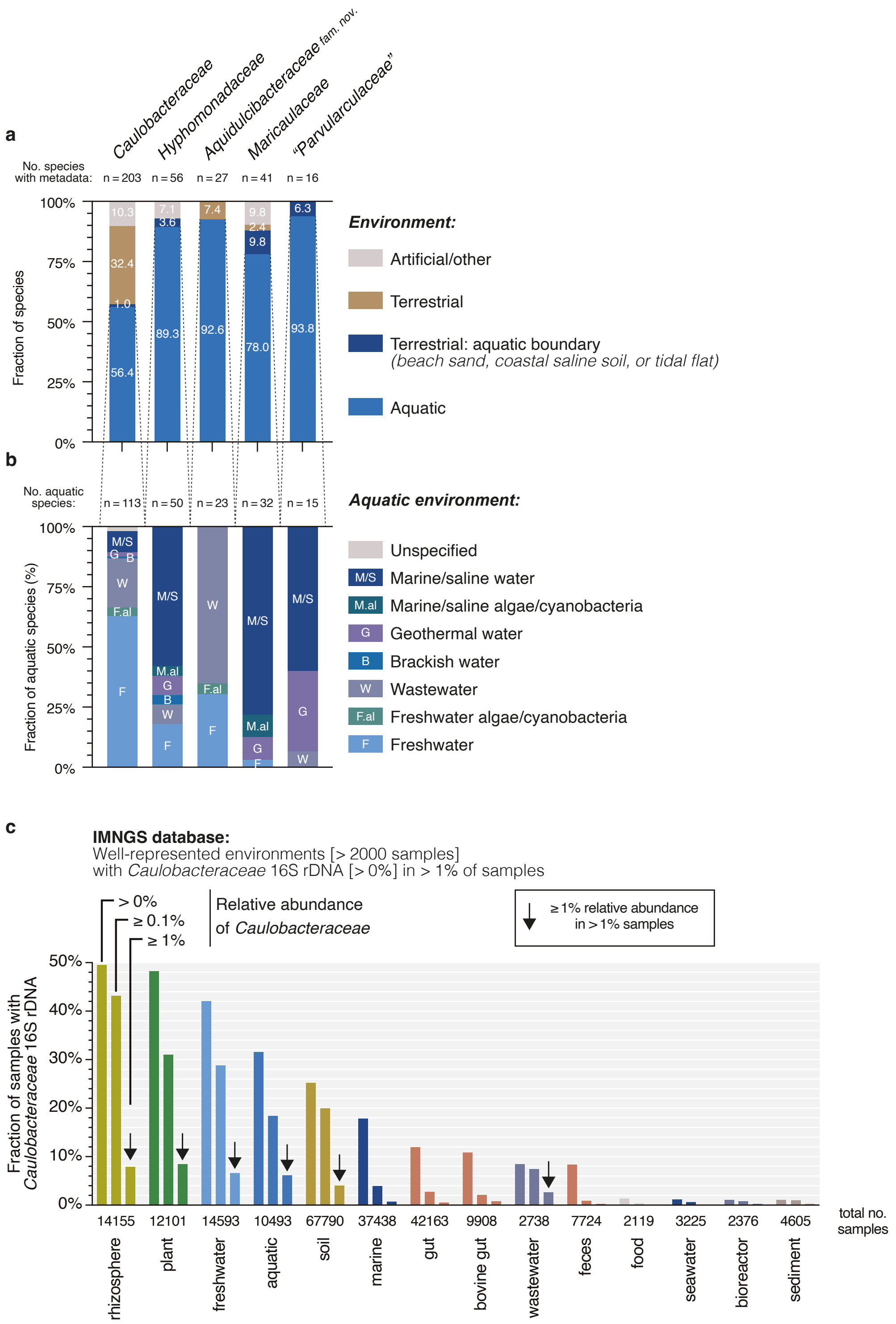

Figure S5 | Environmental distribution of *Caulobacterales* species. (a) Proportion of aquatic to terrestrial sampling environmental sources, shown in **Fig. 1b**, among genomes of the five order-*Caulobacterales* families. “Artificial/other” includes environments that fit neither category. The category “Terrestrial: aquatic boundary”, shown here, includes the terrestrial environments “beach sand”, “coastal saline soil”, and “tidal flat” and was made to emphasize that lineages classified as terrestrial in **Fig. 1b** for families *Hyphomonadaceae*, *Maricaulaceae* and “*Parvularculaceae*” are

stemming almost exclusively from coastal habitats, in close proximity to marine water. Source data are provided as a Source Data file. **(b)** Proportion of specific aquatic environmental sources among of the five order-*Caulobacterales* families. See **Supplementary Data 2** for keyword definitions and original metadata for (a) and (b). Source data are provided as a Source Data file. **(c)** The top environmental categories with *Caulobacteraceae* representation among 16S rRNA gene amplicon studies listed in the IMNGS[32] database. Well-represented environmental categories (> 2000 samples) in which *Caulobacteraceae* sequences were present at any abundance (> 0%) in at least 1% of all samples from that category. Environmental categories are sorted from left to right by the percentage for "any abundance" (> 0%). Arrows highlight environmental categories where *Caulobacteraceae* reads represent ≥ 1% of all reads in ≥ 1% of all samples; i.e., the environmental categories where *Caulobacteraceae* species are generally at high relative abundances. See **Supplementary Data 3** for full dataset. Source data are provided as a Source Data file.

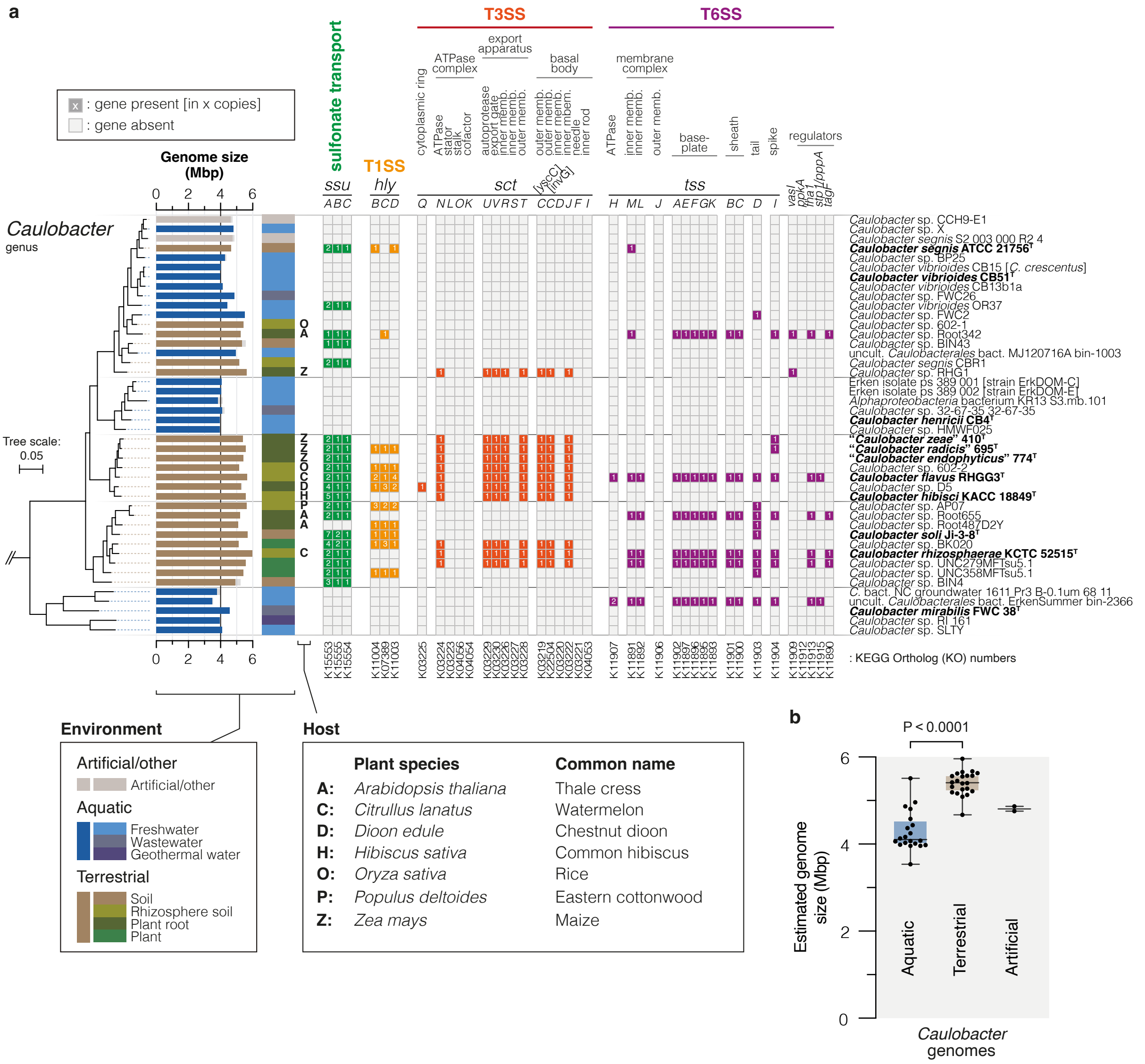

Figure S6 | Environmental specialization of *Caulobacter* lineages. (a) Overview of the phylogeny (pruned version of tree shown in **Supplementary Fig. S1**), genome size (estimated genome size calculated using estimated completeness scores from CheckM[33] 'lineage_wf' is shown in gray), and sampling site environment for *Caulobacter* species, shown alongside the presence (and copy number) and absence of putative host colonization and interaction genes (KEGG orthologs [KOs] annotated using eggNOG-mapper). For plant-associated species, the plant host species is indicated with uppercase letters when reported. Species type strains are indicated with bold letters. The *sct* and *tss* nomenclature is based on [34, 35]. T1SS, type I secretion system; T3SS, type III secretion system; T6SS, type VI secretion system. The full dataset can be found in **Supplementary Data 4**. **(b)** Comparison of estimated genome sizes of *Caulobacter* genomes from aquatic (n = 20), terrestrial (n = 22), and artificial (n = 2; environments that fit neither category) sources. Terrestrial *Caulobacter* genomes are generally larger than aquatic *Caulobacter* genomes (unpaired two-tailed parametric t-test; done in GraphPad Prism v.10.3.0). Individual genome sizes are represented by points, and boxplots show median (lines), interquartile ranges (boxes), as well as minimum and maximum genome sizes (bars). Source data are provided as a Source Data file.

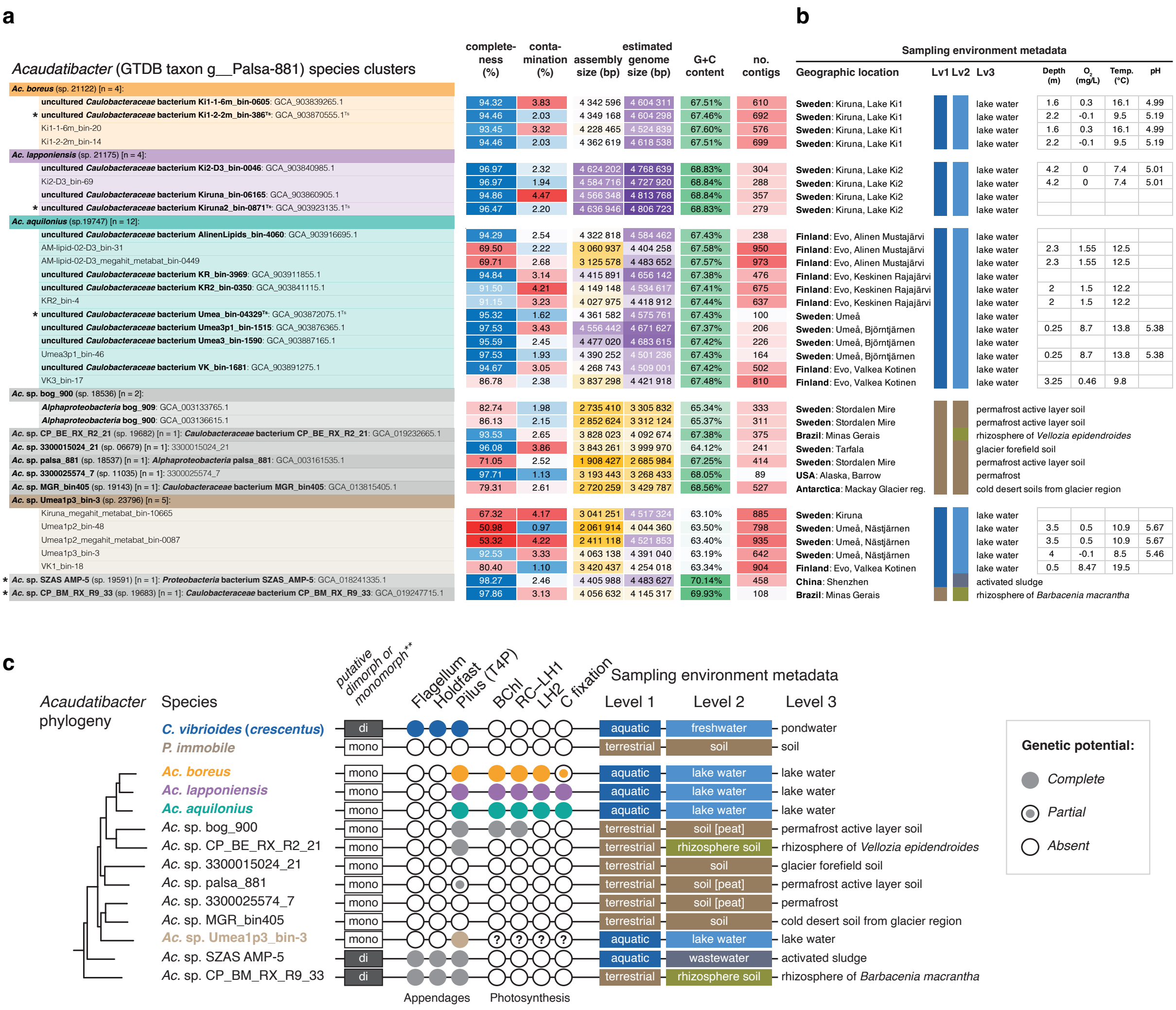

Figure S7 | Overview of the "extended dataset" of *Acaudatibacter* genomes. Overview of metadata for the subset of *Acaudatibacter* gen. nov. genomes included in Rodríguez-Gijón *et al.*[36] here referred to as the "extended dataset". **(a)** Genome attributes for the 34 *Acaudatibacter* metagenome-assembled genomes (MAGs). Taxon names are written in bold font and MAG IDs in light font. Five-digit species cluster names match the dataset of Rodríguez-Gijón *et al.*[36] Numerical values are highlighted with linear color scales to aid contrast. *: MAGs used as species genome representatives in our "core dataset". **(b)** Metagenome sampling site metadata based on NCBI BioSample information, and from Nayfach *et al.*[37] for assemblies 3300015024_21 and 3300025574_7, presented as done in **Fig. 1b**. See panel (c) for environment color codes. Metadata are presented in the right-hand table for MAGs obtained from individual freshwater metagenomes[38] for which water chemistry measurements are available. **(c)** Summary of the phylogenetic relationship, genetic potential, and habitats of the twelve *Acaudatibacter* species clusters, alongside *C. crescentus* and *P. immobile*. **: putative dimorphic or monomorphic lifecycles is inferred from the presence or large absence of developmental regulators of the dimorphic lifecycle, respectively (**Fig. 2b**, **Supplementary Fig. S8**), and the reproductive symmetry of *P. immobile* (**Fig. 3**). Circles represent genetic potential shown in **Supplementary Figs. S8** and **S16**, and **Supplementary Data 8–9**. Full phylogeny is presented in **Supplementary Fig. S9**.

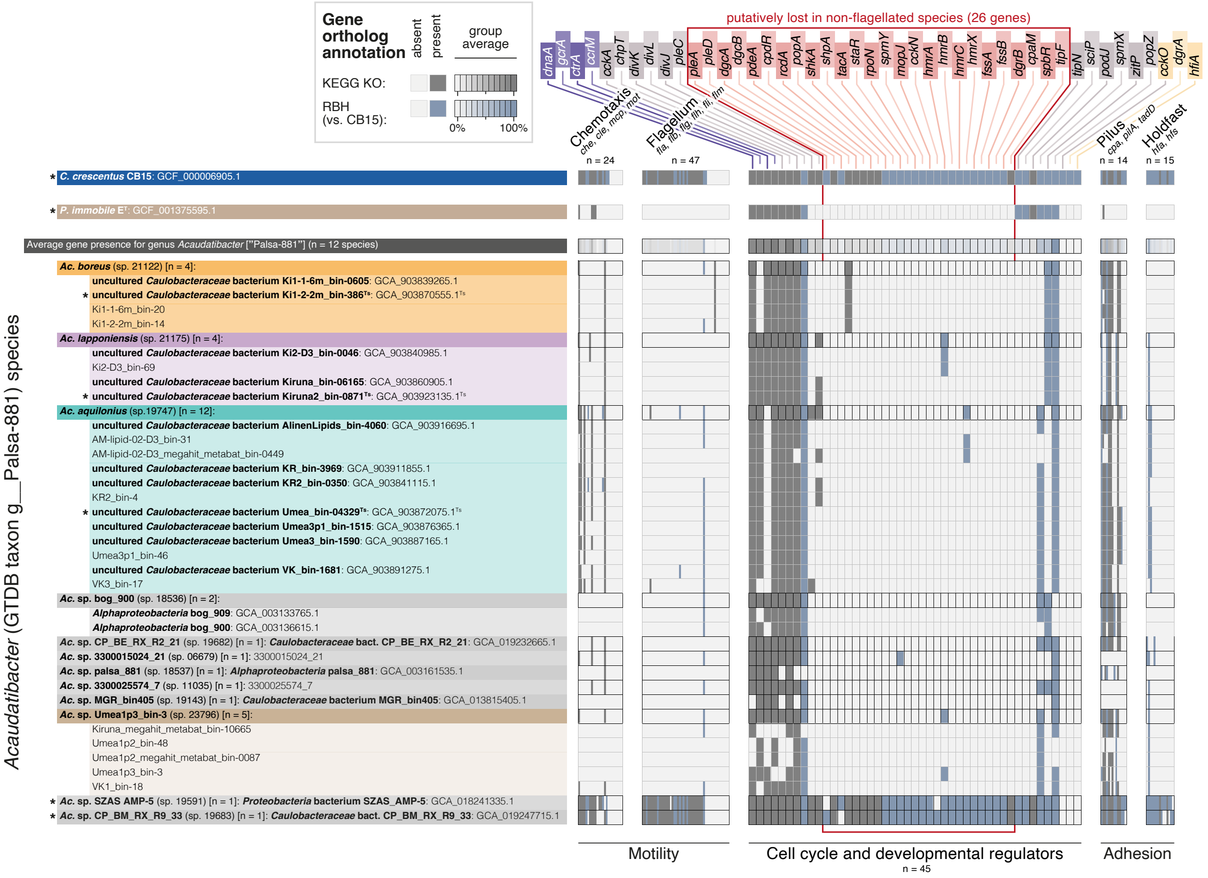

Figure S8 | Absence of cellular dimorphic traits across *Acaudatibacter* gen. nov. Presence and absence of genes shown in **Fig. 2b** for cellular dimorphism among additional *Acaudatibacter* genomes, here referred to as the "extended dataset" (see **Supplementary Fig. S7**). Gene presence and absence was determined using KEGG KO annotations (dark gray; eggNOG-mapper[39] v2.1.12 for *Acaudatibacter* genomes and eggNOG-mapper[39] v2.1.5 for *C. crescentus* and *P. immobile*) and reciprocal best blast hits to the *C. crescentus* CB15 proteome (blue). For *Acaudatibacter* species clusters represented by more than one genome assembly, the combined presence and absence is shown with black borders alongside individual genomes with gray borders. Taxon names are written in bold font and metagenome assembled genome (MAG) IDs in light font. Species clusters have been given five-digit numbers according to Rodríguez-Gijón *et al.*[36] and MAGs used as representatives of species clusters in the "core dataset" are marked with asterisks. The full dataset is found in **Supplementary Data 4**.

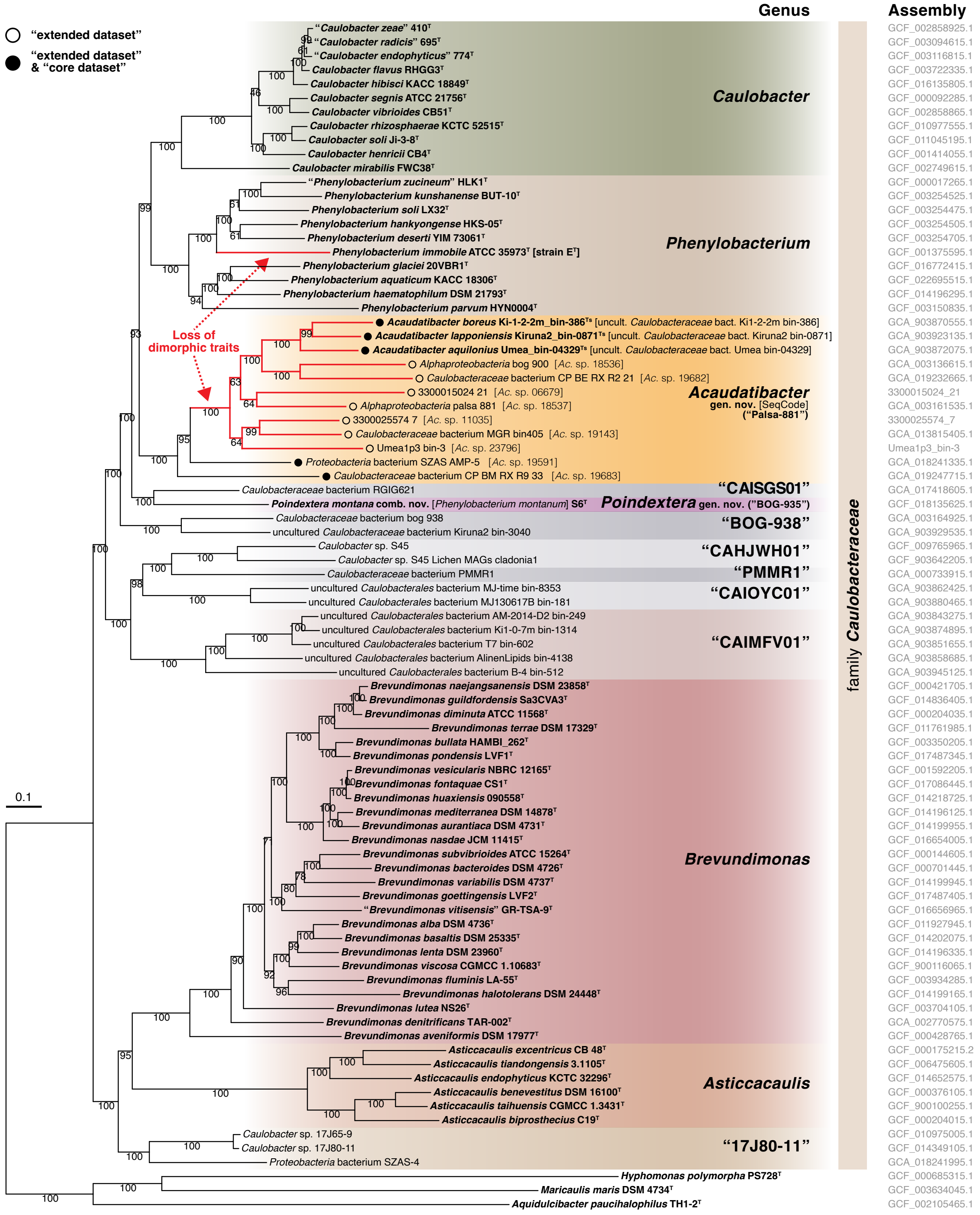

Figure S9 | Species phylogeny of *Acaudatibacter* gen. nov. Maximum likelihood concatenated species phylogeny of 117 alphaproteobacterial single-copy marker genes retrieved using GToTree v1.8.6[24] and inferred using IQ-TREE[30] with the LG+C60+F+G model of evolution and 1000 ultrafast bootstraps (alignment length of 23,561 amino acids). The following genomes were included: (i) species representatives of the twelve *Acaudatibacter* species clusters from the "extended dataset"

(**Supplementary Figs. S7a,b**) (circles), (ii) all 54 *Caulobacteraceae* species type strains of the "core dataset", (iii) the 16 species representatives from uncharacterized *Caulobacteraceae* genera of the "core dataset", (iv) and three outgroup *Caulobacterales* species type strains for rooting. Lineages with extensive absence (putative loss) of genes for dimorphic traits (see **Supplementary Fig. S8**) are highlighted in red. The scale bar indicates number of substitutions per site in the phylogeny.

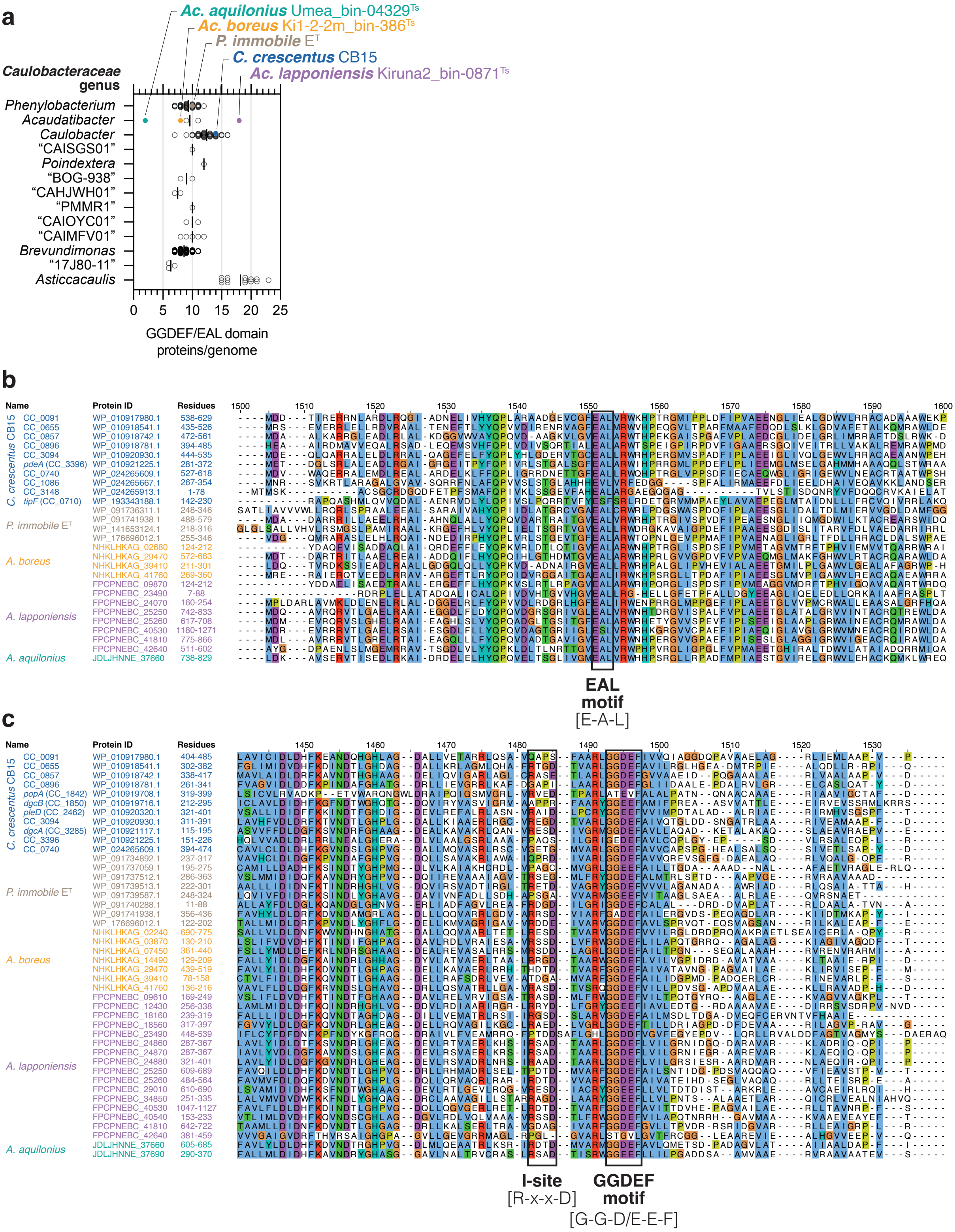

Figure S10 | Genomes of putatively monomorphic *Caulobacteraceae* species encode canonical c-di-GMP enzymes. (a) The number of GGDEF (c-di-GMP biosynthesis) and/or EAL domain (c-di-GMP degradation) proteins per "core dataset" genome, shown for *Caulobacteraceae* genera (no. genomes: *Phenylobacterium*, n = 41; *Acaudatibacter* gen. nov., n = 5; *Caulobacter*, n = 44; "CAISGS01", n = 1;

Poindextera gen. nov., n = 1; "BOG-938", n = 2; "CAHJWH01", n = 2; "PMMR1", n = 1; "CAIOYC01", n = 2; "CAIMFV01", n = 5; *Brevundimonas*, n = 86; "17J80-11", n = 3; *Asticcacaulis*, n = 14). Putatively monomorphic species are highlighted in color alongside the model strain *C. crescentus* CB15. Black vertical lines depict mean counts for each genus. Protein domains were identified using InterProScan[40]. Source data are provided as a Source Data file. **(b–c)** Proteins containing EAL (b) or GGDEF (c) domains aligned using Clustal Omega[20]. Most proteins show canonical, non-degenerate EAL (Glu-Ala-Leu) or GGDEF (Gly-Gly-Asp/Glu-Glu-Phe) motifs, with most GGDEF domain proteins additionally containing the allosteric I-site motif (Arg-x-x-Asp)—key amino acid residues involved in the catalysis of c-di-GMP degradation or biosynthesis[41]. Non-functional c-di-GMP biosynthesis enzymes typically contain degenerate GGDEF motifs[41].

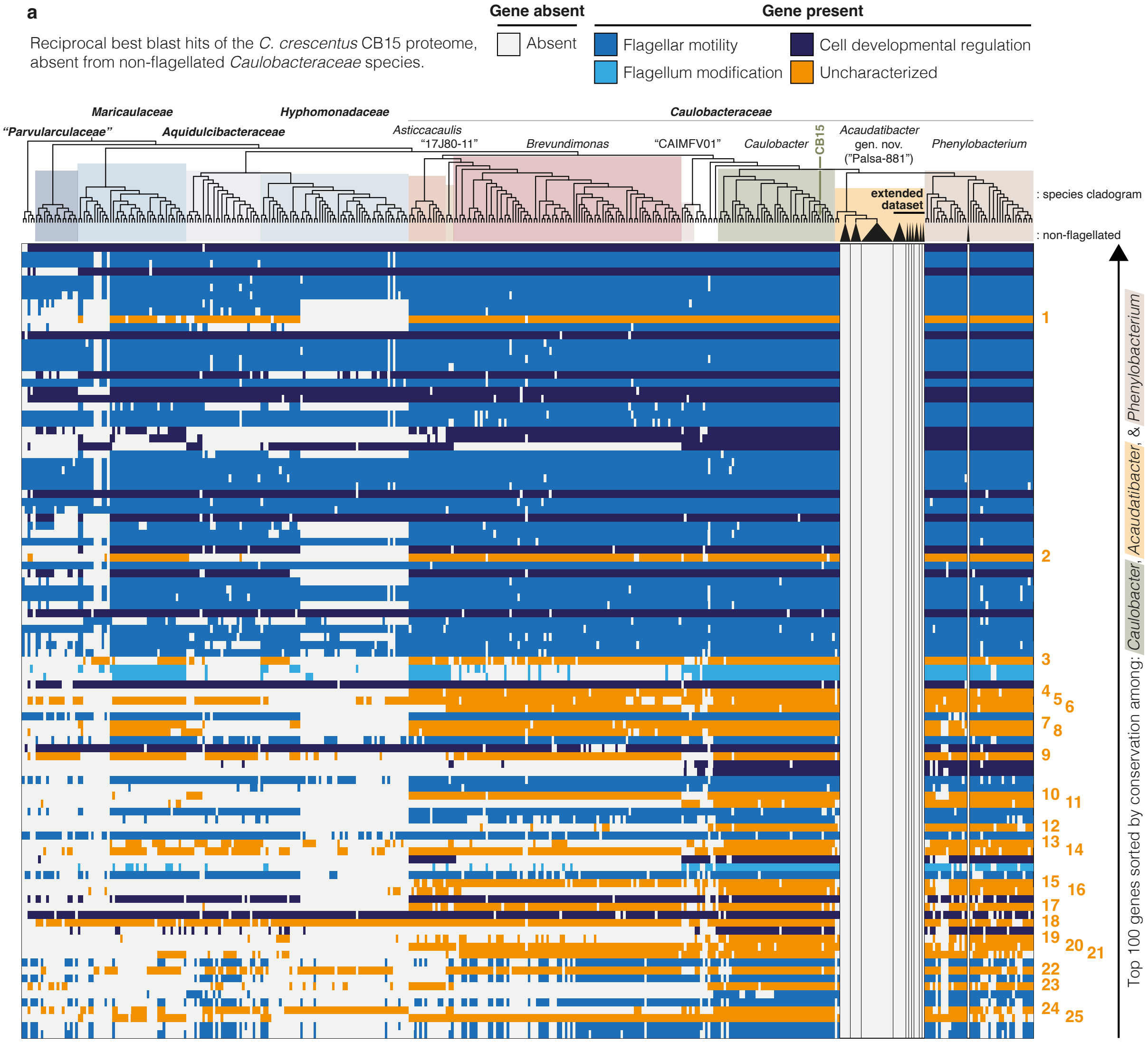

b

Conservation among flagellated species

#	Protein accession	Locus ID in CB15 (CC_RS number)	(CC number)	Locus ID in NA1000 (CCNA number)	Description	*Caul. + Acau. + Phen.* (n = 86)*	*Caulobacteraceae* (n = 203)*	*Caulobacterales* (n = 344)*
1	WP_024265705.1	CC_RS07140	CC_1362	CCNA_01424	hypothetical protein	100.0%	99.5%	77.3%
2	WP_010920121.1	CC_RS11790	CC_2260	CCNA_02343	pentapeptide repeat-containing protein	98.8%	96.6%	66.3%
3	WP_024265667.1	CC_RS05705	CC_1086	CCNA_01140	EAL domain-containing protein	97.7%	89.2%	62.5%
4	WP_010918396.1	CC_RS02650	CC_0508	CCNA_00542	hypothetical protein	96.5%	95.1%	56.1%
5	WP_010919355.1	CC_RS07765	CC_1481	CCNA_01548	MFS transporter	96.5%	88.2%	75.9%
6	WP_024265917.1	CC_RS16500	CC_3162	CCNA_03265	response regulator	96.5%	86.7%	51.2%
7	WP_010918125.1	CC_RS01220	CC_0236	CCNA_00236	hypothetical protein	95.3%	96.6%	67.4%
8	WP_012640579.1	CC_RS14430	CC_2764	CCNA_02852	hypothetical protein	95.3%	94.1%	64.8%
9	WP_010920363.1	CC_RS13085	CC_2506	CCNA_02592	acyl-CoA thioesterase	95.3%	83.7%	71.5%
10	WP_010918801.1	CC_RS04795	CC_0917	CCNA_00965	DnaJ domain-containing protein	94.2%	92.1%	56.4%
11	WP_012640241.1	CC_RS07575	CC_1443	CCNA_01510	hypothetical protein	94.2%	44.3%	27.3%
12	WP_010920365.1	CC_RS13090	CC_2508	CCNA_02593	hypothetical protein	93.0%	43.8%	25.9%
13	WP_010919908.1	CC_RS10650	CC_2044	CCNA_02124	DUF3429 domain-containing protein	91.9%	45.8%	39.5%
14	WP_012640303.1	CC_RS08860	CC_1694	CCNA_01765	hypothetical protein	90.7%	91.6%	73.0%
15	WP_010918665.1	CC_RS04070	CC_0780	CCNA_00821	hypothetical protein	89.5%	85.7%	50.6%
16	WP_010918239.1	CC_RS01810	CC_0350	CCNA_00355	pentapeptide repeat-containing protein	88.4%	45.8%	28.8%
17	WP_024265624.1	CC_RS04075	CC_0781	CCNA_00822	hypothetical protein	87.2%	86.2%	51.2%
18	WP_012640450.1	CC_RS11770	NA	CCNA_02339	class I SAM-dependent methyltransferase	86.0%	90.6%	91.9%
19	WP_010919384.1	CC_RS07920	CC_1510	CCNA_01578	BlaI/MecI/CopY family transcriptional regulator	84.9%	47.8%	30.5%
20	WP_024265755.1	CC_RS10495	CC_2012	CCNA_02091	hypothetical protein	83.7%	90.6%	53.5%
21	WP_010919892.1	CC_RS10565	CC_2026	CCNA_02105	hypothetical protein	83.7%	81.8%	54.9%
22	WP_010921320.1	CC_RS18220	CC_3491	CCNA_03605	DUF2219 family protein	82.6%	80.3%	69.2%
23	WP_010919208.1	CC_RS06980	CC_1331	CCNA_01392	hypothetical protein	82.6%	44.8%	31.7%
24	WP_024265607.1	CC_RS03825	CC_0736	CCNA_00773	M20 family peptidase	81.4%	40.9%	50.0%
25	WP_010918950.1	CC_RS05605	CC_1066	CCNA_01119	hypothetical protein	80.2%	91.6%	63.7%

Figure S11 | Legend on next page.

Figure S11 | Uncharacterized *C. crescentus* genes that might play roles in flagellar motility and dimorphism, based on their specific absence in lineages with extensive lack of genes for dimorphic traits. (a) *C. crescentus* CB15 protein-coding gene orthologs identified using the reciprocal best blast hit (RBH) algorithm in *Caulobacterales* genomes, but being undetected across *Caulobacteraceae* genomes with large absences of flagellar and developmental genes (n = 33; *P. immobile* and 32 *Acaudatibacter* MAGs). Genes (rows) are sorted from top to bottom by their conservation among genomes of the *Acaudatibacter–Caulobacter–Phenylobacterium* superclade encoding flagellar genes—showing the top 100 genes (out of 3827 *C. crescentus* genes) from this sorting. The genomes included are the following: (i) the "core dataset" comprising the genome representatives of the 347 *Caulobacterales* species clusters, the *C. crescentus* CB15 genome, and five genomes of outgroup *Alphaproteobacteria*, and (ii) the "extended dataset" with the additional 29 *Acaudatibacter* gen. nov. (GTDB taxon g__Palsa-881) genomes (see also **Supplementary Fig. S7**). For the "core dataset", a cladogram displaying the species tree topology from **Fig. 1a** is shown. Some *Acaudatibacter* species clusters of the "extended dataset" are not represented in this species phylogeny. Since some putatively non-flagellated species clusters are represented by several genomes, each is highlighted with black triangles and are outlined in black in the presence–absence heatmap. Uncharacterized genes are numbered and highlighted in orange. The full dataset can be found in **Supplementary Data 6**. **(b)** Names and annotations of the 25 genes uncharacterized genes from panel (a). For each gene, the proportion of flagellar gene-containing genomes in which an ortholog was identified using the RBH method, is shown as percentages for the *Acaudatibacter–Caulobacter–Phenylobacterium* clade (n = 86 species clusters), the family *Caulobacteraceae* (n = 203 species clusters), and the order *Caulobacterales* (n = 344 species clusters).

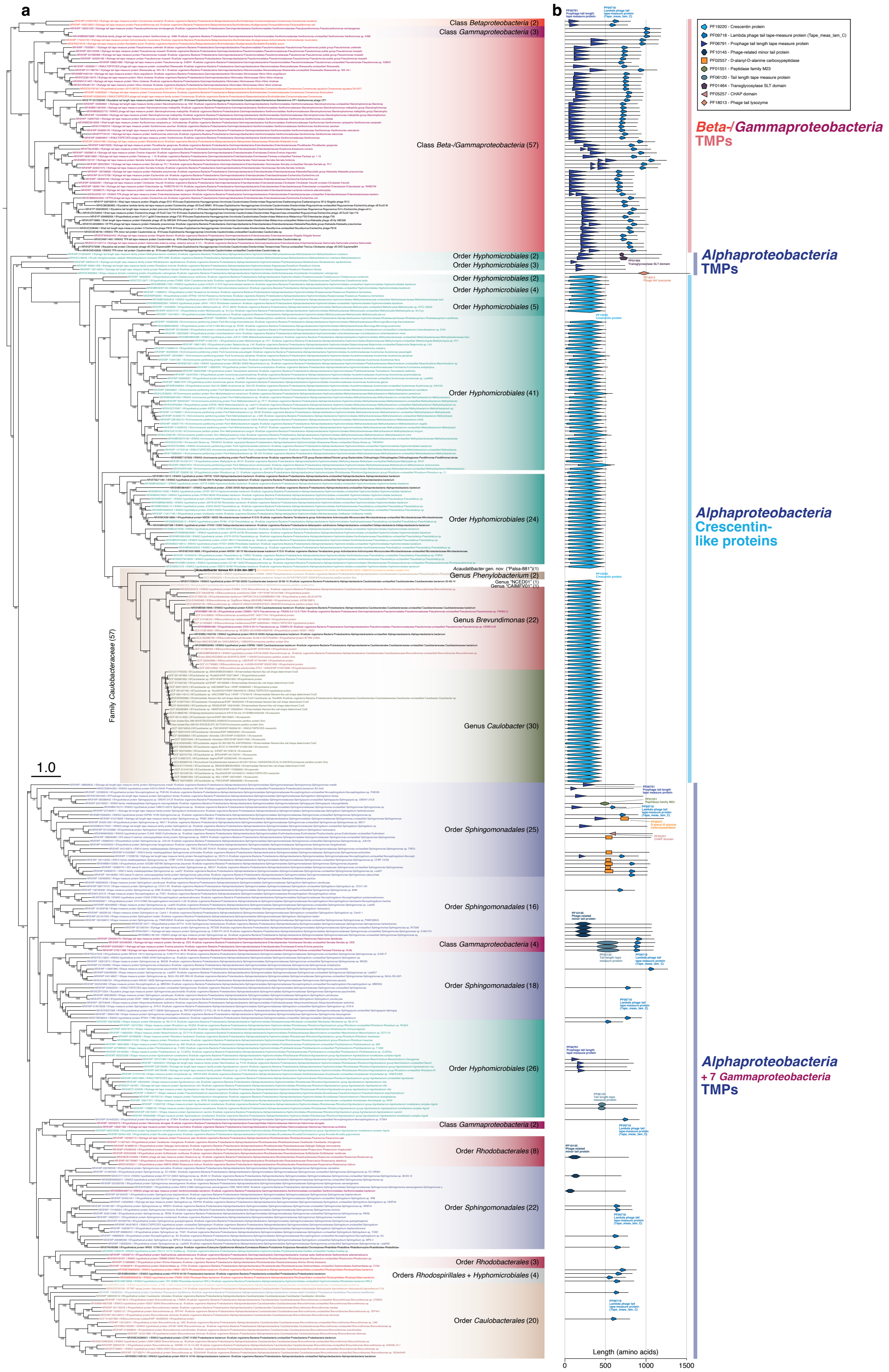

Figure S12 | Legend on next page.

Figure S12 | Phylogeny of all identified crescentin (CreS) homologs. (a) ML phylogeny of crescentin (CreS) homologs. The phylogeny was inferred using IQ-TREE[30] with the Q.pfam+C60+R9 model of evolution and 1000 ultrafast bootstraps (356 sequences with 902 amino acid positions), and was rooted with the major *Beta-/Gammaproteobacteria* clade (NCBI taxonomy). The scale bar indicates number of substitutions per site. Same tree as shown in **Fig. 4c**. **(b)** Pfam protein domains identified for each protein included in (a), mapped over the length of the protein. The representation of the different Pfam domains is outlined in the right-most upper panel. Proteins without predicted domains are not shown. For collapsed clades in **Fig. 4c**, the major taxonomic group is specified alongside the numbers of sequences per clade shown in parentheses.

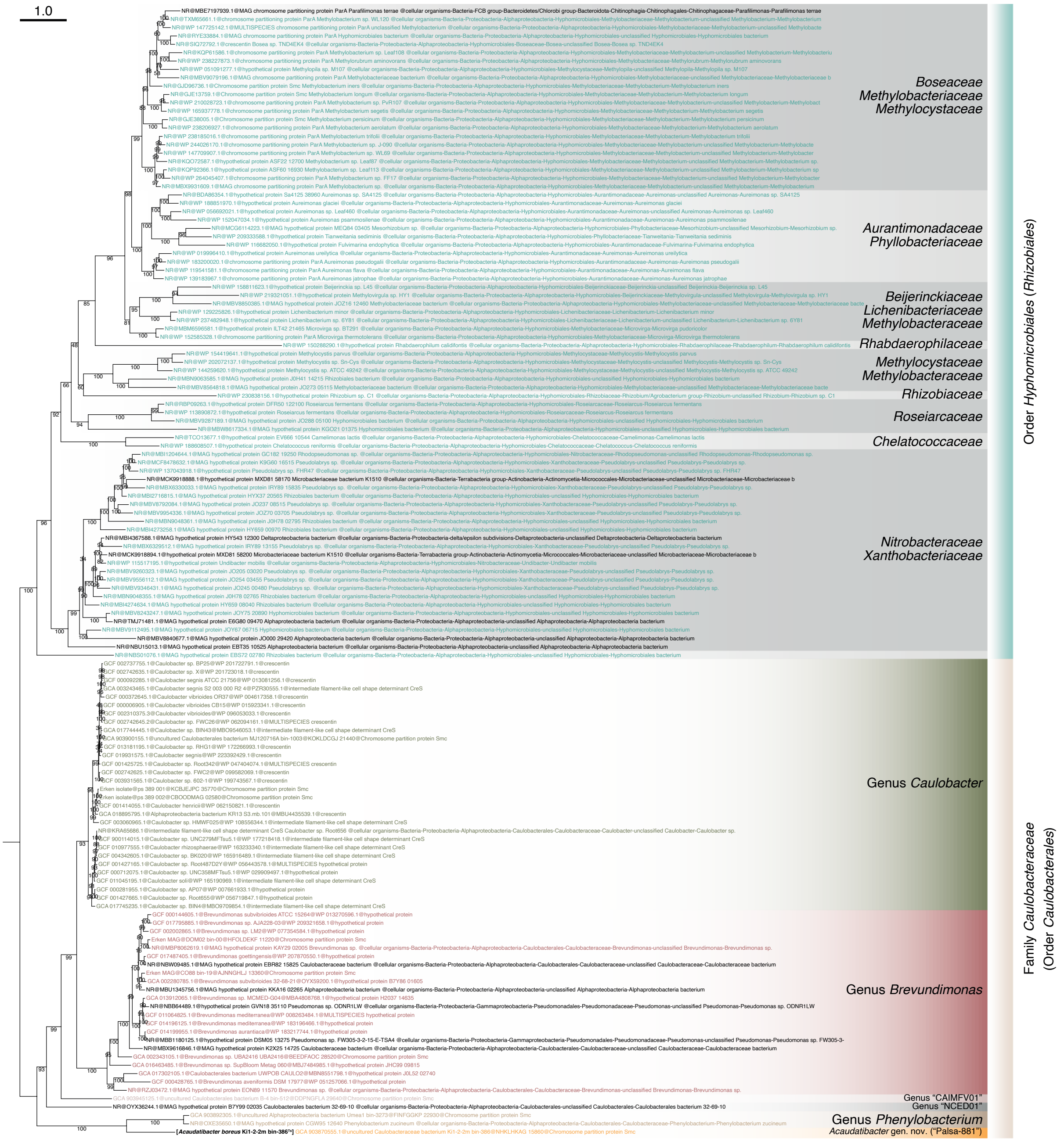

Figure S13 | Phylogeny of the subset of 'crescentin-like' crescentin (CreS) homologs. ML phylogeny including only the clade from **Fig. 4c** and **Supplementary Fig. S12** where proteins with predicted crescentin domains (PF19220) are found. The phylogeny was inferred using IQ-TREE[30] with the JTT+C60+R5 model of evolution and 1000 ultrafast bootstraps (135 sequences with 407 amino acid alignment positions), and was rooted between *Hyphomicrobiales* and *Caulobacteraceae*. The scale bar indicates number of substitutions per site. *Hyphomicrobiales* families and *Caulobacteraceae* genera are indicated. Same tree as shown in **Fig. 4d**.

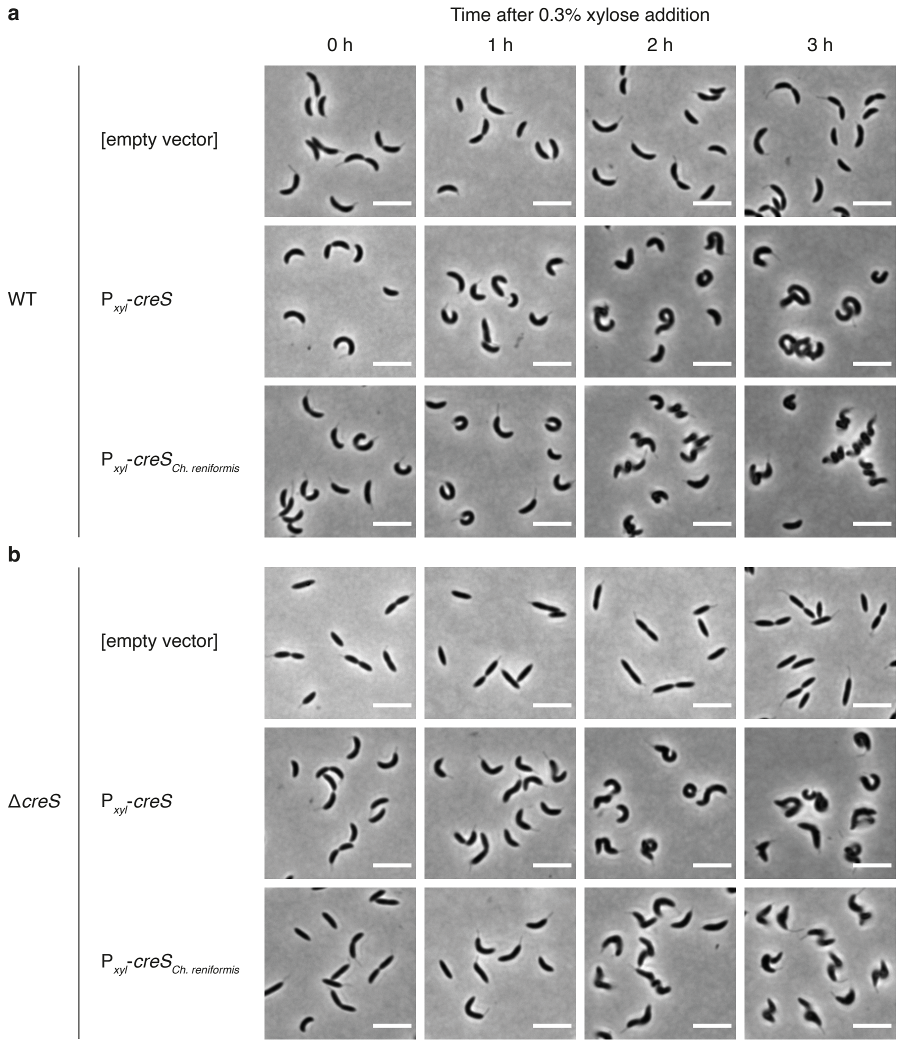

Figure S14 | Expression of a CreS homolog from *Chelatococcus reniformis* increases cell curvature in wild-type *C. crescentus* NA1000 cells and is able to restore cell curvature of a Δ*creS* mutant of *C. crescentus* NA1000. Representative micrographs (from one experiment) of wild-type cells **(a)** and Δ*creS* cells **(b)** containing pBXMCS-4 plasmid constructs and grown until mid-exponential phase in PYE broth with gentamicin, at which point xylose was added to a final concentration of 0.3% to induce expression from the P_{xyl} promoter. Scale bars: 5 µm.

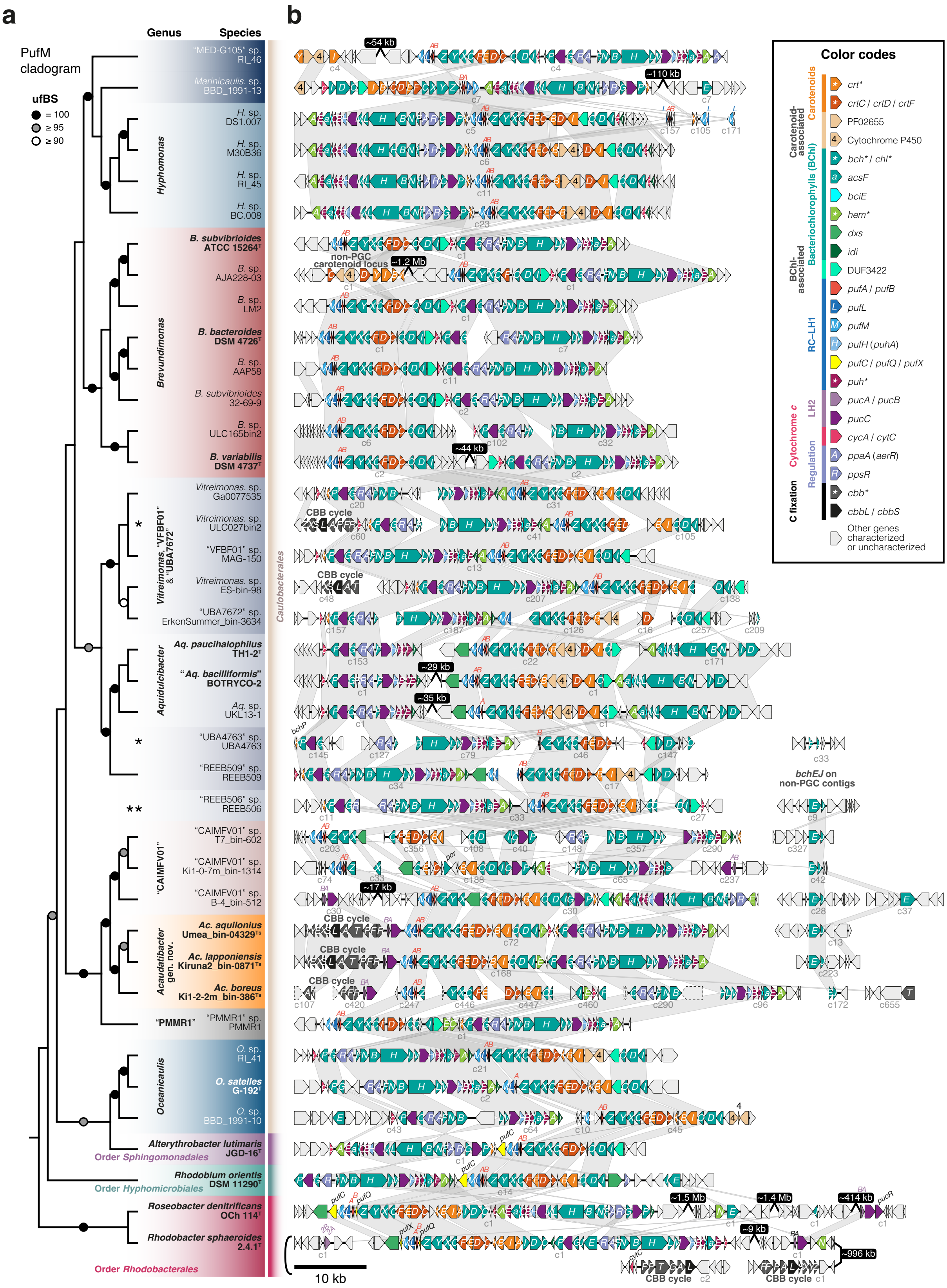

Figure S15 | Legend on next page.

Figure S15 | Gene synteny of *Caulobacterales* photosynthesis gene clusters (PGCs). (a) Cladogram representation of the PufM phylogeny shown in **Fig. 5e** and **Supplementary Fig. S17**, pruned to only include relevant species. Ultrafast bootstrap proportions (ufBPs) are indicated with circles. Single asterisks highlight species absent from the PufM phylogeny; their vertical placement here is based instead on the BchY phylogeny of **Supplementary Fig. S20**. Double asterisks highlight the taxon “REEB506” sp. REEB506, for which its PufM ortholog was placed without support outside of the main *Caulobacterales* clade (**Supplementary Fig. S17**); its vertical placement here close to the taxa “UBA4763” and “REEB509” is based instead of the BchY phylogeny of **Supplementary Fig. S20**. **(b)** Synteny of *Caulobacterales* phototrophy genes. Species have been sorted vertically following the topology of panel (a). Genes are color-coded and schematically labeled with letters corresponding to their gene names as outlined in the right-most upper panel, and orthologs are linked with gray boxes. Contigs are marked with “c” followed by the contig number and distances between loci on the same contig are shown with black boxes. When present and fully enclosed within 5 kb of a phototrophy gene, additional flanking genes are shown to highlight broader genetic context. Uncharacterized genes coding for PF02655 (ATP-grasp domain) and cytochrome P450 proteins, as well as for DUF3422 (domain of unknown function 3422) proteins, were tentatively labeled as carotenoid- and bacteriochlorophyll-associated, respectively, based on their colocalization patterns.

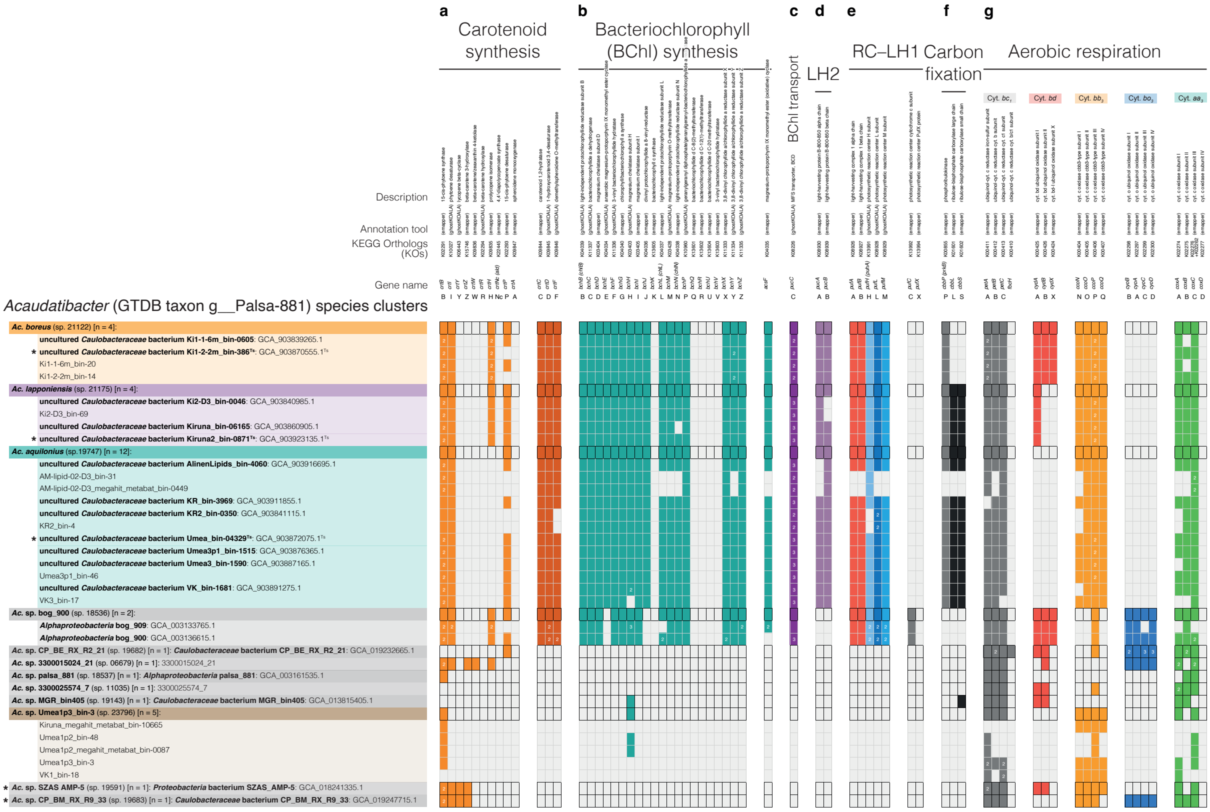

Figure S16 | Presence and absence of genes, in *Acaudatibacter* gen. nov. "extended dataset" genomes sourced from Rodríguez-Gijón *et al.*[36], for pigment production, phototrophy, carbon fixation, and aerobic respiration. (a–g) Presence (colors) and absence (light gray) of KEGG orthologs (KOs) annotated using either eggNOG-mapper[39] (emapper; v2.1.12) or GhostKOALA[42] (v3.0), involved in (a) carotenoid biosynthesis, (b) bacteriochlorophyll biosynthesis, (c) bacteriochlorophyll transport, (d) light-harvesting complex II (LH2), (e) reaction center–light-harvesting complex I (RC–LH1), (f) CO_2 fixation via the CBB cycle, and (g) aerobic respiration among *Acaudatibacter* genomes. For species clusters comprising several genomes, data for individual genomes are shown with gray borders, and the binary presence/absence of genes across all genomes of the species cluster is shown with black borders. Numbers show KO copy numbers > 1. Abbreviations: cytochrome (cyt.). The full dataset can be found in **Supplementary Data 4**.

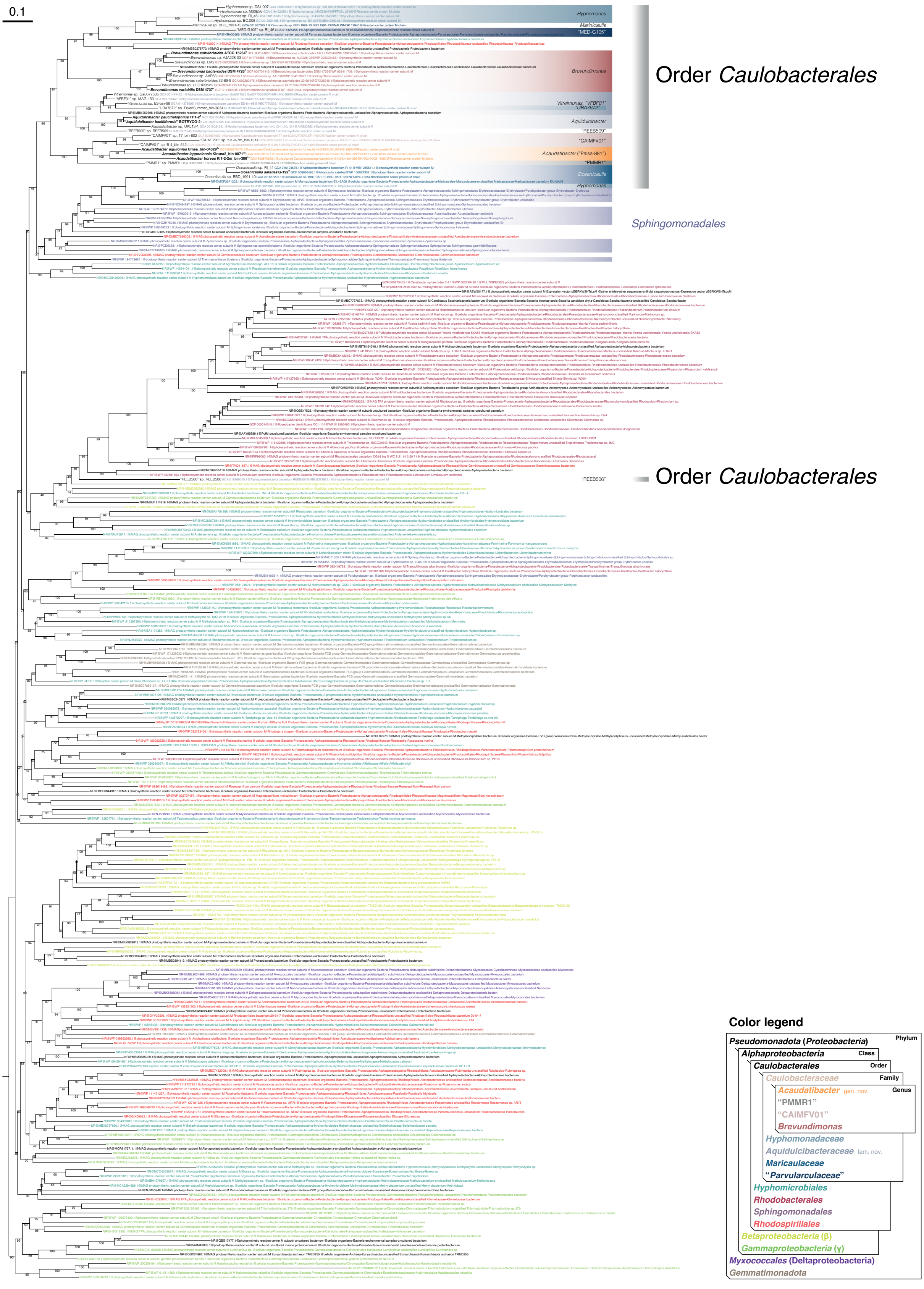

Figure S17 | Legend on next page.

Figure S17 | PufM phylogeny. ML phylogeny of photosynthetic reaction center protein PufM, inferred using IQ-TREE[30] with the WAG+C60+R9 model of evolution and 1000 ultrafast bootstraps (278 sequences with 295 amino acid alignment positions). The tree was rooted at the major *Chromatiales* (*Gammaproteobacteria*) clade. Taxonomic groups are color-coded as outlined in the color legend. The scale bar indicates number of substitutions per site. Same tree as shown in **Fig. 5e**; gradients highlight collapsed clades in **Fig. 5e**.

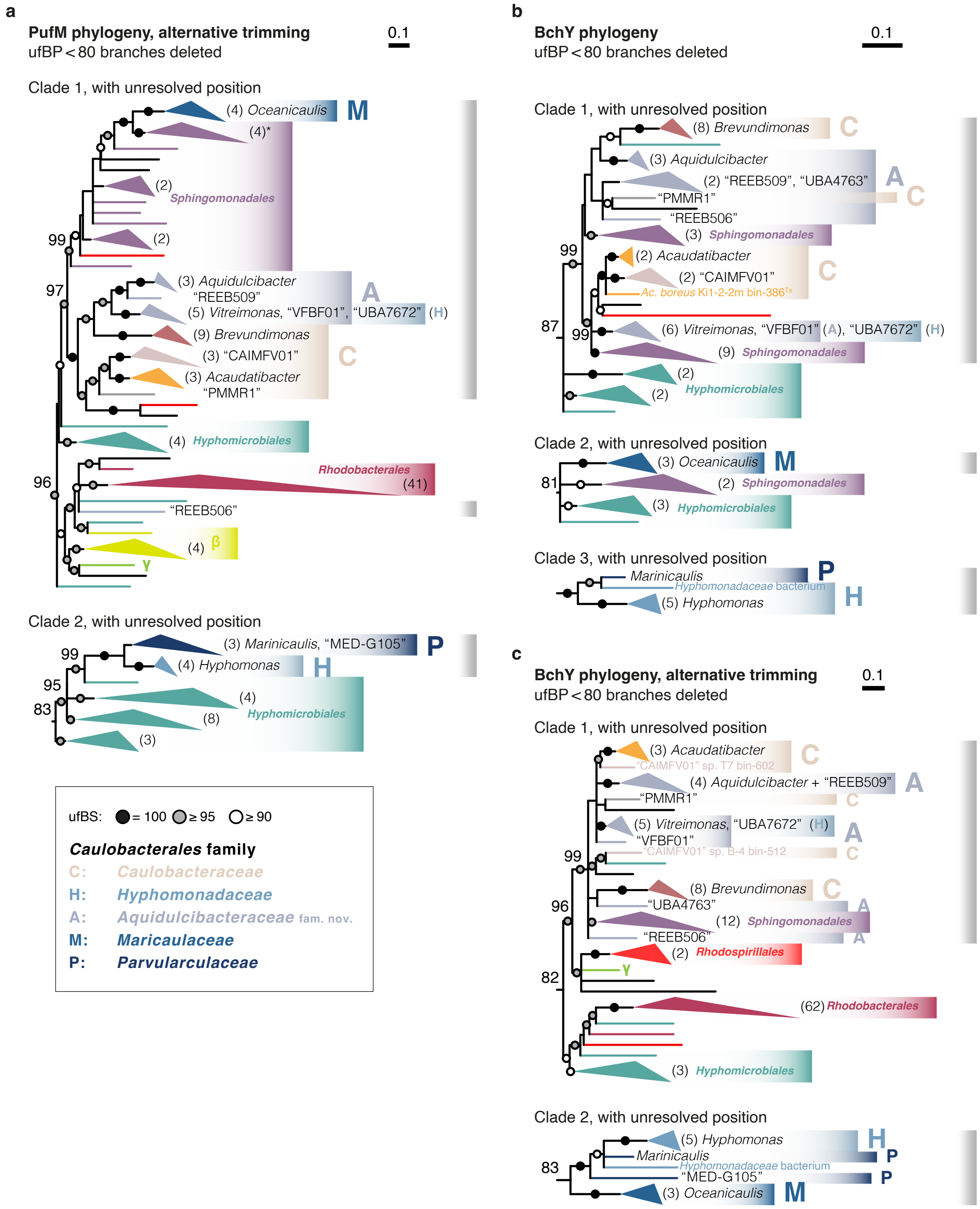

Figure S18 | Overview of *Caulobacterales*-containing clades in additional maximum-likelihood phylogenies of photosynthesis proteins. The PufM (photosynthetic reaction center subunit M) phylogeny was inferred as previously in **Fig. 5e**, but subjected to less stringent trimming. The BchY (chlorophyllide *a* reductase subunit Y) phylogenies were inferred using IQ-TREE[30] with the WAG+C60+R10 model of evolution and 1000 ultrafast bootstraps. All trees were rooted using their respective major *Chromatiales* (*Gammaproteobacteria*) clades, before unsupported branches (ultrafast bootstrap support [ufBS] < 80%) were deleted. ufBS is indicated with circles, and specified with

numbers for key branches. The scale bars indicate the number of substitutions per site. Uppercase letters denote the *Caulobacterales* families: A, *Aquidulcibacteraceae* fam. nov.; C, *Caulobacteraceae*; H, *Hyphomonadaceae*; M, *Maricaulaceae*; P, "*Parvularculaceae*". **(a)** PufM tree with less stringent alignment trimming (278 sequences with 324 amino acid alignment positions). The asterisk indicates a clade in which a second copy of PufM from *Hyphomonas* sp. DS1.007 clusters with three *Sphingomonadales* sequences. The full tree is found in **Supplementary Fig. S19**. **(b)** BchY with more stringent trimming (352 sequences with 417 amino acid alignment positions). The full tree is found in **Supplementary Fig. S20**. **(c)** BchY with less stringent trimming (352 sequences with 509 amino acid alignment positions). The full tree is found in **Supplementary Fig. S21**.

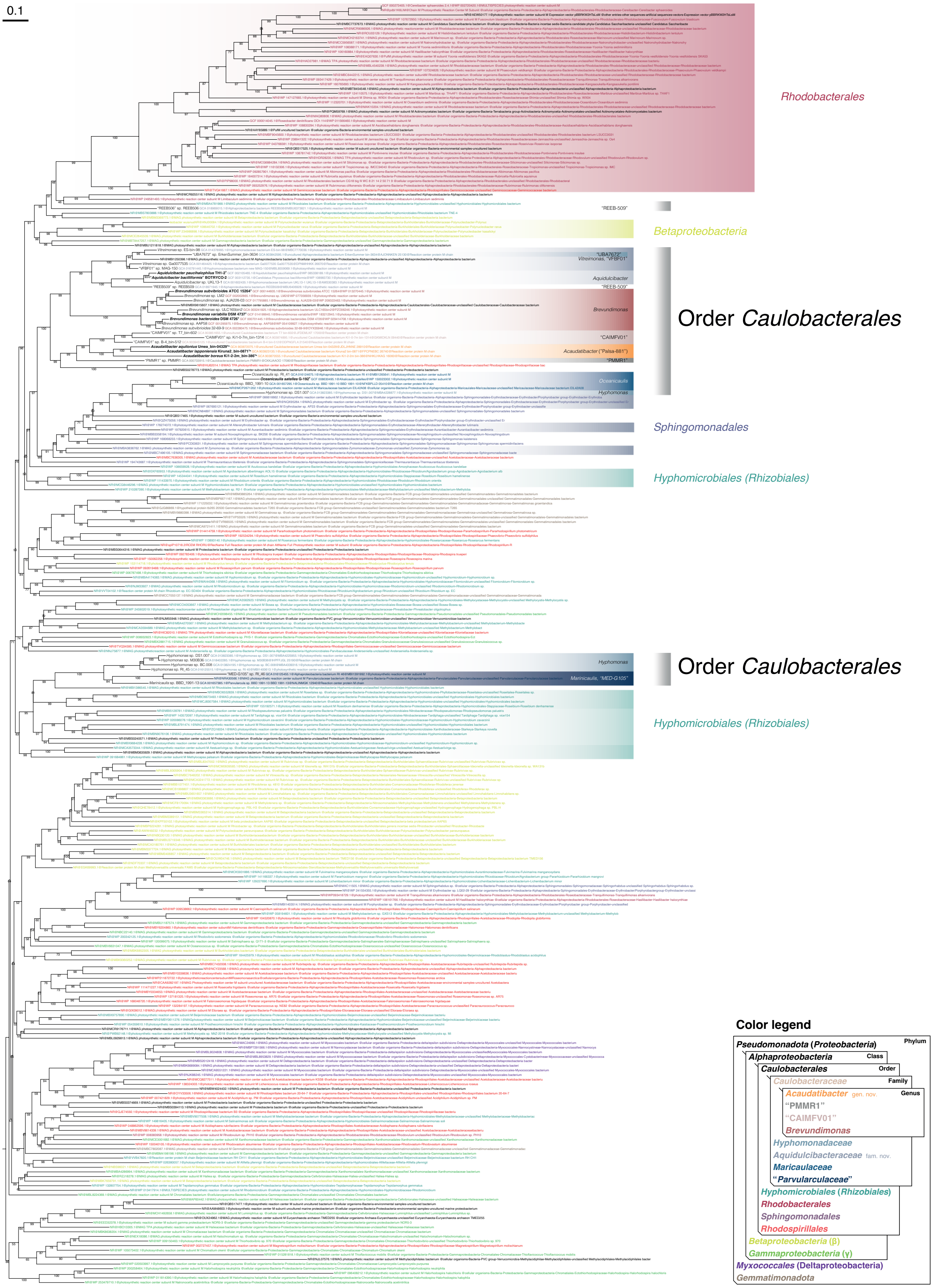

Figure S19 | Legend on next page.

Figure S19 | Alternative PufM phylogeny. ML phylogeny of photosynthetic reaction center protein PufM, inferred as in **Supplementary Fig. S17**, but with less stringent alignment trimming (278 sequences with 324 amino acid positions). The tree was rooted at the major *Chromatiales* (*Gammaproteobacteria*) clade. Taxonomic groups are color-coded as outlined in the color legend. The scale bar indicates number of substitutions per site. Same tree as shown in **Supplementary Fig. S18a**; gradients highlight collapsed clades in **Supplementary Fig. S18a**.

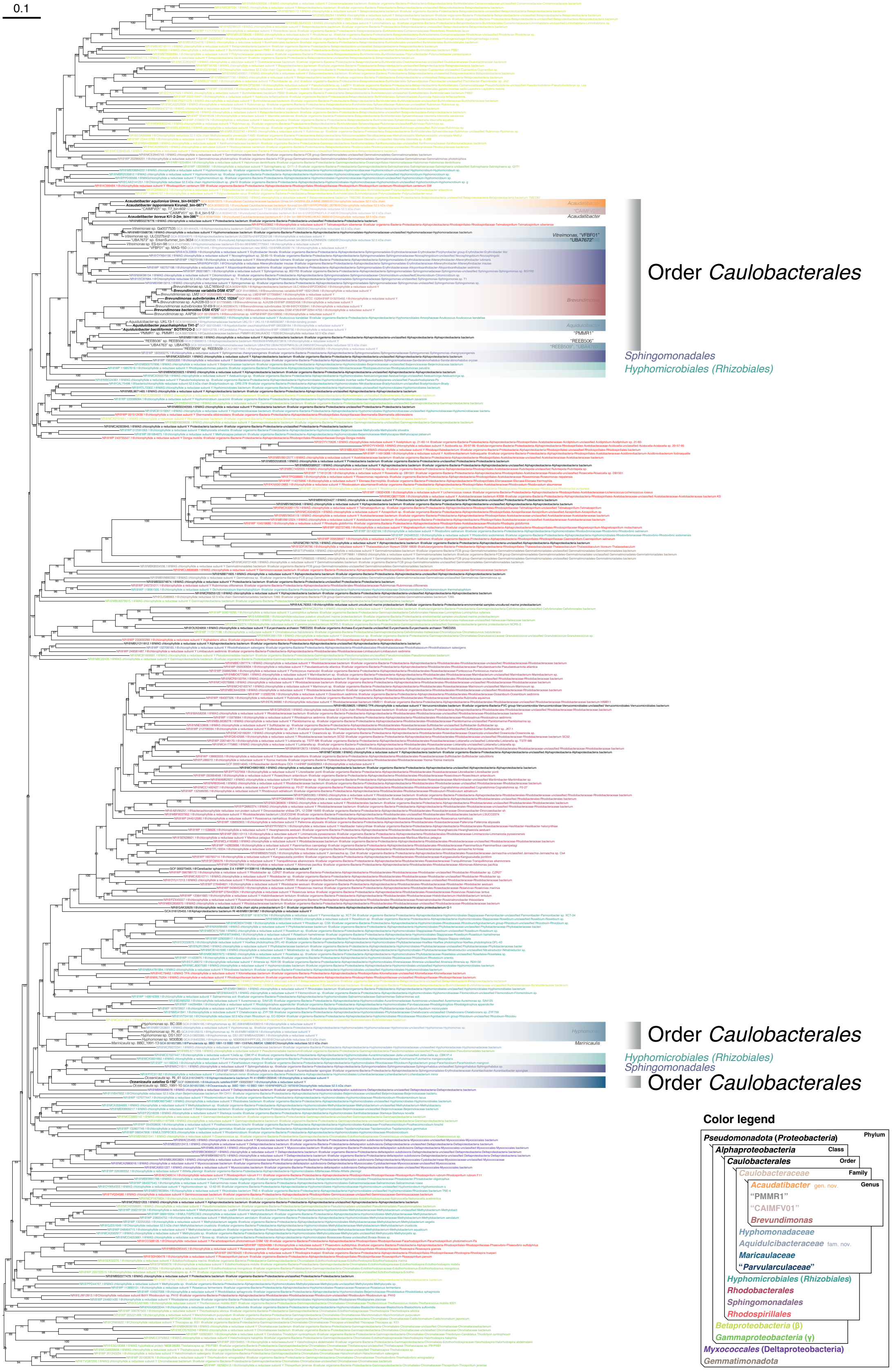

Figure S20 | Legend on next page.

Figure S20 | BchY Phylogeny. ML phylogeny of chlorophyllide *a* reductase subunit BchY, inferred using IQ-TREE[30] with the WAG+C60+R10 model of evolution and 1000 ultrafast bootstraps (352 sequences with 417 amino acid alignment positions). The tree was rooted at the major *Chromatiales* (*Gammaproteobacteria*) clade. Taxonomic groups are color-coded as outlined in the color legend. The scale bar indicates number of substitutions per site. Same tree as shown in **Supplementary Fig. S18b**; gradients highlight collapsed clades in **Supplementary Fig. S18b**.

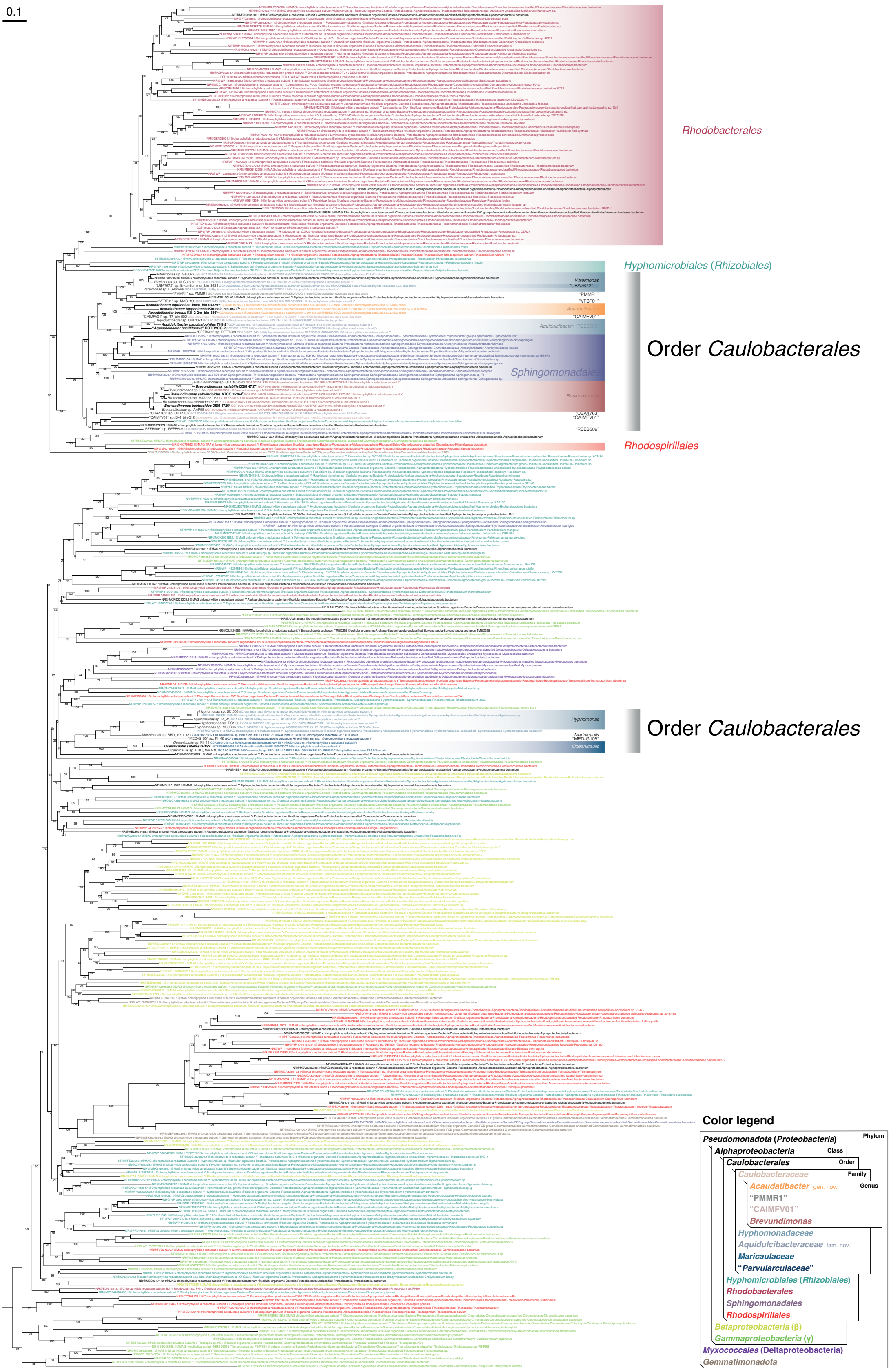

Figure S21 | Legend on next page.

Figure S21 | Alternative BchY phylogeny. ML phylogeny of chlorophyllide *a* reductase subunit BchY, inferred as in **Supplementary Fig. S17**, but with less stringent alignment trimming (352 sequences with 509 amino acid alignment positions). The tree was rooted at the major *Chromatiales* (*Gammaproteobacteria*) clade. Taxonomic groups are color-coded as outlined in the color legend. The scale bar indicates number of substitutions per site. Same tree as shown in **Supplementary Fig. S18c**; gradients highlight collapsed clades in **Supplementary Fig. S18c**.

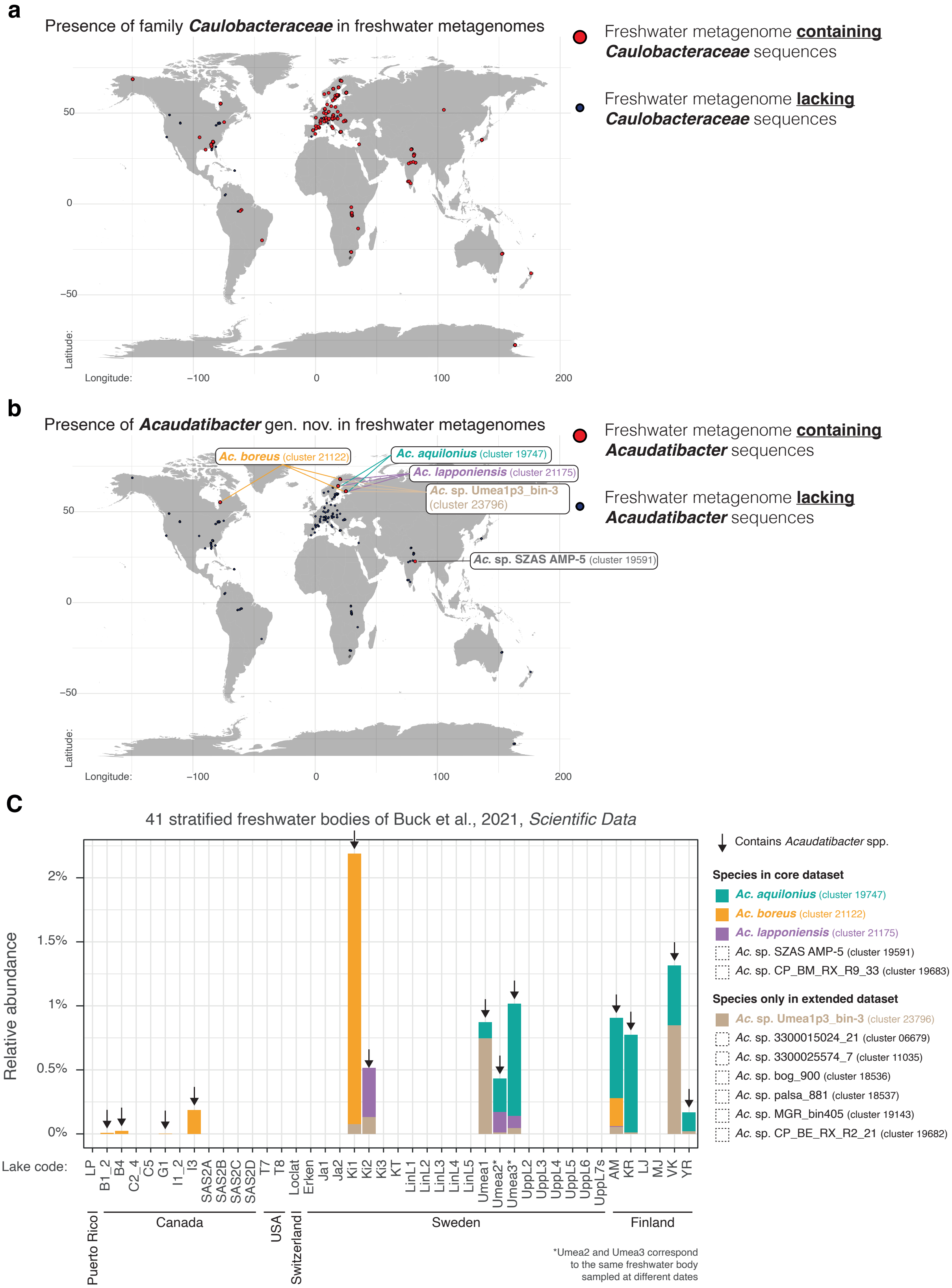

Figure S22 | Geographical distribution of freshwater *Acaudatibacter* species. (a) Competitive mapping results of *Caulobacteraceae* species clusters (genomes of > 50% completeness and < 5%

contamination, clustered at > 95% ANI) against 636 freshwater metagenomes, from Rodríguez-Gijón *et al.*[36] Out of the 325 *Caulobacteraceae* species clusters investigated, 190 were present in at least one freshwater metagenome (red circles). Dark blue circles represent metagenomes to which none of the 325 *Caulobacteraceae* species clusters mapped. Note that some locations have multiple geographically proximal freshwater bodies and/or multiple metagenomes per freshwater body, resulting in overlapping datapoints. Source data are provided as a Source Data file. **(b)** Same as in (a), but mapping only the subset of species clusters (n = 12) belonging to the genus *Acaudatibacter* gen. nov. (GTDB taxon "g__Palsa-881"). The five *Acaudatibacter* species clusters that mapped to freshwater metagenomes are indicated. Source data are provided as a Source Data file. **(c)** Total sum of relative abundances across sampled depths per water body, of the twelve *Acaudatibacter* species clusters among metagenomes from 41 stratified freshwater bodies represented in the dataset of Buck *et al.*[38] (see **Fig. 6c** and **Supplementary Fig. S24**), as a proxy for species cluster distribution. Genomes of the species clusters labeled in black did not map to the dataset. Among the twelve *Acaudatibacter* species clusters investigated, five are represented in our core dataset of 347 *Caulobacterales* species genome representatives, as indicated. Source data are provided as a Source Data file.

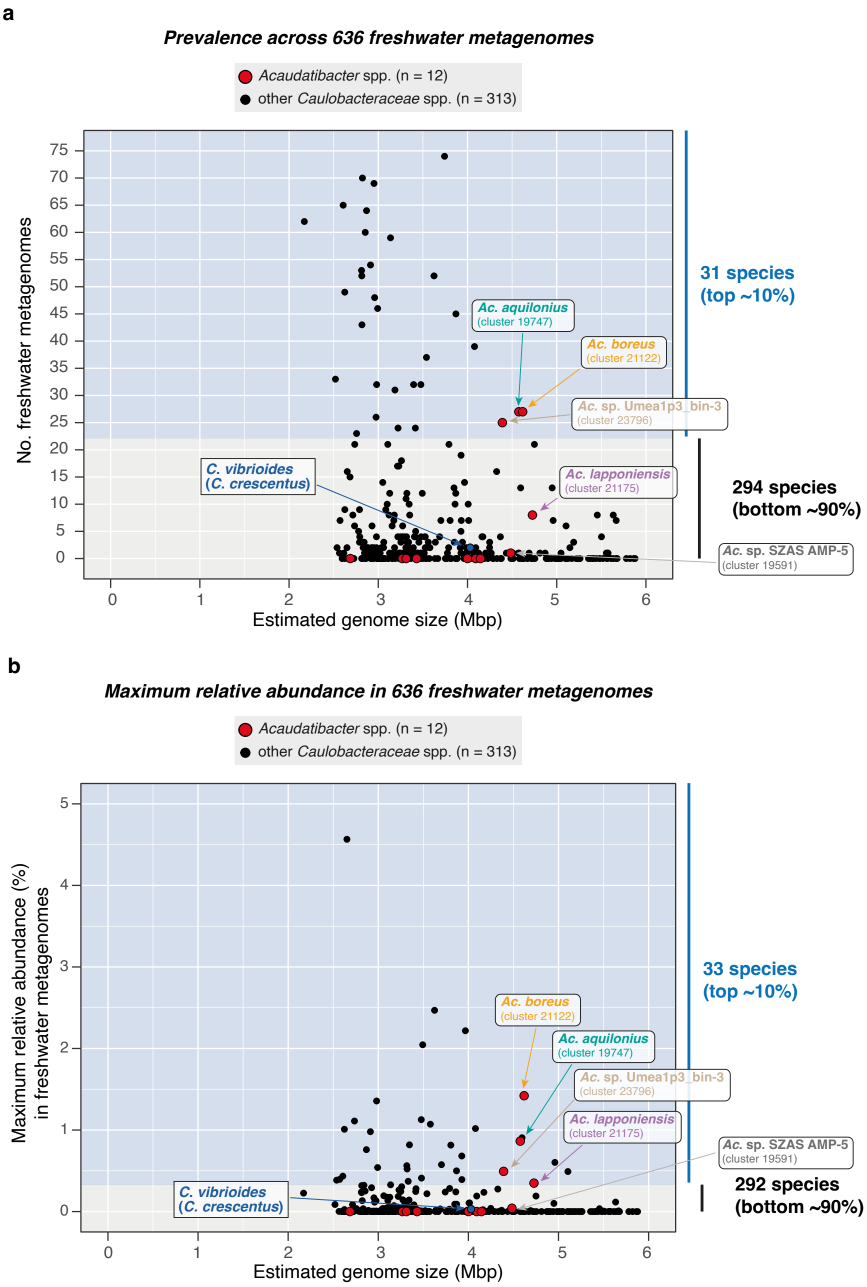

Figure S23 | Putatively photoautotrophic *Acaudatibacter* species are among the most prevalent and relatively abundant *Caulobacteraceae* species in freshwater metagenomes. (a) Prevalence of *Acaudatibacter* species (red circles) and other *Caulobacteraceae* species (black circles) across the 636 freshwater metagenomes from a recent dataset[36]. *C. vibrioides* (synonym. *C. crescentus*) is marked with a blue circle. Source data are provided as a Source Data file. **(b)** Maximum relative abundance of *Acaudatibacter* species (red circles) compared to other *Caulobacteraceae* species (black circles) in the investigated 636 freshwater metagenomes. *C. vibrioides* (synonym. *C. crescentus*) is marked with a blue circle. Source data are provided as a Source Data file.

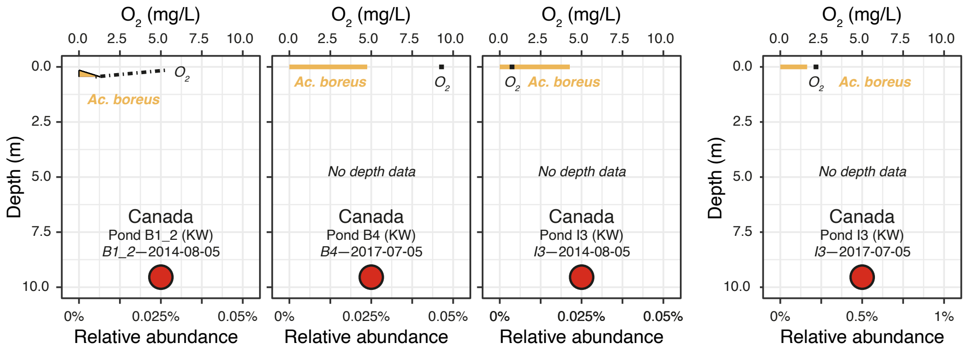

Figure S24. | Relative abundance of *Acaudatibacter* species among metagenomes from Canadian freshwater bodies in which sampling was not done at different depths of the water column. Presented as in **Fig. 6c**; for each sampling series, the name of the freshwater body, geographical region (parentheses), lake code (italics), and sampling date are indicated. Source data are provided as a Source Data file.

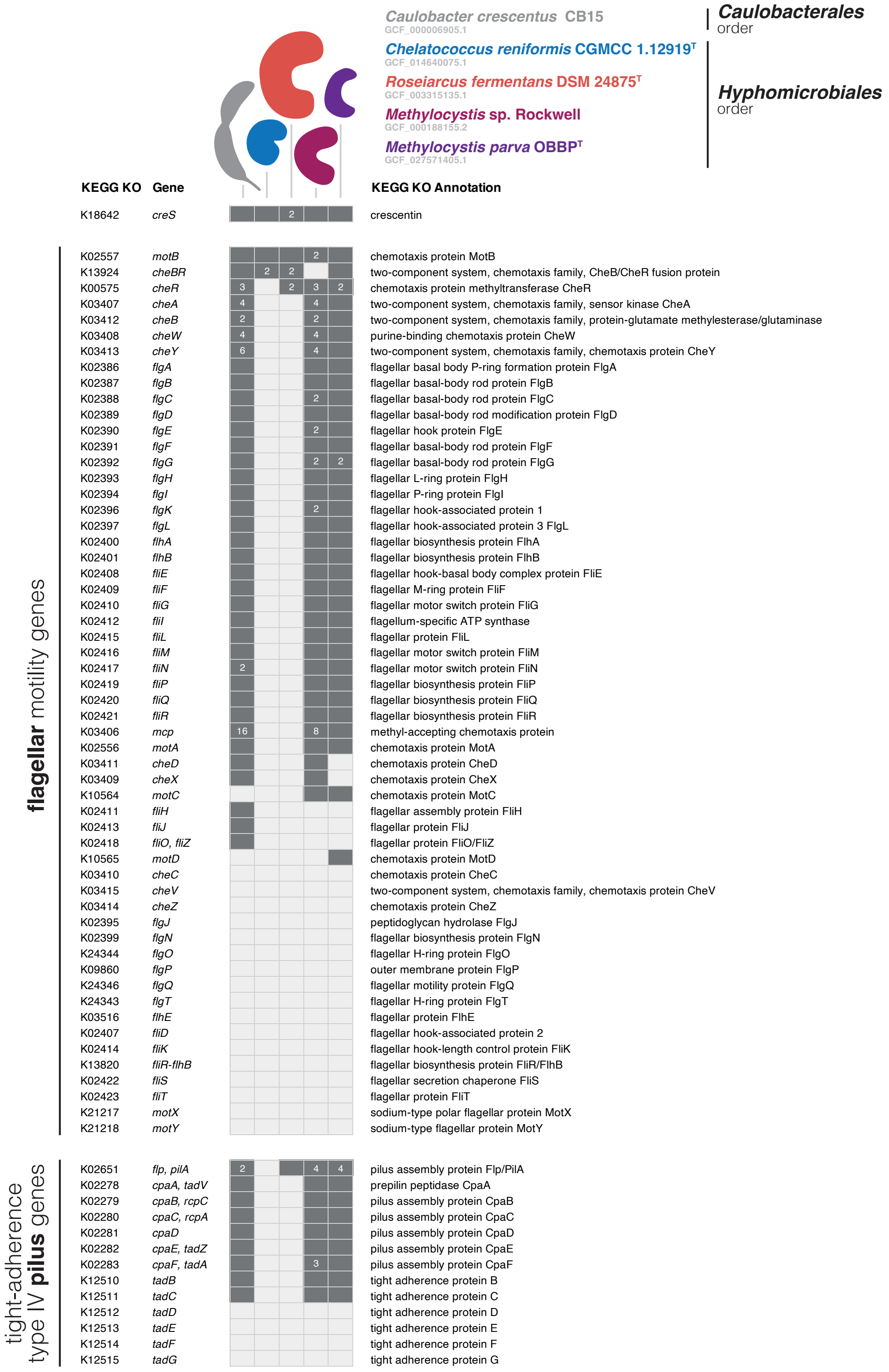

Figure S25 | Two curved crescentin-encoding *Hyphomicrobiales* species lack genes for both flagella and type IV pili. Presence (dark gray) and absence (light gray) of flagellar and type IV tight-adhesion pili (T4P) KEGG orthologs (KOs) predicted using eggNOG-mapper[39] (v2.1.12) among curved *creS*-encoding *Hyphomicrobiales* species. Numbers show KO copy numbers above one.

SUPPLEMENTARY TABLES

Table S1. List of published micrographs used as reference for drawing schematic illustrations in **Figs. 1a** and **4d**.

Species	Micrograph used as guide for drawing		Modifications made to cell outline
	**Figure/file in orig. publ.**	**Reference**	
P. haematophilum	Figure 2a	43	
P. glaciei	Figure 2	44	
P. parvum	Figure S1	45	
C. rhizosphaerae	Figure S1*	46	*Figs. S1a and S1b of Sun *et al.*[46] were combined; prostheca based on Fig. S1b added to predivisional cell outline based on Fig. S1a.
Po. montana comb. nov. (*P. montanum*)	Figure 2	5	
B. vesicularis	Figure 2	47	
B. mediterranea	Figure 2	47	
B. nasdae	Figure 1	47	
B. terrae	Figure 2	47	
B. pondensis	Figure 4a	48	
B. basaltis	Figure 2	47	Prostheca based on non-predivisional cell was added to cell outline based on predivisional cell.
B. denitrificans	Figure 2	49	
B. aveniformis	Figure 1	47	
A. tiandongensis	Figure S1	50	
A. benevestitus	Figure 1b	51	
A. taihuensis	Figure 1	52	
Hyphomonas neptunia	Figure 6c	53	
Hyphomonas adhaerens	Figure 1	54	
Henriciella barbarensis	Figure 2	55	
Ponticaulis koreensis	Figure 2	56	
Hirschia baltica	Figure 1	57	
Vitreimonas silvestris comb. nov. (*Terricaulis silvestris*)	Figure S1B	4	
Vitreimonas flagellata	Figure S1	3	
Pseudaquidulcibacter paucihalophilus	Figure 1b	2	
"Maricaulis alexandrii"	Figure 1d	58	
Hyphomicrobium indicum	Figure 2a	59	
Glycocaulis alkaliphilus	Figure 1a	60	
Marinicauda pacifica	Figure S2a	61	
Oceanicaulis alexandrii	Figure 3a	17	
Oceanicaulis satelles comb. nov. (*Alkalicaulis satelles*)	Figure 1a	6	
Woodsholea maritima	Figure 1a	62	
Robiginitomaculum antarcticum	Figure 2a	63	Prostheca-like protrusion seen on non-predivisional cell was added to cell outline based on predivisional cell.
Parvularcula bermudensis	Figure 1a	64	
Aquisalinus flavus	Figure S1	65	
Amphiplicatus metrithermophilus	Figure S1a	66	
Methylocystis parva	Figure BXII.α.153	67	
Methylocystis sp. Rockwell	Figure 2a	68	
Roseiarcus fermentans	Figure 1a	69	

Table S2. List of strains used in this work. Abbreviations: genta, gentamicin; DSMZ, German Collection of Microorganisms and Cell Cultures; CCUG, Culture Collection University of Gothenburg.

Species	#	Strain	Background	Genotype	Medium	Source	Ref.
Aquidulcibacter paucihalophilus	KJ1143	DSM 109892^{T} [TH1-2^{T}]			PYE	DSMZ	
Asticcacaulis biprosthecius	KJ1167	DSM 4723^{T} [C19^{T}]			PYE	DSMZ	
Asticcacaulis excentricus	KJ1168	DSM 4724^{T} [CB48^{T}]			PYE	DSMZ	
Brevundimonas aurantiaca	KJ1149	CCUG 45020^{T} [CB-R^{T}]			PYE	CCUG	
Brevundimonas bacteroides	KJ998	DSM4726^{T} [CB7^{T}]			PYE	DSMZ	
Brevundimonas diminuta	KJ1144	DSM 7234^{T} [Pickett K-248^{T}]			PYE	DSMZ	
Brevundimonas goettingensis	KJ1145	DSM 112305^{T} [LVF2^{T}]			PYE	DSMZ	
Brevundimonas lenta	KJ1146	DSM 23960^{T} [DS-18^{T}]			PYE	DSMZ	
Brevundimonas subvibrioides	KJ1148	DSM 4735^{T} [CB81^{T}]			PYE	DSMZ	
Brevundimonas variabilis	KJ997	DSM4737^{T} [CB17^{T}]			PYE	DSMZ	
Caulobacter crescentus (*C. vibrioides*)	KJ883	CB15			PYE	S. Crosson	
Caulobacter crescentus (*C. vibrioides*)	KJ1	NA1000			PYE	M. Laub	[70]
Caulobacter crescentus (*C. vibrioides*)	KJ1183		NA1000	pBXMCS-4 (EV)	PYE + genta	This work	
Caulobacter crescentus (*C. vibrioides*)	KJ1184		NA1000	pBXMCS-4-P$_{xyl}$-*creS*	PYE + genta	This work	
Caulobacter crescentus (*C. vibrioides*)	KJ1185		NA1000	pBXMCS-4-P$_{xyl}$-*creS*$_{Ch.reniformis}$	PYE + genta	This work	
Caulobacter crescentus (*C. vibrioides*)	KJ1179	CJW1208 (LS3812)	NA1000	Δ*creS*	PYE	C. Jacobs-Wagner	[71]
Caulobacter crescentus (*C. vibrioides*)	KJ1186		CJW1208	Δ*creS* pBXMCS-4 (EV)	PYE + genta	This work	
Caulobacter crescentus (*C. vibrioides*)	KJ1187		CJW1208	Δ*creS* pBXMCS-4-P$_{xyl}$-*creS*	PYE + genta	This work	
Caulobacter crescentus (*C. vibrioides*)	KJ1188		CJW1208	Δ*creS* pBXMCS-4-P$_{xyl}$-*creS*$_{Ch.reniformis}$	PYE + genta	This work	
Caulobacter flavus	KJ1169	DSM 29968^{T} [RHGG3^{T}]			PYE	DSMZ	
Caulobacter henricii	KJ1151	CCUG 49339^{T} [CB4^{T}]			PYE	CCUG	
Caulobacter mirabilis	KJ1170	DSM 21795^{T} [FWC38^{T}]			PYE	DSMZ	
Caulobacter segnis	KJ1147	DSM 7131^{T} [TK0059^{T}]			PYE	DSMZ	
Caulobacteraceae sp. PMMR1	KJ1174	DSM 26776 [PMMR1]			R2A pH 6.0	DSMZ	
Chelatococcus reniformis	KJ1171	DSM 105737^{T} [B2974^{T}]			PYE	DSMZ	
Escherichia coli	KJ665	MG1655	K12	F^{-} λ^{-} *ilvG*$^{-}$ *rfb*-50 *rph*-1	LB	M. Laub	
Escherichia coli	N/A	DH5α		[general cloning strain]	LB	Invitrogen	
Escherichia coli	N/A		TOP10	pBXMCS-4 (EV)	LB + genta	M. Thanbichler	[72]
Escherichia coli	KJ1181		DH5α	pBXMCS-4-P$_{xyl}$-*creS*	LB + genta	This work	
Escherichia coli	KJ1182		DH5α	pBXMCS-4-P$_{xyl}$-*creS*$_{Ch.reniformis}$	LB + genta	This work	
Phenylobacterium deserti	KJ1172	DSM 103871^{T} [YIM 73061^{T}]			R2A	DSMZ	
Phenylobacterium immobile	KJ1173	DSM 1986^{T} [E^{T}]			R2A	DSMZ	

Table S3. List of oligonucleotides used in this work.

Name	Sequence (5′–3′)
OJH25	atggtcgtctccccaaaactc
OJH26	ctgcagcccgggggatccactag
OJH27	gctcgagttttggggagacgaccatatgagactgctgtcgaaga
OJH28	aactagtggatcccccgggctgcagttaggcgctcgcggccacg
OJH29	gctcgagttttggggagacgaccatatgatcggtattggacagc
OJH30	aactagtggatcccccgggctgcagttattcggccgcggtattg

SUPPLEMENTARY MOVIE LEGENDS

Supplementary Movie 1. Timelapse microscopy of *P. immobile* E^T at 30°C on R2A 1% agarose, in 5-minute imaging intervals. Playback speed: 18 frames/s (1.5 h/s). Scale bars: 1 µm. File format: .mp4. **(a)** The cell shown in **Fig**. **3a**. **(b)** Three additional cells.

Supplementary Movie 2. Timelapse microscopy of *E. coli* MG1655 at 37°C on LB 1% agarose, in 20-second imaging intervals. Playback speed: 30 frames/s (15 min/s). Scale bars: 1 µm. File format: .mp4. **(a)** The cell shown in **Fig**. **3a**. **(b)** Three additional cells.

Supplementary Movie 3. Timelapse microscopy of *C. crescentus* CB15 at 30°C on PYE 1% agarose, in 30-second imaging intervals. Playback speed: 30 frames/s (15 min/s). Scale bars: 1 µm. File format: .mp4. **(a)** The cell shown in **Fig**. **3a**. **(b)** Six additional cells.

SUPPLEMENTARY DATA LEGENDS

Supplementary Data 1 | Genome overview. Overview of genomes used in this work. **(a)** The "core dataset" of *Caulobacterales* genomes. Columns A–C, genome name, accession, and taxon name; Columns D–G, information on genome dereplication and selection of species representatives; Column H, GTDB taxonomy (release R207); Columns I–R, genome statistics, including assembly size, estimated genome size, N50, number of contigs, G+C content, and estimates of completeness and contamination from both the CheckM[33] methods 'taxonomy_wf' (used for genome selection) and 'lineage_wf' (used for estimated genome size calculation); Columns S–U, additional information. **(b)** The "extended dataset" of *Acaudatibacter* gen. nov. (GTDB taxon "g__Palsa-881") genomes sourced from Rodríguez-Gijón *et al.*[36] Column A, genome name; Column B, references for assemblies[36-38, 73-76]; Column C–D, species clustering information; Column E, whether the genome is also included as a species genome representative (SGR) in the "core dataset"; Column F, type of genome (MAG, metagenome-assembled genome); Column G, GTDB taxonomy (release R207); Columns H–N, genome statistics, including assembly size, estimated genome size, number of contigs, number of scaffolds, G+C content, and estimates of completeness and contamination from the CheckM method 'lineage_wf'.

Supplementary Data 2 | Environmental metadata. Meta analysis of genome sampling environment metadata. **(a)** Explanation of the table layout and content of the metadata. **(b)** Manually collected and curated metadata from NCBI BioSample (www.ncbi.nlm.nih.gov/biosample/) and BioProject (www.ncbi.nlm.nih.gov/bioproject/) pages, as well as JGI Gold (gold.jgi.doe.gov/), and listed publications when necessary. Listed literature references: [2, 6, 7, 9-12, 17, 21, 22, 28, 43, 46, 52, 54, 55, 58, 62, 64-66, 77-118].

Supplementary Data 3 | IMNGS environmental data. Compiled IMNGS[32] 'Taxonomy' job results for the query: "Bacteria/Proteobacteria/Alphaproteobacteria/Caulobacterales/Caulobacteraceae".

Supplementary Data 4 | Selected gene presence/absence data. Gene presence and absence data presented in figures and supplementary figures of the article (**Figs. 2b**, **4b**, and **5b**, **Supplementary Figs. S6**, **S8**, and **S16**, and **Supplementary Data 5** and **8**). Includes genes for chemotaxis, flagellar motility, cell cycle and development, type IV pilus, holdfast synthesis, crescentin, S-layer, prostheca, cell division, carotenoid synthesis, photosynthesis, carbon fixation, aerobic respiration, secretion systems, and sulfonate transport. Includes both *Caulobacterales* genomes of the "core dataset", and *Acaudatibacter* genomes of the "extended dataset". For gene orthologs, attributes are separated by "@" in the following order: (1) gene category, (2) annotation tool, (3) KEGG KO or *C. crescentus* locus IDs, (4) gene name, (5) EC number, (6) gene annotation, (7) manually curated gene name. For RBH results, the attributes #4 and #7 were taken from the *C. crescentus* NA1000 GCF_000022005.1 assembly, since it is better annotated, but they all agree well with the *C. crescentus* CB15 GCF_000006905.1 assembly. For genomes, attributes are separated by "@" in the following order: (1) dataset [either the "core" dataset of *Caulobacterales* species genome representatives or the "extended" dataset of additional *Acaudatibacter*/"Palsa-881" genomes], (2) assembly ID, (3) family, (4) genus, (5) species, (6) taxon name for the genome assembly [only for the "extended" dataset]. KOs of the "core" dataset were annotated using eggNOG-mapper[39] (emapper) v2.1.5 or GhostKOALA[42] v2.3 and KOs of the "extended" dataset were annotated using eggNOG-mapper (emapper) v2.1.12 or GhostKOALA v3.0.

Supplementary Data 5 | Overview of the presence and absence of cell morphology and development genes across *Caulobacterales*. **(a)** Species phylogeny shown in **Fig. 1a**. Numbers

represent non-parametric bootstraps and the scale bar indicates number of substitutions per site. (**b–j**) Expanded view of the presence and absence of genes presented in **Fig. 2b**, showing genes involved in (b) chemotaxis, (c) flagellum, (d) cell cycle and developmental genes, (e) type IV adhesive pilus (T4P), (f) holdfast, (g) crescentin, (h) S-layer, (i) prostheca, and (j) cell division, among *Caulobacterales* genomes. Gene orthologs were identified using KEGG ortholog (KO) annotations from eggNOG-mapper[39] v2.1.5 (dark gray) or through the reciprocal best blast hit (RBH) algorithm using the *C. crescentus* CB15 proteome (blue). For RBH results, the corresponding loci in the *C. crescentus* CB15 (CC numbers) and *C. crescentus* NA1000 (CCNA numbers) are shown alongside the gene name. Descriptions come from the KO annotation or from the *C. crescentus* NA1000 genome annotation. Numbers show KO copy numbers > 1. The full dataset is found in **Supplementary Data 4**.

Supplementary Data 6 | Uncharacterized putative flagellar/developmental genes. Identification of putative flagellar motility and development factors, based on their absence from non-flagellated *Acaudatibacter* gen. nov. and *Phenylobacterium* species. Presence/absence is based on the reciprocal best blast hit (RBH) algorithm. The 100 genes missing from non-flagellated lineages, sorted by their conservation in the *Acaudatibacter–Caulobacter–Phenylobacterium* (ACP) clade. For gene orthologs, attributes are separated by "@" in the following order: (1) gene category, (2) *C. crescentus* CB15 protein accession, (3) CB15 new locus ID, (4) CB15 old locus ID, (5) CB15 gene name, (6) CB15 gene annotation, (7) *C. crescentus* NA1000 protein accession, (8) NA1000 locus ID, (9) NA1000 gene name, (10) NA1000 gene annotation, (11) total number of species genome representatives having the gene within the ACP clade [basis for sorting]. For genomes, attributes are separated by "@" as presented in **Supplementary Data 4**.

Supplementary Data 7 | Colony pigments. Meta analysis of the colony pigment description terms used in the literature for *Caulobacterales* isolates included in our dataset, if available. Listed literature references: [1-4, 6, 7, 9, 17, 21, 22, 28, 29, 43-46, 48-52, 54-63, 65, 66, 79, 80, 83, 85, 87, 90-92, 94-99, 101, 102, 108, 109, 111-113, 115, 117, 119-145].

Supplementary Data 8 | Overview of the presence and absence of phototrophy and respiration genes across *Caulobacterales*. (a) Species phylogeny shown in **Fig. 1a**. Numbers represent non-parametric bootstraps and the scale bar indicates number of substitutions per site. Genomes containing phototrophy genes are marked with red circles. **(b)** Meta-analysis of colony pigments across *Caulobacterales* species. See **Supplementary Data 7** for colony descriptor words and references. Asterisks: *Caulobacter* isolates ErkDOM-C and ErkDOME pigment descriptions derive from this work. **(c–i)** Expanded view of the presence and absence of genes presented in **Fig. 5b**, showing genes involved in (c) carotenoid biosynthesis, (d) bacteriochlorophyll biosynthesis, (e) bacteriochlorophyll transport, (f) light-harvesting complex II (LH2), (g) reaction center–light-harvesting complex I (RC–LH1), (h) CO_2 fixation using the CBB cycle, and (i) aerobic respiration among *Caulobacterales* genomes. KEGG ortholog (KO) gene ortholog were annotated using either eggNOG-mapper[39] (emapper; v2.1.5) or GhostKOALA[42] (v2.2). Numbers show KO copy numbers > 1. Abbreviations: cytochrome (cyt.). Full dataset is found in **Supplementary Data 4**. **(j)** Schematic representation of the highly branched electron transport chain of *C. crescentus* CB15, which includes two high-affinity terminal oxidases operating under low-oxygen concentrations (cytochromes *bd* and bb_3) and two low-affinity terminal oxidases operating under high-oxygen concentrations (cytochromes bo_3 and aa_3)[146].

Supplementary Data 9 | Overview of the presence and absence of carbon fixation pathways across *Caulobacterales*. Estimated completeness of carbon fixation pathways among *Caulobacterales*

genomes based on KEGG ortholog (KO) annotations from eggNOG-mapper[39] (v2.1.5) and associated KEGG modules. **(a)** Species phylogeny shown in **Fig. 1a**. Numbers represent non-parametric bootstraps and the scale bar indicates number of substitutions per site. Genomes containing phototrophic potential are marked with red circles for *Caulobacterales* and with dark blue circles for outgroup *Alphaproteobacteria*. Genomes with complete genetic potential for the CBB cycle are marked with orange circles. **(b)** Calvin-Benson-Bassham (CBB) cycle—KEGG module M00165. *Left panel*: Completeness of the CBB cycle steps. *Right panel*: Copy number of individual CBB cycle KOs. **(c)** Completeness of the reductive citrate cycle steps—KEGG module M00173. **(d)** Completeness of the 3-hydroxy-propionate bicycle steps—KEGG module M00376.

Supplementary Data 10 | Overview of eggNOG-mapper-predicted NOGs. Overview of eggNOG-mapper[39] (emapper) annotations of non-supervised orthologous groups (NOGs) in *Caulobacterales* species genome representatives of the "core dataset" (emapper v2.1.5). For each genome, attributes are separated by "@" as presented in **Supplementary Data 4**.

Supplementary Data 11 | Overview of eggNOG-mapper-predicted KOs. Overview of eggNOG-mapper[39] (emapper) KEGG ortholog (KO) annotations of **(a)** *Caulobacterales* species genome representatives of the "core dataset" (emapper v2.1.5), **(b)** *Acaudatibacter* gen. nov. ("Palsa-881") genomes of the "extended dataset" (emapper v2.1.12), or **(c)** genomes of curved crescentin-encoding *Hyphomicrobiales* species (emapper v2.1.12). For each genome, attributes are separated by "@" as presented in **Supplementary Data 4**.

Supplementary Data 12 | Overview of GhostKOALA-predicted KOs. Overview of GhostKOALA[42] v2.2 KEGG ortholog (KO) annotations of **(a)** *Caulobacterales* species genome representatives of the "core dataset" (GhostKOALA v2.3), or **(b)** *Acaudatibacter* gen. nov. ("Palsa-881") genomes of the "extended dataset" (GhostKOALA v3.0). For each genome, attributes are separated by "@" as presented in **Supplementary Data 4**.

Supplementary Data 13 | KEGG Decoder: pathway completeness. Pathway completeness prediction using KEGG Decoder[147] v1.3 with GhostKOALA-predicted KOs listed in **Supplementary Data 12**.

Supplementary Data 14 | Overview of RBHs. Overview of reciprocal best blast hit (RBH) results for the *C. crescentus* CB15 proteome of the GCF_000006905.1 assembly queried against **(a)** *Caulobacterales* species genome representatives of the "core dataset", and **(b)** *Acaudatibacter* gen. nov. ("Palsa-881") genomes of the "extended dataset". For each genome, attributes are separated by "@" as presented in **Supplementary Data 4**.

Supplementary Data 15 | Pairwise ANI. Overview of pairwise average nucleotide identity (ANI) comparisons using FastANI[148] v1.33 for *Caulobacterales* genomes of the "core dataset" as well as *Acaudatibacter* gen. nov. ("Palsa-881") species of the "extended dataset". For each genome, attributes are separated by "@" as presented in **Supplementary Data 4**. Note that FastANI simply outputs "NA" for ANIs far below 80%.

Supplementary Data 16 | Pairwise AAI. Overview of pairwise average amino acid identity (AAI) comparisons using EzAAI[149] v1.2.3 for *Caulobacterales* genomes of the "core dataset" as well as *Acaudatibacter* gen. nov. ("Palsa-881") species of the "extended dataset". For each genome, attributes are separated by "@" as presented in **Supplementary Data 4**.

Supplementary Data 17 | Manual refinement of species phylogeny. Sequences manually removed when making the manually refined ML species phylogeny presented in **Fig. 1a** and **Supplementary Fig. S1**, which included the removal of putative paralogs, contamination, long-branching, horizontal transfers, and duplicate sequences.

Supplementary Data 18 | 16S and 23S rRNA gene hits from Barrnap. Overview of the longest 16S and 23S rRNA gene sequences retrieved from *Caulobacterales* species representative genomes using Barrnap v0.9 (https://github.com/tseemann/barrnap), with a cutoff of 50% of each gene ("--reject 0.5"), and using bacterial rRNA gene models ("--kingdom bac"), and which were then used to infer the species phylogeny presented in **Supplementary Fig. S3**. "NA" means that no sequence was retrieved.

REFERENCES FOR SUPPLEMENTARY INFORMATION

1. Cai H, Shi Y, Wang Y, Cui H, Jiang H. *Aquidulcibacter paucihalophilus* gen. nov., sp. nov., a novel member of family *Caulobacteraceae* isolated from cyanobacterial aggregates in a eutrophic lake. *Antonie van Leeuwenhoek* **110**, 1169-1177 (2017).

2. Liu Y*, et al. Pseudaquidulcibacter saccharophilus* gen. nov., sp. nov., a novel member of family *Caulobacteraceae*, isolated from a water purification facility with supplement of starch as a carbon source. *International Journal of Systematic and Evolutionary Microbiology* **72**, (2022).

3. Asem MD*, et al. Vitreimonas flagellata* gen. nov., sp. nov., a novel member of the family *Hyphomonadaceae* isolated from an activated sludge sample. *International Journal of Systematic and Evolutionary Microbiology* **70**, 2632-2639 (2020).

4. Vieira S*, et al. Terricaulis silvestris* gen. nov., sp. nov., a novel prosthecate, budding member of the family *Caulobacteraceae* isolated from forest soil. *International Journal of Systematic and Evolutionary Microbiology* **70**, 4966-4977 (2020).

5. Tang Y*, et al. Phenylobacterium montanum* sp. nov., an oligotrophic, slightly acidophilic mesophile isolated from sandy soil. *International Journal of Systematic and Evolutionary Microbiology* **74**, (2024).

6. Kevbrin V, Boltyanskaya Y, Koziaeva V, Uzun M, Grouzdev D. *Alkalicaulis satelles* gen. nov., sp. nov., a novel haloalkaliphile isolated from a laboratory culture cyanobacterium *Geitlerinema* species and proposals of *Maricaulaceae* fam. nov., *Robiginitomaculaceae* fam. nov., *Maricaulales* ord. nov. and *Hyphomonadales* ord. nov. *International Journal of Systematic and Evolutionary Microbiology* **71**, (2021).

7. Leifson E. *Hyphomicrobium neptunium* sp. n. *Antonie van Leeuwenhoek* **30**, 249-256 (1964).

8. Moore RL, Weiner RM, Gebers R. Notes: Genus *Hyphomonas* Pongratz 1957 nom. rev. emend., *Hyphomonas polymorpha* Pongratz 1957 nom. rev. emend., and *Hyphomonas neptunium* (Leifson 1964) comb. nov. emend. (*Hyphomicrobium neptunium*). *International Journal of Systematic Bacteriology* **34**, 71-73 (1984).

9. Weiner RM, Devine RA, Powell DM, Dagasan L, Moore RL. *Hyphomonas oceanitis* sp. nov., *Hyphomonas hirschiana* sp. nov., and *Hyphomonas jannaschiana* sp. nov. *International Journal of Systematic Bacteriology* **35**, 237-243 (1985).

10. Tanabe Y*, et al.* A novel alphaproteobacterial ectosymbiont promotes the growth of the hydrocarbon-rich green alga *Botryococcus braunii*. *Scientific Reports* **5**, 10467 (2015).

11. Tanabe Y, Yamaguchi H, Watanabe MM. Draft Genome Sequence of "*Candidatus* Phycosocius bacilliformis," an Alphaproteobacterial Ectosymbiont of the Hydrocarbon-Producing Green Alga *Botryococcus braunii*. *Genome Announcements* **6**, e00396-00318 (2018).

12. Braun B, Szewzyk U. Complete Genome Sequence of "*Candidatus* Viadribacter manganicus" Isolated from a German Floodplain Area. *Genome Announcements* **4**, (2016).

13. Parks DH, *et al.* A standardized bacterial taxonomy based on genome phylogeny substantially revises the tree of life. *Nature Biotechnology* **36**, 996-1004 (2018).

14. Goris J, Konstantinidis KT, Klappenbach JA, Coenye T, Vandamme P, Tiedje JM. DNA–DNA hybridization values and their relationship to whole-genome sequence similarities. *International Journal of Systematic and Evolutionary Microbiology* **57**, 81-91 (2007).

15. Konstantinidis KT, Tiedje JM. Genomic insights that advance the species definition for prokaryotes. *Proceedings of the National Academy of Sciences* **102**, 2567-2572 (2005).

16. Konstantinidis KT, Rosselló-Móra R, Amann R. Uncultivated microbes in need of their own taxonomy. *The ISME Journal* **11**, 2399-2406 (2017).

17. Strömpl C, *et al. Oceanicaulis alexandrii* gen. nov., sp. nov., a novel stalked bacterium isolated from a culture of the dinoflagellate *Alexandrium tamarense* (Lebour) Balech. *International Journal of Systematic and Evolutionary Microbiology* **53**, 1901-1906 (2003).

18. Qin Q-L, *et al.* A Proposed Genus Boundary for the Prokaryotes Based on Genomic Insights. *Journal of Bacteriology* **196**, 2210-2215 (2014).

19. Kuzyk SB, Jafri M, Humphrey E, Maltman C, Kyndt JA, Yurkov V. Prosthecate aerobic anoxygenic phototrophs *Photocaulis sulfatitolerans* gen. nov. sp. nov. and *Photocaulis rubescens* sp. nov. isolated from alpine meromictic lakes in British Columbia, Canada. *Archives of Microbiology* **204**, (2022).

20. Madeira F, *et al.* The EMBL-EBI Job Dispatcher sequence analysis tools framework in 2024. *Nucleic Acids Research* **52**, W521-W525 (2024).

21. Jung JY, Kim JM, Jin HM, Kim SY, Park W, Jeon CO. *Litorimonas taeanensis* gen. nov., sp. nov., isolated from a sandy beach. *International Journal of Systematic and Evolutionary Microbiology* **61**, 1534-1538 (2011).

22. Liu C, *et al. Algimonas arctica* sp. nov., isolated from intertidal sand, and emended description of the genus *Algimonas*. *International Journal of Systematic and Evolutionary Microbiology* **65**, 3256-3261 (2015).

23. Tanabe Y, Yamaguchi H, Yoshida M, Kai A, Okazaki Y. Characterization of a bloom-associated alphaproteobacterial lineage, '*Candidatus* Phycosocius': insights into freshwater algal-bacterial interactions. *ISME Communications* **3**, (2023).

24. Lee MD. GToTree: a user-friendly workflow for phylogenomics. *Bioinformatics* **35**, 4162-4164 (2019).

25. Martijn J, Vosseberg J, Guy L, Offre P, Ettema TJG. Deep mitochondrial origin outside the sampled alphaproteobacteria. *Nature* **557**, 101-105 (2018).

26. Lau CKY, Krewulak KD, Vogel HJ. Bacterial ferrous iron transport: the Feo system. *FEMS Microbiology Reviews* **40**, 273-298 (2016).

27. Van Gemerden H, Mas J. Ecology of Phototrophic Sulfur Bacteria. In: *Anoxygenic Photosynthetic Bacteria* (eds Blankenship RE, Madigan MT, Bauer CE). Springer Netherlands (1995).

28. Poindexter JS. Biological properties and classification of the *Caulobacter* group. *Bacteriol Rev* **28**, 231-295 (1964).

29. Patel S, Fletcher B, Scott DC, Ely B. Genome Sequence and Phenotypic Characterization of *Caulobacter segnis*. *Current Microbiology* **70**, 355-363 (2015).

30. Nguyen L-T, Schmidt HA, von Haeseler A, Minh BQ. IQ-TREE: A Fast and Effective Stochastic Algorithm for Estimating Maximum-Likelihood Phylogenies. *Molecular Biology and Evolution* **32**, 268-274 (2014).

31. Parks DH, Chuvochina M, Rinke C, Mussig AJ, Chaumeil P-A, Hugenholtz P. GTDB: an ongoing census of bacterial and archaeal diversity through a phylogenetically consistent, rank normalized and complete genome-based taxonomy. *Nucleic Acids Research* **50**, D785-D794 (2021).

32. Lagkouvardos I*, et al.* IMNGS: A comprehensive open resource of processed 16S rRNA microbial profiles for ecology and diversity studies. *Scientific Reports* **6**, 33721 (2016).

33. Parks DH, Imelfort M, Skennerton CT, Hugenholtz P, Tyson GW. CheckM: assessing the quality of microbial genomes recovered from isolates, single cells, and metagenomes. *Genome Research* **25**, 1043-1055 (2015).

34. Bernal P, Llamas MA, Filloux A. Type VI secretion systems in plant-associated bacteria. *Environmental Microbiology* **20**, 1-15 (2018).

35. Deng W*, et al.* Assembly, structure, function and regulation of type III secretion systems. *Nature Reviews Microbiology* **15**, 323-337 (2017).

36. Rodríguez-Gijón A*, et al.* The ecological success of freshwater microorganisms is mediated by streamlining and biotic interactions. Preprint at https://doi.org/10.1101/2025.03.24.644981 (2025).

37. Nayfach S*, et al.* A genomic catalog of Earth's microbiomes. *Nature Biotechnology* **39**, 499-509 (2021).

38. Buck M*, et al.* Comprehensive dataset of shotgun metagenomes from oxygen stratified freshwater lakes and ponds. *Scientific Data* **8**, (2021).

39. Cantalapiedra CP, Hernández-Plaza A, Letunic I, Bork P, Huerta-Cepas J. eggNOG-mapper v2: Functional Annotation, Orthology Assignments, and Domain Prediction at the Metagenomic Scale. *Molecular Biology and Evolution* **38**, 5825-5829 (2021).

40. Jones P*, et al.* InterProScan 5: genome-scale protein function classification. *Bioinformatics* **30**, 1236-1240 (2014).

41. Römling U, Liang Z-X, Dow JM. Progress in Understanding the Molecular Basis Underlying Functional Diversification of Cyclic Dinucleotide Turnover Proteins. *Journal of Bacteriology* **199**, e00790-00716 (2017).

42. Kanehisa M, Sato Y, Morishima K. BlastKOALA and GhostKOALA: KEGG Tools for Functional Characterization of Genome and Metagenome Sequences. *Journal of Molecular Biology* **428**, 726-731 (2016).

43. Abraham WR*, et al.* Phylogeny by a polyphasic approach of the order *Caulobacterales*, proposal of *Caulobacter mirabilis* sp. nov., *Phenylobacterium haematophilum* sp. nov. and *Phenylobacterium conjunctum* sp. nov., and emendation of the genus *Phenylobacterium*. *International Journal of Systematic and Evolutionary Microbiology* **58**, 1939-1949 (2008).

44. Thomas FA, Sinha RK, Hatha AAM, Krishnan KP. *Phenylobacterium glaciei* sp. nov., isolated from Vestrebroggerbreen, a valley glacier in Svalbard, Arctic. *International Journal of Systematic and Evolutionary Microbiology* **72**, (2022).

45. Baek C, Shin S-K, Yi H. *Phenylobacterium parvum* sp. nov., isolated from lake water. *International Journal of Systematic and Evolutionary Microbiology* **69**, 1169-1172 (2019).

46. Sun L-N, Yang E-D, Hou X-T, Wei J-C, Yuan Z-X, Wang W-Y. *Caulobacter rhizosphaerae* sp. nov., a stalked bacterium isolated from rhizosphere soil. *International Journal of Systematic and Evolutionary Microbiology* **67**, 1771-1776 (2017).

47. Curtis PD. Stalk formation of *Brevundimonas* and how it compares to *Caulobacter crescentus*. *PLOS ONE* **12**, e0184063 (2017).

48. Friedrich I, Klassen A, Neubauer H, Schneider D, Hertel R, Daniel R. Living in a Puddle of Mud: Isolation and Characterization of Two Novel *Caulobacteraceae* Strains *Brevundimonas pondensis* sp. nov. and *Brevundimonas goettingensis* sp. nov. *Applied Microbiology* **1**, 38-59 (2021).

49. Tsubouchi T*, et al. Brevundimonas denitrificans* sp. nov., a denitrifying bacterium isolated from deep subseafloor sediment. *International Journal of Systematic and Evolutionary Microbiology* **64**, 3709-3716 (2014).

50. Zhou X-K*, et al. Asticcacaulis tiandongensis* sp. nov., a new member of the genus *Asticcacaulis*, isolated from a cave soil sample. *International Journal of Systematic and Evolutionary Microbiology* **70**, 687-692 (2020).

51. Vasilyeva LV*, et al. Asticcacaulis benevestitus* sp. nov., a psychrotolerant, dimorphic, prosthecate bacterium from tundra wetland soil. *International Journal of Systematic and Evolutionary Microbiology* **56**, 2083-2088 (2006).

52. Liu Z-P, Wang B-J, Liu S-J, Liu Y-H. *Asticcacaulis taihuensis* sp. nov., a novel stalked bacterium isolated from Taihu Lake, China. *International Journal of Systematic and Evolutionary Microbiology* **55**, 1239-1242 (2005).

53. Jung A*, et al.* Two-step chromosome segregation in the stalked budding bacterium *Hyphomonas neptunium*. *Nature Communications* **10**, (2019).

54. Weiner RM, Melick M, O'Neill K, Quintero E. *Hyphomonas adhaerens* sp. nov., *Hyphomonas johnsonii* sp. nov. and *Hyphomonas rosenbergii* sp. nov., marine budding

and prosthecate bacteria. *International Journal of Systematic and Evolutionary Microbiology* **50**, 459-469 (2000).

55. Abraham W-R*, et al.* Proposal of *Henriciella barbarensis* sp. nov. and *Henriciella algicola* sp. nov., stalked species of the genus and emendation of the genus *Henriciella*. *International Journal of Systematic and Evolutionary Microbiology* **67**, 2804-2810 (2017).

56. Kang HS, Lee SD. *Ponticaulis koreensis* gen. nov., sp. nov., a new member of the family *Hyphomonadaceae* isolated from seawater. *International Journal of Systematic and Evolutionary Microbiology* **59**, 2951-2955 (2009).

57. Schlesner H, Bartels C, Sittig M, Dorsch M, Stackebrandt E. Taxonomic and Phylogenetic Studies on a New Taxon of Budding, Hyphal *Proteobacteria*, *Hirschia baltica* gen. nov., sp. nov. *International Journal of Systematic Bacteriology* **40**, 443-451 (1990).

58. Zhang X-L, Qi M, Li Q-H, Cui Z-D, Yang Q. *Maricaulis alexandrii* sp. nov., a novel active bioflocculants-bearing and dimorphic prosthecate bacterium isolated from marine phycosphere. *Antonie van Leeuwenhoek* **114**, 1195-1203 (2021).

59. Ruan C-J*, et al. Hyphobacterium indicum* sp. nov., isolated from deep seawater, and emended description of the genus *Hyphobacterium*. *International Journal of Systematic and Evolutionary Microbiology* **68**, 3760-3765 (2018).

60. Geng S*, et al. Glycocaulis alkaliphilus* sp. nov., a dimorphic prosthecate bacterium isolated from crude oil. *International Journal of Systematic and Evolutionary Microbiology* **65**, 838-844 (2015).

61. Zhang X-Y*, et al. Marinicauda pacifica* gen. nov., sp. nov., a prosthecate alphaproteobacterium of the family *Hyphomonadaceae* isolated from deep seawater. *International Journal of Systematic and Evolutionary Microbiology* **63**, 2248-2253 (2013).

62. Abraham W-R*, et al. Woodsholea maritima* gen. nov., sp. nov., a marine bacterium with a low diversity of polar lipids. *International Journal of Systematic and Evolutionary Microbiology* **54**, 1227-1234 (2004).

63. Lee K, Lee HK, Choi T-H, Cho J-C. *Robiginitomaculum antarcticum* gen. nov., sp. nov., a member of the family *Hyphomonadaceae*, from Antarctic seawater. *International Journal of Systematic and Evolutionary Microbiology* **57**, 2595-2599 (2007).

64. Cho J-C, Giovannoni SJ. *Parvularcula bermudensis* gen. nov., sp. nov., a marine bacterium that forms a deep branch in the *α-Proteobacteria*. *International Journal of Systematic and Evolutionary Microbiology* **53**, 1031-1036 (2003).

65. Zhong Z-P, Liu Y, Wang F, Zhou Y-G, Liu H-C, Liu Z-P. *Aquisalinus flavus* gen. nov., sp. nov., a member of the family *Parvularculaceae* isolated from a saline lake. *International Journal of Systematic and Evolutionary Microbiology* **66**, 1813-1817 (2016).

66. Zhen-Li Z*, et al.* *Amphiplicatus metriothermophilus* gen. nov., sp. nov., a thermotolerant alphaproteobacterium isolated from a hot spring. *International Journal of Systematic and Evolutionary Microbiology* **64**, 2805-2811 (2014).

67. Bowman JP. Family V. *Methylocystaceae*. In: *Bergey's Manual® of Systematic Bacteriology* (eds Brenner DJ, Krieg NR, Staley JT). 2 edn. Springer New York, NY (2005).

68. Lazic M, Gudneppanavar R, Whiddon K, Sauvageau D, Stein LY, Konopka M. In vivo quantification of polyhydroxybutyrate (PHB) in the alphaproteobacterial methanotroph, *Methylocystis* sp. Rockwell. *Applied Microbiology and Biotechnology* **106**, 811-819 (2022).

69. Kulichevskaya IS, Danilova OV, Tereshina VM, Kevbrin VV, Dedysh SN. Descriptions of *Roseiarcus fermentans* gen. nov., sp. nov., a bacteriochlorophyll *a*-containing fermentative bacterium related phylogenetically to alphaproteobacterial methanotrophs, and of the family *Roseiarcaceae* fam. nov. *International Journal of Systematic and Evolutionary Microbiology* **64**, 2558-2565 (2014).

70. Evinger M, Agabian N. Envelope-associated nucleoid from *Caulobacter crescentus* stalked and swarmer cells. *Journal of Bacteriology* **132**, 294-301 (1977).

71. Gitai Z, Dye N, Shapiro L. An actin-like gene can determine cell polarity in bacteria. *Proceedings of the National Academy of Sciences* **101**, 8643-8648 (2004).

72. Thanbichler M, Iniesta AA, Shapiro L. A comprehensive set of plasmids for vanillate- and xylose-inducible gene expression in *Caulobacter crescentus*. *Nucleic Acids Research* **35**, e137-e137 (2007).

73. Woodcroft BJ*, et al.* Genome-centric view of carbon processing in thawing permafrost. *Nature* **560**, 49-54 (2018).

74. Ortiz M*, et al.* Multiple energy sources and metabolic strategies sustain microbial diversity in Antarctic desert soils. *Proceedings of the National Academy of Sciences* **118**, e2025322118 (2021).

75. Camargo AP*, et al.* Plant microbiomes harbor potential to promote nutrient turnover in impoverished substrates of a Brazilian biodiversity hotspot. *The ISME Journal* **17**, 354-370 (2023).

76. Wang Y, Zhao R, Liu L, Li B, Zhang T. Selective enrichment of comammox from activated sludge using antibiotics. *Water Research* **197**, 117087 (2021).

77. Anantharaman K*, et al.* Thousands of microbial genomes shed light on interconnected biogeochemical processes in an aquifer system. *Nature Communications* **7**, 13219 (2016).

78. Garcia SL*, et al.* Taxonomic and functional diversity of aquatic heterotrophs is sustained by dissolved organic matter chemodiversity. Preprint at https://doi.org/10.1101/2022.03.21.485019 (2022).

79. Zhang K, Han W, Zhang R, Xu X, Pan Q, Hu X. *Phenylobacterium zucineum* sp. nov., a facultative intracellular bacterium isolated from a human erythroleukemia cell line K562. *Systematic and Applied Microbiology* **30**, 207-212 (2007).

80. Khan IU*, et al. Phenylobacterium deserti* sp. nov., isolated from desert soil. *International Journal of Systematic and Evolutionary Microbiology* **67**, 4722-4727 (2017).

81. Morrison KD*, et al.* Influence of Uranium Concentration and pH on U-Phosphate Biomineralization by *Caulobacter* OR37. *Environmental Science & Technology* **55**, 1626-1636 (2021).

82. Luo D*, et al.* Plant Growth Promotion Driven by a Novel *Caulobacter* Strain. *Molecular Plant-Microbe Interactions* **32**, 1162-1174 (2019).

83. Sun L-N, Yang E-D, Wei J-C, Tang X-Y, Cao Y-Y, Han G-M. *Caulobacter flavus* sp. nov., a stalked bacterium isolated from rhizosphere soil. *International Journal of Systematic and Evolutionary Microbiology* **65**, 4374-4380 (2015).

84. Brown SD*, et al.* Twenty-One Genome Sequences from *Pseudomonas* Species and 19 Genome Sequences from Diverse Bacteria Isolated from the Rhizosphere and Endosphere of *Populus deltoides*. *Journal of Bacteriology* **194**, 5991-5993 (2012).

85. Liu L, Feng Y, Wei L, Zong Z, Kovac J. Genome-Based Taxonomy of *Brevundimonas* with Reporting *Brevundimonas huaxiensis* sp. nov. *Microbiology Spectrum* **9**, e00111-00121 (2021).

86. Karimi E*, et al.* Genome Sequences of 72 Bacterial Strains Isolated from *Ectocarpus subulatus*: A Resource for Algal Microbiology. *Genome Biology and Evolution* **12**, 3647-3655 (2020).

87. Fritz I, Strömpl C, Nikitin DI, Lysenko AM, Abraham W-R. *Brevundimonas mediterranea* sp. nov., a non-stalked species from the Mediterranean Sea. *International Journal of Systematic and Evolutionary Microbiology* **55**, 479-486 (2005).

88. Parks DH*, et al.* Recovery of nearly 8,000 metagenome-assembled genomes substantially expands the tree of life. *Nature Microbiology* **2**, 1533-1542 (2017).

89. Su H, Zhang T, Bao M, Jiang Y, Wang Y, Tan T. Genome Sequence of a Promising Hydrogen-Producing Facultative Anaerobic Bacterium, *Brevundimonas naejangsanensis* Strain B1. *Genome Announcements* **2**, e00542-00514 (2014).

90. Leifson E, Hugh R. A New Type of Polar Monotrichous Flagellation. *Journal of General Microbiology* **10**, 68-70 (1954).

91. Yoon J-H, Kang S-J, Lee J-S, Oh T-K. *Brevundimonas terrae* sp. nov., isolated from an alkaline soil in Korea. *International Journal of Systematic and Evolutionary Microbiology* **56**, 2915-2919 (2006).

92. Urakami T, Oyanagi H, Araki H, Suzuki KI, Komagata K. Recharacterization and Emended Description of the Genus *Mycoplana* and Description of Two New Species, *Mycoplana ramosa* and *Mycoplana segnis*. *International Journal of Systematic Bacteriology* **40**, 434-442 (1990).

93. Zimmermann J*, et al.* The functional repertoire contained within the native microbiota of the model nematode *Caenorhabditis elegans*. *The ISME Journal* **14**, 26-38 (2020).

94. Jiang L*, et al.* Pyomelanin-Producing *Brevundimonas vitisensis* sp. nov., Isolated From Grape (*Vitis vinifera* L.). *Frontiers in Microbiology* **12**, (2021).

95. Choi J-H, Kim M-S, Roh SW, Bae J-W. *Brevundimonas basaltis* sp. nov., isolated from black sand. *International Journal of Systematic and Evolutionary Microbiology* **60**, 1488-1492 (2010).

96. Yoon J-H, Kang S-J, Lee J-S, Oh HW, Oh T-K. *Brevundimonas lenta* sp. nov., isolated from soil. *International Journal of Systematic and Evolutionary Microbiology* **57**, 2236-2240 (2007).

97. Wang J, Zhang J, Ding K, Xin Y, Pang H. *Brevundimonas viscosa* sp. nov., isolated from saline soil. *International Journal of Systematic and Evolutionary Microbiology* **62**, 2475-2479 (2012).

98. Abraham W-R, Estrela AB, Nikitin DI, Smit J, Vancanneyt M. *Brevundimonas halotolerans* sp. nov., *Brevundimonas poindexterae* sp. nov. and *Brevundimonas staleyi* sp. nov., prosthecate bacteria from aquatic habitats. *International Journal of Systematic and Evolutionary Microbiology* **60**, 1837-1843 (2010).

99. Qu J-H, Fu Y-H, Li X-D, Li H-F, Tian H-L. *Brevundimonas lutea* sp. nov., isolated from lake sediment. *International Journal of Systematic and Evolutionary Microbiology* **69**, 1417-1422 (2019).

100. Ishizawa H, Kuroda M, Morikawa M, Ike M. Evaluation of environmental bacterial communities as a factor affecting the growth of duckweed *Lemna minor*. *Biotechnology for Biofuels* **10**, (2017).

101. Zhu L*, et al. Asticcacaulis endophyticus* sp. nov., a prosthecate bacterium isolated from the root of Geum aleppicum. *International Journal of Systematic and Evolutionary Microbiology* **64**, 3964-3969 (2014).

102. Pate JL, Porter JS, Jordan TL. *Asticcacaulis biprosthecum* sp.nov. Life cycle, morphology and cultural characteristics. *Antonie van Leeuwenhoek* **39**, 569-583 (1973).

103. Pongratz E. D'une bactérie pédiculée isolée d'un pus de sinus. *Pathobiology* **20**, 593-608 (1957).

104. Inoue K, Habe H, Yamane H, Omori T, Nojiri H. Diversity of carbazole-degrading bacteria having the *car* gene cluster: Isolation of a novel gram-positive carbazole-degrading bacterium. *FEMS Microbiology Letters* **245**, 145-153 (2005).

105. Lee SH, Shim JK, Kim JM, Choi H-K, Jeon CO. *Henriciella litoralis* sp. nov., isolated from a tidal flat, transfer of *Maribaculum marinum* Lai et al. 2009 to the genus

Henriciella as *Henriciella aquimarina* nom. nov. and emended description of the genus *Henriciella*. *International Journal of Systematic and Evolutionary Microbiology* **61**, 722-727 (2011).

106. Chertkov O*, et al.* Complete genome sequence of *Hirschia baltica* type strain (IFAM 1418T). *Standards in Genomic Sciences* **5**, 287-297 (2011).

107. Driscoll CB, Otten TG, Brown NM, Dreher TW. Towards long-read metagenomics: complete assembly of three novel genomes from bacteria dependent on a diazotrophic cyanobacterium in a freshwater lake co-culture. *Standards in Genomic Sciences* **12**, (2017).

108. Abraham W-R*, et al.* Phylogeny of *Maricaulis* Abraham et al. 1999 and proposal of *Maricaulis virginensis* sp. nov., *M. parjimensis* sp. nov., *M. washingtonensis* sp. nov. and *M. salignorans* sp. nov. *International Journal of Systematic and Evolutionary Microbiology* **52**, 2191-2201 (2002).

109. Lv X-L*, et al. Glycocaulis albus* sp. nov., a moderately halophilic dimorphic prosthecate bacterium isolated from petroleum-contaminated saline soil. *International Journal of Systematic and Evolutionary Microbiology* **64**, 3181-3187 (2014).

110. Cole JK*, et al.* Phototrophic biofilm assembly in microbial-mat-derived unicyanobacterial consortia: model systems for the study of autotroph-heterotroph interactions. *Frontiers in Microbiology* **5**, (2014).

111. Jeong SE, Jeon SH, Chun BH, Kim D-W, Jeon CO. *Marinicauda algicola* sp. nov., isolated from a marine red alga *Rhodosorus marinus*. *International Journal of Systematic and Evolutionary Microbiology* **67**, 3423-3427 (2017).

112. Zhai T-J, Liu B-T, Zhu R-Q, Chen G-J, Du Z-J. *Marinicauda salina* sp. nov., isolated from a marine solar saltern. *International Journal of Systematic and Evolutionary Microbiology* **69**, 2233-2238 (2019).

113. Nedashkovskaya OI, Kukhlevskiy AD, Zhukova NV, Kim S-J, Rhee S-K. *Litorimonas cladophorae* sp. nov., a new *alphaproteobacterium* isolated from the Pacific green alga *Cladophora stimpsoni*, and emended descriptions of the genus *Litorimonas* and *Litorimonas taeaensis*. *Antonie van Leeuwenhoek* **103**, 1263-1269 (2013).

114. Yu Z, Lai Q, Li G, Shao Z. *Parvularcula dongshanensis* sp. nov., isolated from soft coral. *International Journal of Systematic and Evolutionary Microbiology* **63**, 2114-2117 (2013).

115. Al-Omari J*, et al. Parvularcula mediterranea* sp. nov., isolated from marine plastic debris from Zakynthos Island, Greece. *International Journal of Systematic and Evolutionary Microbiology* **71**, (2021).

116. Arun AB*, et al. Parvularcula lutaonensis* sp. nov., a moderately thermotolerant marine bacterium isolated from a coastal hot spring. *International Journal of Systematic and Evolutionary Microbiology* **59**, 998-1001 (2009).

117. Zhang X-Q, Wu Y-H, Zhou X, Zhang X, Xu X-W, Wu M. *Parvularcula flava* sp. nov., an alphaproteobacterium isolated from surface seawater of the South China Sea.

International Journal of Systematic and Evolutionary Microbiology **66**, 3498-3502 (2016).

118. Ying J-J, Wu Z-C, Fang Y-C, Xu L, Sun C. Reclassification of *Parvularcula* flava as *Aquisalinus luteolus* nom. nov. and emended description of the genus *Aquisalinus*. *International Journal of Systematic and Evolutionary Microbiology* **71**, (2021).

119. Chu C*, et al. Phenylobacterium kunshanense* sp. nov., isolated from the sludge of a pesticide manufacturing factory. *International Journal of Systematic and Evolutionary Microbiology* **65**, 325-330 (2015).

120. Li X*, et al. Phenylobacterium soli* sp. nov., isolated from arsenic and cadmium contaminated farmland soil. *International Journal of Systematic and Evolutionary Microbiology* **69**, 1398-1403 (2019).

121. Choi G-M, Lee S-Y, Choi KD, Im W-T. *Phenylobacterium hankyongense* sp. nov., isolated from ginseng field soil. *International Journal of Systematic and Evolutionary Microbiology* **68**, 125-130 (2018).

122. Lingens F*, et al. Phenylobacterium immobile* gen. nov., sp. nov., a Gram-Negative Bacterium That Degrades the Herbicide Chloridazon. *International Journal of Systematic Bacteriology* **35**, 26-39 (1985).

123. Jo JH, Choi G-M, Lee S-Y, Im W-T. *Phenylobacterium aquaticum* sp. nov., isolated from the reservoir of a water purifier. *International Journal of Systematic and Evolutionary Microbiology* **66**, 3519-3523 (2016).

124. Gao J-l*, et al. Caulobacter zeae* sp. nov. and *Caulobacter radicis* sp. nov., novel endophytic bacteria isolated from maize root (*Zea mays* L.). *Systematic and Applied Microbiology* **41**, 604-610 (2018).

125. Gao J-L*, et al. Caulobacter endophyticus* sp. nov., an endophytic bacterium harboring three lasso peptide biosynthetic gene clusters and producing indoleacetic acid isolated from maize root. *Antonie van Leeuwenhoek* **114**, 1213-1224 (2021).

126. Moya G*, et al. Caulobacter hibisci* sp. nov., isolated from rhizosphere of *Hibiscus syriacus* L. (Mugunghwa flower). *International Journal of Systematic and Evolutionary Microbiology* **67**, 3167-3173 (2017).

127. Yang Y*, et al. Caulobacter soli* sp. nov., isolated from soil sampled at Jiri Mountain, Republic of Korea. *International Journal of Systematic and Evolutionary Microbiology* **70**, 4158-4164 (2020).

128. Ballard RW, Doudoroff M, Stanier RY, Mandel M. Taxonomy of the Aerobic Pseudomonads: *Pseudomonas diminuta* and *P. vesiculare*. *Journal of General Microbiology* **53**, 349-361 (1968).

129. Lee K, Son H, Choi Y. *Brevundimonas fontaquae* sp. nov., isolated from Dalgi carbonate spring-water. *Korean Journal of Microbiology* **58**, 275-285 (2022).

130. Li Y*, et al. Sphingomonas yabuuchiae* sp. nov. and *Brevundimonas nasdae* sp. nov., isolated from the Russian space laboratory Mir. *International Journal of Systematic and Evolutionary Microbiology* **54**, 819-825 (2004).

131. Kang SJ, Choi NS, Choi JH, Lee JS, Yoon JH, Song JJ. *Brevundimonas naejangsanensis* sp. nov., a proteolytic bacterium isolated from soil, and reclassification of *Mycoplana bullata* into the genus *Brevundimonas* as *Brevundimonas bullata* comb. nov. *International Journal of Systematic and Evolutionary Microbiology* **59**, 3155-3160 (2009).

132. Lee YW, Lee KH, Lee SY, Im W-T. *Brevundimonas fluminis* sp. nov., isolated from a river. *International Journal of Systematic and Evolutionary Microbiology* **70**, 204-210 (2020).

133. Ryu SH, Park M, Lee JR, Yun P-Y, Jeon CO. *Brevundimonas aveniformis* sp. nov., a stalked species isolated from activated sludge. *International Journal of Systematic and Evolutionary Microbiology* **57**, 1561-1565 (2007).

134. Moore RL, Weiner RM. Genus *Hyphomonas* (ex Pongratz 1975) Moore, Weiner and Gebers 1984, 71VP. In: *Bergey's Manual of Systematic Bacteriology* (eds Staley JT, Bryant MP, Pfenning N, Holt JG). Williams & Wilkins (1989).

135. Li C*, et al. Hyphomonas beringensis* sp. nov. and *Hyphomonas chukchiensis* sp. nov., isolated from surface seawater of the Bering Sea and Chukchi Sea. *Antonie van Leeuwenhoek* **106**, 657-665 (2014).

136. Li C, Lai Q, Li G, Sun F, Shao Z. *Hyphomonas atlanticus* sp. nov., isolated from the Atlantic Ocean and emended description of the genus *Hyphomonas*. *Systematic and Applied Microbiology* **37**, 423-428 (2014).

137. Li X, Li C, Lai Q, Li G, Sun F, Shao Z. *Hyphomonas pacifica* sp. nov., isolated from deep sea of the Pacific Ocean. *Antonie van Leeuwenhoek* **109**, 1111-1119 (2016).

138. Wu Y-H, Cheng H, Huo Y-Y, Jin X-B, Wang C-S, Xu X-W. *Henriciella pelagia* sp. nov., isolated from seawater. *International Journal of Systematic and Evolutionary Microbiology* **67**, 3020-3025 (2017).

139. Ren W-T, Cheng H, Wang C-S, Xu X-W, Zhou P, Wu Y-H. Physiological and genomic features of *Henriciella* with the description of *Henriciella mobilis* sp. nov. *International Journal of Systematic and Evolutionary Microbiology* **71**, (2021).

140. Kang HS, Lee SD. *Hirschia maritima* sp. nov., isolated from seawater. *International Journal of Systematic and Evolutionary Microbiology* **59**, 2264-2268 (2009).

141. Sun H, Hu Y, Zhou S, Zheng Y, Zhang X-H. *Glycocaulis profundi* sp. nov., a marine bacterium isolated from seawater of the Mariana Trench. *International Journal of Systematic and Evolutionary Microbiology* **70**, 814-819 (2020).

142. Deng W, Zhang Y, Xie X, Zhao Z, Fu Y. *Euryhalocaulis caribicus* gen. nov., sp. nov., a New Members of the Family *Hyphomonadaceae* Isolated from the Caribbean Sea. *Current Microbiology* **66**, 606-612 (2013).

143. Alain K, Tindall BJ, Intertaglia L, Catala P, Lebaron P. *Hellea balneolensis* gen. nov., sp. nov., a prosthecate alphaproteobacterium from the Mediterranean Sea. *International Journal of Systematic and Evolutionary Microbiology* **58**, 2511-2519 (2008).

144. Li S, Tang K, Liu K, Yu C-P, Jiao N. *Parvularcula oceanus* sp. nov., isolated from deep-sea water of the Southeastern Pacific Ocean. *Antonie van Leeuwenhoek* **105**, 245-251 (2014).

145. Sun L-L*, et al. Parvularcula marina* sp. nov., isolated from surface water of the South China Sea, and emended description of the genus *Parvularcula*. *International Journal of Systematic and Evolutionary Microbiology* **69**, 2571-2576 (2019).

146. Crosson S, McGrath PT, Stephens C, McAdams HH, Shapiro L. Conserved modular design of an oxygen sensory/signaling network with species-specific output. *Proceedings of the National Academy of Sciences* **102**, 8018-8023 (2005).

147. Graham ED, Heidelberg JF, Tully BJ. Potential for primary productivity in a globally-distributed bacterial phototroph. *The ISME Journal* **12**, 1861-1866 (2018).

148. Jain C, Rodriguez-R LM, Phillippy AM, Konstantinidis KT, Aluru S. High throughput ANI analysis of 90K prokaryotic genomes reveals clear species boundaries. *Nature Communications* **9**, (2018).

149. Kim D, Park S, Chun J. Introducing EzAAI: a pipeline for high throughput calculations of prokaryotic average amino acid identity. *Journal of Microbiology* **59**, 476-480 (2021).
