# Supplementary material for: Widespread potential for phototrophy and convergent reduction of lifecycle complexity in the dimorphic order Caulobacterales
Source: Nat Commun. 2025 Dec 12;16:11003. doi: 10.1038/s41467-025-65642-x (PMC12700997; doi:10.1038/s41467-025-65642-x)
Supplement: Supplementary file 2 — Description of Additional Supplementary Files [file 41467_2025_65642_MOESM2_ESM.pdf]

SUPPLEMENTARY MOVIE LEGENDS

Supplementary Movie 1. Timelapse microscopy of *P. immobile* E^T at 30°C on R2A 1% agarose, in 5-minute imaging intervals. Playback speed: 18 frames/s (1.5 h/s). Scale bars: 1 µm. File format: .mp4. **(a)** The cell shown in **Fig**. **3a**. **(b)** Three additional cells.

Supplementary Movie 2. Timelapse microscopy of *E. coli* MG1655 at 37°C on LB 1% agarose, in 20-second imaging intervals. Playback speed: 30 frames/s (15 min/s). Scale bars: 1 µm. File format: .mp4. **(a)** The cell shown in **Fig**. **3a**. **(b)** Three additional cells.

Supplementary Movie 3. Timelapse microscopy of *C. crescentus* CB15 at 30°C on PYE 1% agarose, in 30-second imaging intervals. Playback speed: 30 frames/s (15 min/s). Scale bars: 1 µm. File format: .mp4. **(a)** The cell shown in **Fig**. **3a**. **(b)** Six additional cells.

SUPPLEMENTARY DATA LEGENDS

Supplementary Data 1 | Genome overview. Overview of genomes used in this work. **(a)** The "core dataset" of *Caulobacterales* genomes. Columns A–C, genome name, accession, and taxon name; Columns D–G, information on genome dereplication and selection of species representatives; Column H, GTDB taxonomy (release R207); Columns I–R, genome statistics, including assembly size, estimated genome size, N50, number of contigs, G+C content, and estimates of completeness and contamination from both the CheckM[33] methods 'taxonomy_wf' (used for genome selection) and 'lineage_wf' (used for estimated genome size calculation); Columns S–U, additional information. **(b)** The "extended dataset" of *Acaudatibacter* gen. nov. (GTDB taxon "g__Palsa-881") genomes sourced from Rodríguez-Gijón *et al.*[36] Column A, genome name; Column B, references for assemblies[36-38, 73-76]; Column C–D, species clustering information; Column E, whether the genome is also included as a species genome representative (SGR) in the "core dataset"; Column F, type of genome (MAG, metagenome-assembled genome); Column G, GTDB taxonomy (release R207); Columns H–N, genome statistics, including assembly size, estimated genome size, number of contigs, number of scaffolds, G+C content, and estimates of completeness and contamination from the CheckM method 'lineage_wf'.

Supplementary Data 2 | Environmental metadata. Meta analysis of genome sampling environment metadata. **(a)** Explanation of the table layout and content of the metadata. **(b)** Manually collected and curated metadata from NCBI BioSample (www.ncbi.nlm.nih.gov/biosample/) and BioProject (www.ncbi.nlm.nih.gov/bioproject/) pages, as well as JGI Gold (gold.jgi.doe.gov/), and listed publications when necessary. Listed literature references: [2, 6, 7, 9-12, 17, 21, 22, 28, 43, 46, 52, 54, 55, 58, 62, 64-66, 77-118].

Supplementary Data 3 | IMNGS environmental data. Compiled IMNGS[32] 'Taxonomy' job results for the query: "Bacteria/Proteobacteria/Alphaproteobacteria/Caulobacterales/Caulobacteraceae".

Supplementary Data 4 | Selected gene presence/absence data. Gene presence and absence data presented in figures and supplementary figures of the article (**Figs. 2b**, **4b**, and **5b**, **Supplementary Figs. S6**, **S8**, and **S16**, and **Supplementary Data 5** and **8)**. Includes genes for chemotaxis, flagellar motility, cell cycle and development, type IV pilus, holdfast synthesis, crescentin, S-layer, prostheca, cell division, carotenoid synthesis, photosynthesis, carbon fixation, aerobic respiration, secretion systems, and sulfonate transport. Includes both *Caulobacterales* genomes of the "core dataset", and *Acaudatibacter* genomes of the "extended dataset". For gene orthologs, attributes are separated by "@" in the following order: (1) gene category, (2) annotation tool, (3) KEGG KO or *C. crescentus* locus IDs, (4) gene name, (5) EC number, (6) gene annotation, (7) manually curated gene name. For RBH results, the attributes #4 and #7 were taken from the *C. crescentus* NA1000 GCF_000022005.1 assembly, since it is better annotated, but they all agree well with the *C. crescentus* CB15 GCF_000006905.1 assembly. For genomes, attributes are separated by "@" in the following order: (1) dataset [either the "core" dataset of *Caulobacterales* species genome representatives or the "extended" dataset of additional *Acaudatibacter*/"Palsa-881" genomes], (2) assembly ID, (3) family, (4) genus, (5) species, (6) taxon name for the genome assembly [only for the "extended" dataset]. KOs of the "core" dataset were annotated using eggNOG-mapper[39] (emapper) v2.1.5 or GhostKOALA[42] v2.3 and KOs of the "extended" dataset were annotated using eggNOG-mapper (emapper) v2.1.12 or GhostKOALA v3.0.

Supplementary Data 5 | Overview of the presence and absence of cell morphology and development genes across *Caulobacterales*. (a) Species phylogeny shown in **Fig. 1a**. Numbers

represent non-parametric bootstraps and the scale bar indicates number of substitutions per site. (**b–j**) Expanded view of the presence and absence of genes presented in **Fig. 2b**, showing genes involved in (b) chemotaxis, (c) flagellum, (d) cell cycle and developmental genes, (e) type IV adhesive pilus (T4P), (f) holdfast, (g) crescentin, (h) S-layer, (i) prostheca, and (j) cell division, among *Caulobacterales* genomes. Gene orthologs were identified using KEGG ortholog (KO) annotations from eggNOG-mapper[39] v2.1.5 (dark gray) or through the reciprocal best blast hit (RBH) algorithm using the *C. crescentus* CB15 proteome (blue). For RBH results, the corresponding loci in the *C. crescentus* CB15 (CC numbers) and *C. crescentus* NA1000 (CCNA numbers) are shown alongside the gene name. Descriptions come from the KO annotation or from the *C. crescentus* NA1000 genome annotation. Numbers show KO copy numbers > 1. The full dataset is found in **Supplementary Data 4**.

Supplementary Data 6 | Uncharacterized putative flagellar/developmental genes. Identification of putative flagellar motility and development factors, based on their absence from non-flagellated *Acaudatibacter* gen. nov. and *Phenylobacterium* species. Presence/absence is based on the reciprocal best blast hit (RBH) algorithm. The 100 genes missing from non-flagellated lineages, sorted by their conservation in the *Acaudatibacter–Caulobacter–Phenylobacterium* (ACP) clade. For gene orthologs, attributes are separated by "@" in the following order: (1) gene category, (2) *C. crescentus* CB15 protein accession, (3) CB15 new locus ID, (4) CB15 old locus ID, (5) CB15 gene name, (6) CB15 gene annotation, (7) *C. crescentus* NA1000 protein accession, (8) NA1000 locus ID, (9) NA1000 gene name, (10) NA1000 gene annotation, (11) total number of species genome representatives having the gene within the ACP clade [basis for sorting]. For genomes, attributes are separated by "@" as presented in **Supplementary Data 4**.

Supplementary Data 7 | Colony pigments. Meta analysis of the colony pigment description terms used in the literature for *Caulobacterales* isolates included in our dataset, if available. Listed literature references: [1-4, 6, 7, 9, 17, 21, 22, 28, 29, 43-46, 48-52, 54-63, 65, 66, 79, 80, 83, 85, 87, 90-92, 94-99, 101, 102, 108, 109, 111-113, 115, 117, 119-145].

Supplementary Data 8 | Overview of the presence and absence of phototrophy and respiration genes across *Caulobacterales*. (a) Species phylogeny shown in **Fig. 1a**. Numbers represent non-parametric bootstraps and the scale bar indicates number of substitutions per site. Genomes containing phototrophy genes are marked with red circles. **(b)** Meta-analysis of colony pigments across *Caulobacterales* species. See **Supplementary Data 7** for colony descriptor words and references. Asterisks: *Caulobacter* isolates ErkDOM-C and ErkDOME pigment descriptions derive from this work. **(c–i)** Expanded view of the presence and absence of genes presented in **Fig. 5b**, showing genes involved in (c) carotenoid biosynthesis, (d) bacteriochlorophyll biosynthesis, (e) bacteriochlorophyll transport, (f) light-harvesting complex II (LH2), (g) reaction center–light-harvesting complex I (RC–LH1), (h) CO_2 fixation using the CBB cycle, and (i) aerobic respiration among *Caulobacterales* genomes. KEGG ortholog (KO) gene ortholog were annotated using either eggNOG-mapper[39] (emapper; v2.1.5) or GhostKOALA[42] (v2.2). Numbers show KO copy numbers > 1. Abbreviations: cytochrome (cyt.). Full dataset is found in **Supplementary Data 4**. **(j)** Schematic representation of the highly branched electron transport chain of *C. crescentus* CB15, which includes two high-affinity terminal oxidases operating under low-oxygen concentrations (cytochromes *bd* and bb_3) and two low-affinity terminal oxidases operating under high-oxygen concentrations (cytochromes bo_3 and aa_3)[146].

Supplementary Data 9 | Overview of the presence and absence of carbon fixation pathways across *Caulobacterales*. Estimated completeness of carbon fixation pathways among *Caulobacterales*

genomes based on KEGG ortholog (KO) annotations from eggNOG-mapper[39] (v2.1.5) and associated KEGG modules. **(a)** Species phylogeny shown in **Fig. 1a**. Numbers represent non-parametric bootstraps and the scale bar indicates number of substitutions per site. Genomes containing phototrophic potential are marked with red circles for *Caulobacterales* and with dark blue circles for outgroup *Alphaproteobacteria*. Genomes with complete genetic potential for the CBB cycle are marked with orange circles. **(b)** Calvin-Benson-Bassham (CBB) cycle—KEGG module M00165. *Left panel*: Completeness of the CBB cycle steps. *Right panel*: Copy number of individual CBB cycle KOs. **(c)** Completeness of the reductive citrate cycle steps—KEGG module M00173. **(d)** Completeness of the 3-hydroxy-propionate bicycle steps—KEGG module M00376.

Supplementary Data 10 | Overview of eggNOG-mapper-predicted NOGs. Overview of eggNOG-mapper[39] (emapper) annotations of non-supervised orthologous groups (NOGs) in *Caulobacterales* species genome representatives of the "core dataset" (emapper v2.1.5). For each genome, attributes are separated by "@" as presented in **Supplementary Data 4**.

Supplementary Data 11 | Overview of eggNOG-mapper-predicted KOs. Overview of eggNOG-mapper[39] (emapper) KEGG ortholog (KO) annotations of **(a)** *Caulobacterales* species genome representatives of the "core dataset" (emapper v2.1.5), **(b)** *Acaudatibacter* gen. nov. ("Palsa-881") genomes of the "extended dataset" (emapper v2.1.12), or **(c)** genomes of curved crescentin-encoding *Hyphomicrobiales* species (emapper v2.1.12). For each genome, attributes are separated by "@" as presented in **Supplementary Data 4**.

Supplementary Data 12 | Overview of GhostKOALA-predicted KOs. Overview of GhostKOALA[42] v2.2 KEGG ortholog (KO) annotations of **(a)** *Caulobacterales* species genome representatives of the "core dataset" (GhostKOALA v2.3), or **(b)** *Acaudatibacter* gen. nov. ("Palsa-881") genomes of the "extended dataset" (GhostKOALA v3.0). For each genome, attributes are separated by "@" as presented in **Supplementary Data 4**.

Supplementary Data 13 | KEGG Decoder: pathway completeness. Pathway completeness prediction using KEGG Decoder[147] v1.3 with GhostKOALA-predicted KOs listed in **Supplementary Data 12**.

Supplementary Data 14 | Overview of RBHs. Overview of reciprocal best blast hit (RBH) results for the *C. crescentus* CB15 proteome of the GCF_000006905.1 assembly queried against **(a)** *Caulobacterales* species genome representatives of the "core dataset", and **(b)** *Acaudatibacter* gen. nov. ("Palsa-881") genomes of the "extended dataset". For each genome, attributes are separated by "@" as presented in **Supplementary Data 4**.

Supplementary Data 15 | Pairwise ANI. Overview of pairwise average nucleotide identity (ANI) comparisons using FastANI[148] v1.33 for *Caulobacterales* genomes of the "core dataset" as well as *Acaudatibacter* gen. nov. ("Palsa-881") species of the "extended dataset". For each genome, attributes are separated by "@" as presented in **Supplementary Data 4**. Note that FastANI simply outputs "NA" for ANIs far below 80%.

Supplementary Data 16 | Pairwise AAI. Overview of pairwise average amino acid identity (AAI) comparisons using EzAAI[149] v1.2.3 for *Caulobacterales* genomes of the "core dataset" as well as *Acaudatibacter* gen. nov. ("Palsa-881") species of the "extended dataset". For each genome, attributes are separated by "@" as presented in **Supplementary Data 4**.

Supplementary Data 17 | Manual refinement of species phylogeny. Sequences manually removed when making the manually refined ML species phylogeny presented in **Fig. 1a** and **Supplementary Fig. S1**, which included the removal of putative paralogs, contamination, long-branching, horizontal transfers, and duplicate sequences.

Supplementary Data 18 | 16S and 23S rRNA gene hits from Barrnap. Overview of the longest 16S and 23S rRNA gene sequences retrieved from *Caulobacterales* species representative genomes using Barrnap v0.9 (https://github.com/tseemann/barrnap), with a cutoff of 50% of each gene ("--reject 0.5"), and using bacterial rRNA gene models ("--kingdom bac"), and which were then used to infer the species phylogeny presented in **Supplementary Fig. S3**. "NA" means that no sequence was retrieved.
