# Supplementary material for: Widespread potential for phototrophy and convergent reduction of lifecycle complexity in the dimorphic order Caulobacterales
Source: Nat Commun. 2025 Dec 12;16:11003. doi: 10.1038/s41467-025-65642-x (PMC12700997; doi:10.1038/s41467-025-65642-x)
Supplement: Supplementary file 6 — Supplementary Data 1–18 [file 41467_2025_65642_MOESM6_ESM.zip › Supplementary Data 5, 8 and 9.pdf]

SUPPLEMENTARY DATA 5, 8, and 9

Widespread potential for phototrophy and convergent reduction of lifecycle complexity within the dimorphic order *Caulobacterales*

Joel Hallgren[1], Jennah E. Dharamshi[2,5], Alejandro Rodríguez-Gijón[2], Julia Nuy[2,6], Sarahi L. Garcia[2,3,4], and Kristina Jonas[1*]

[1] Department of Molecular Biosciences, The Wenner-Gren Institute, Science for Life Laboratory, Stockholm University, Stockholm, Sweden.

[2] Department of Ecology, Environment and Plant Sciences, Science for Life Laboratory, Stockholm University, Stockholm, Sweden.

[3] Institute for Chemistry and Biology of the Marine Environment (ICBM), Carl von Ossietzky University of Oldenburg, Oldenburg, Germany

[4] Helmholtz Institute for Functional Marine Biodiversity at the University of Oldenburg (HIFMB, Oldenburg, Germany

[5] Present address: Department of Organismal Biology, Program in Systematic Biology, Uppsala University, Uppsala, Sweden.

[6] Present address: Environmental Metagenomics, Research Center One Health, University of Duisburg-Essen, Essen, Germany.

*Corresponding author, kristina.jonas@su.se

SUPPLEMENTARY DATA 5 3

SUPPLEMENTARY DATA 8 6

SUPPLEMENTARY DATA 9 9

REFERENCES 12

SUPPLEMENTARY DATA 5

Supplementary Data 5

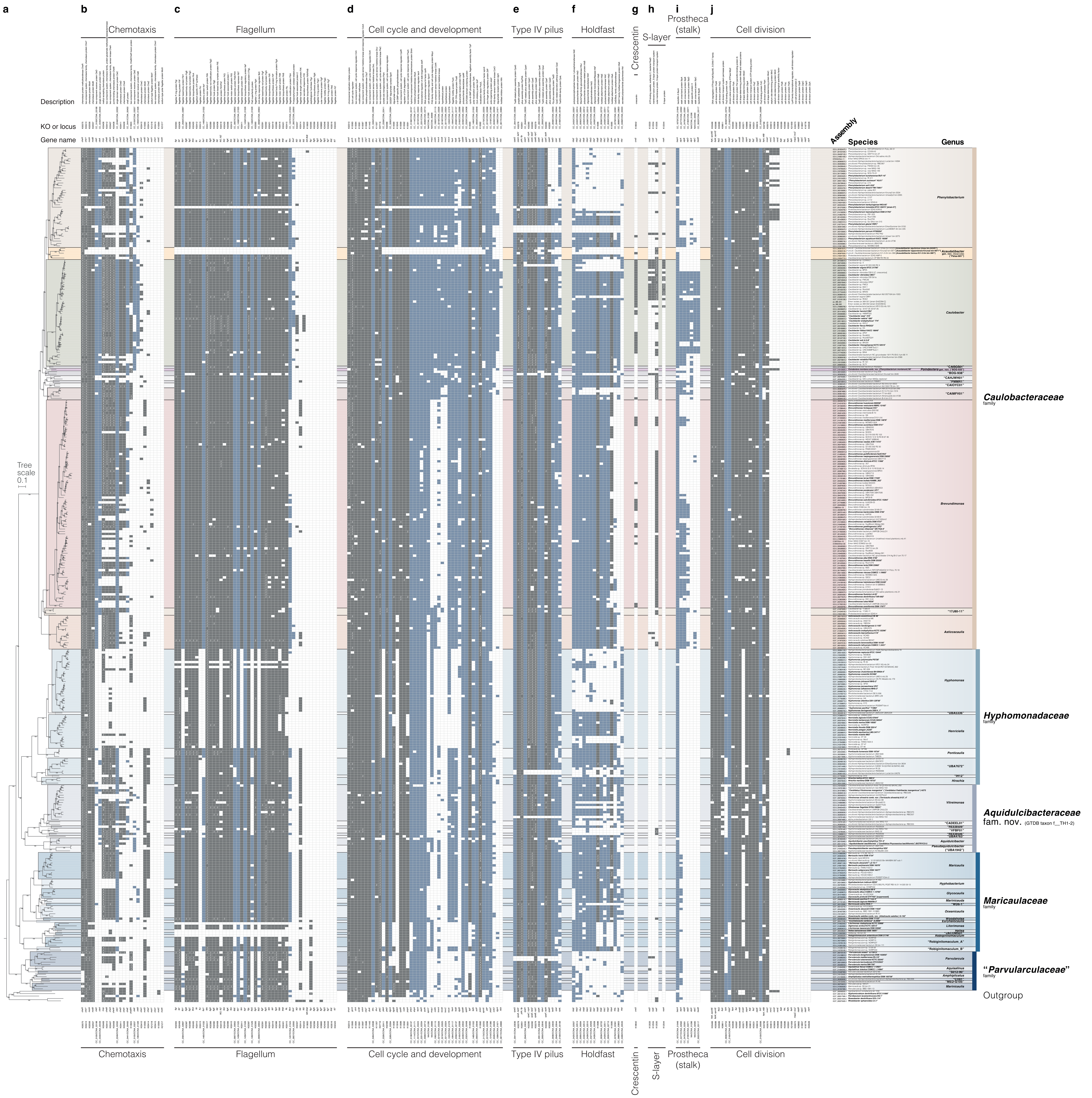

Supplementary Data 5 | Overview of the presence/absence of cell morphology and development genes across *Caulobacterales*. **(a)** Species phylogeny shown in **Fig. 1a**. Numbers represent non-parametric bootstraps and the scale bar indicates number of substitutions per site. (**b–j**) Expanded view of the presence and absence of genes presented in **Fig. 2b**, showing genes involved in (b) chemotaxis, (c) flagellum, (d) cell cycle and developmental genes, (e) type IV adhesive pilus (T4P), (f) holdfast, (g) crescentin, (h) S-layer, (i) prostheca, and (j) cell division, among *Caulobacterales* genomes. Gene orthologs were identified using KEGG ortholog (KO) annotations from eggNOG-mapper[32] v2.1.5 (dark gray) or through the reciprocal best blast hit (RBH) algorithm using the *C. crescentus* CB15 proteome (blue). For RBH results, the corresponding loci in the *C. crescentus* CB15 (CC numbers) and *C. crescentus* NA1000 (CCNA numbers) are shown alongside the gene name. Descriptions come from the KO annotation or from the *C. crescentus* NA1000 genome annotation. Numbers show KO copy numbers > 1. The full dataset is found in **Supplementary Data 4**.

SUPPLEMENTARY DATA 8

Supplementary Data 8

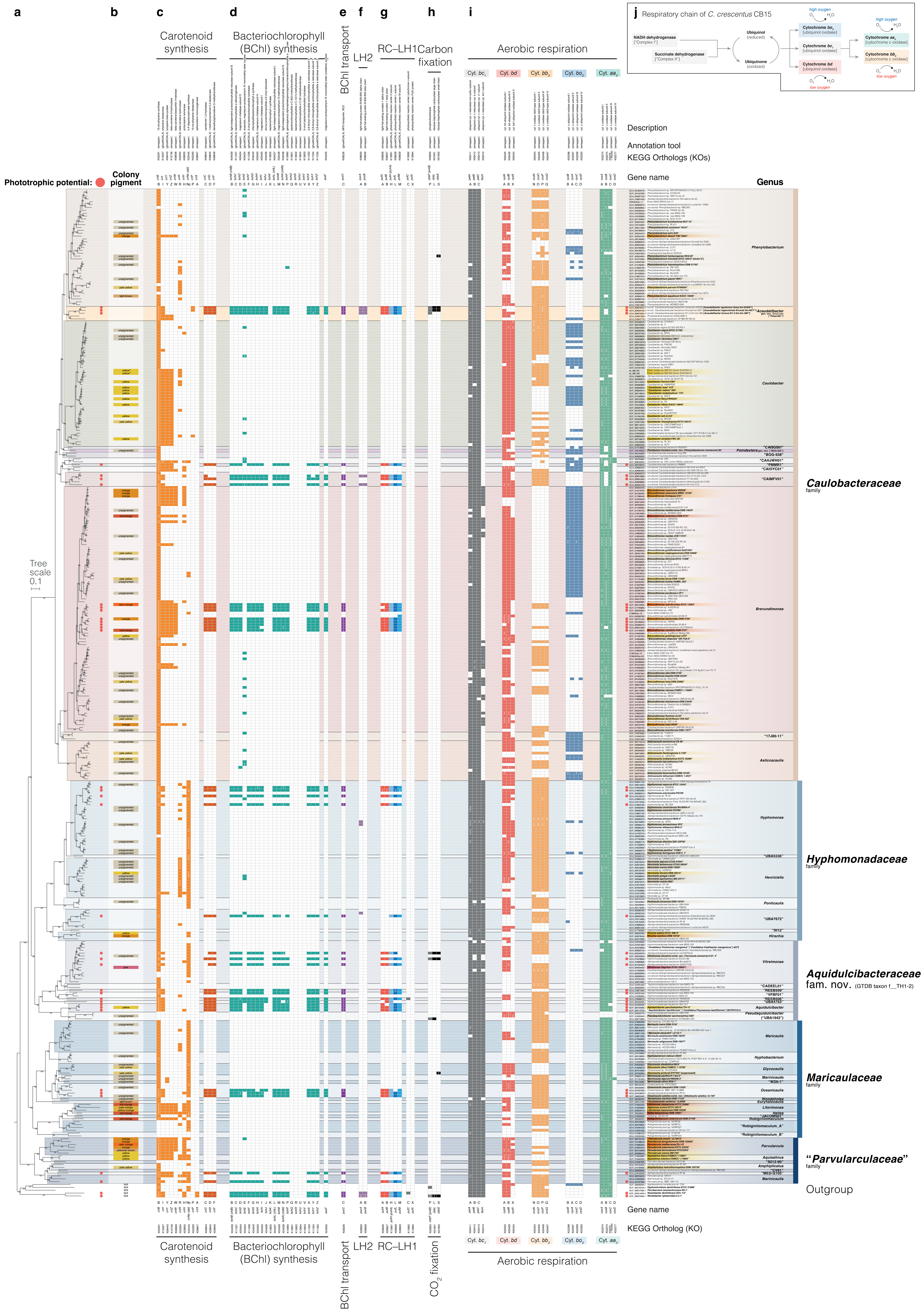

Supplementary Data 8 | Overview of the presence/absence of phototrophy and respiration genes across *Caulobacterales*. (a) Species phylogeny shown in **Fig. 1a**. Numbers represent non-parametric bootstraps and the scale bar indicates number of substitutions per site. Genomes containing phototrophy genes are marked with red circles. **(b)** Meta-analysis of colony pigments across *Caulobacterales* species. See **Supplementary Data 7** for colony descriptor words and references. Asterisks: *Caulobacter* isolates ErkDOM-C and ErkDOME pigment descriptions derive from this work. **(c–i)** Expanded view of the presence and absence of genes presented in **Fig. 5b**, showing genes involved in (c) carotenoid biosynthesis, (d) bacteriochlorophyll biosynthesis, (e) bacteriochlorophyll transport, (f) light-harvesting complex II (LH2), (g) reaction center–light-harvesting complex I (RC–LH1), (h) CO_2 fixation using the CBB cycle, and (i) aerobic respiration among *Caulobacterales* genomes. KEGG ortholog (KO) gene ortholog were annotated using either eggNOG-mapper[32] (emapper; v2.1.5) or GhostKOALA[38] (v2.2). Numbers show KO copy numbers > 1. Abbreviations: cytochrome (cyt.). Full dataset is found in **Supplementary Data 4**. **(j)** Schematic representation of the highly branched electron transport chain of *C. crescentus* CB15, which includes two high-affinity terminal oxidases operating under low-oxygen concentrations (cytochromes *bd* and bb_3) and two low-affinity terminal oxidases operating under high-oxygen concentrations (cytochromes bo_3 and aa_3)[39].

SUPPLEMENTARY DATA 9

Supplementary Data 9

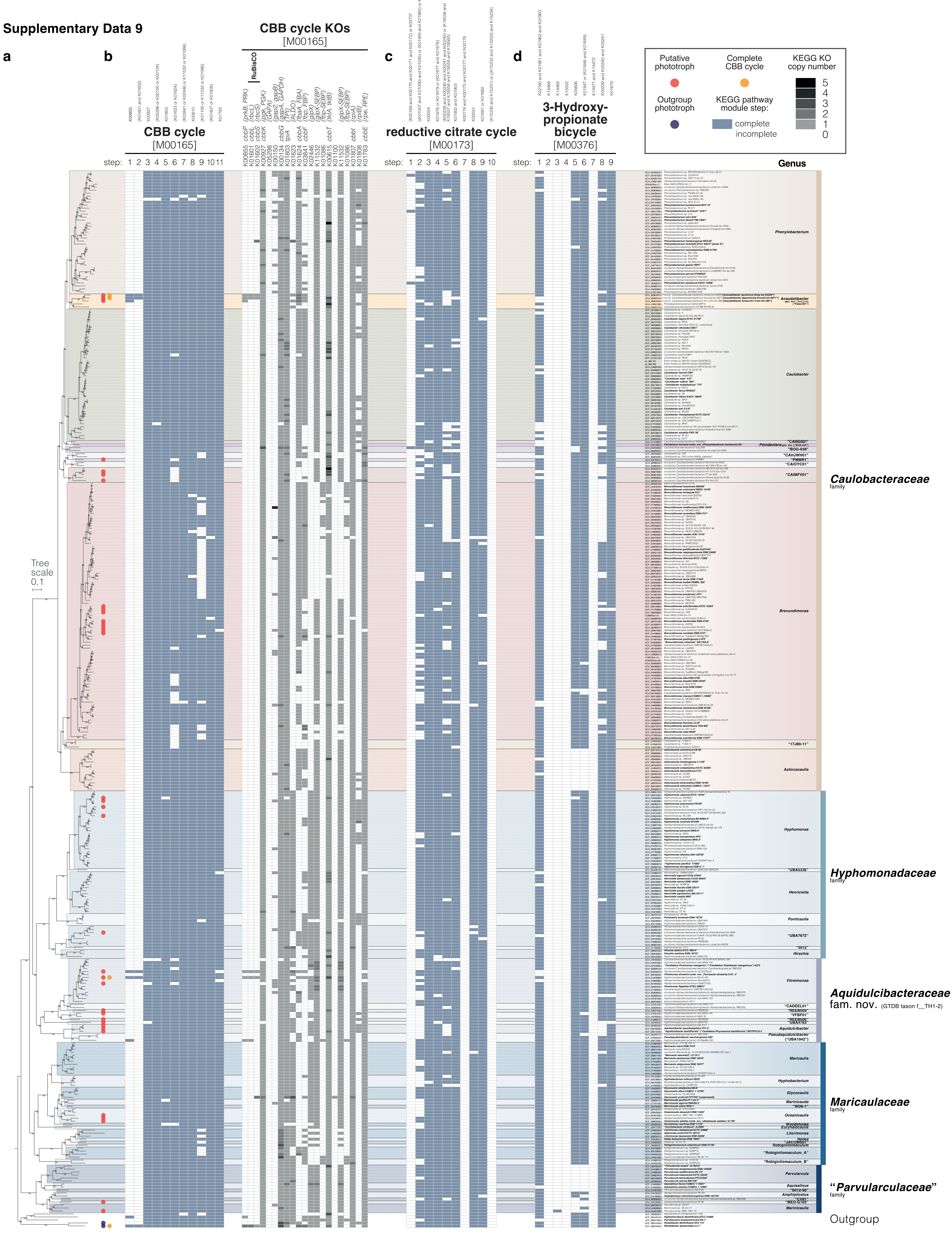

Supplementary Data 9 | Estimated completeness of carbon fixation pathways among *Caulobacterales* genomes based on KEGG ortholog (KO) annotations from eggNOG-mapper[32] (v2.1.5) and associated KEGG modules. (a) Species phylogeny shown in **Fig. 1a**. Numbers represent non-parametric bootstraps and the scale bar indicates number of substitutions per site. Genomes containing phototrophic potential are marked with red circles for *Caulobacterales* and with dark blue circles for outgroup *Alphaproteobacteria*. Genomes with complete genetic potential for the CBB cycle are marked with orange circles. **(b)** Calvin-Benson-Bassham (CBB) cycle—KEGG module M00165. *Left panel*: Completeness of the CBB cycle steps. *Right panel*: Copy number of individual CBB cycle KOs. **(c)** Completeness of the reductive citrate cycle steps—KEGG module M00173. **(d)** Completeness of the 3-hydroxy-propionate bicycle steps—KEGG module M00376.

REFERENCES

References can be found in ***Supplementary Information****.*
