# Supplementary material for: Widespread potential for phototrophy and convergent reduction of lifecycle complexity in the dimorphic order Caulobacterales
Source: Nat Commun. 2025 Dec 12;16:11003. doi: 10.1038/s41467-025-65642-x (PMC12700997; doi:10.1038/s41467-025-65642-x)
Supplement: Supplementary file 7 — Reporting Summary [file 41467_2025_65642_MOESM7_ESM.pdf]

Corresponding author(s): Kristina Jonas
Last updated by author(s): Oct 7, 2025

Reporting Summary

Nature Portfolio wishes to improve the reproducibility of the work that we publish. This form provides structure for consistency and transparency in reporting. For further information on Nature Portfolio policies, see our Editorial Policies and the Editorial Policy Checklist.

Statistics

For all statistical analyses, confirm that the following items are present in the figure legend, table legend, main text, or Methods section.

n/a	Confirmed	
☐	☒	The exact sample size (*n*) for each experimental group/condition, given as a discrete number and unit of measurement
☐	☒	A statement on whether measurements were taken from distinct samples or whether the same sample was measured repeatedly
☐	☒	The statistical test(s) used AND whether they are one- or two-sided<br>*Only common tests should be described solely by name; describe more complex techniques in the Methods section.*
☒	☐	A description of all covariates tested
☒	☐	A description of any assumptions or corrections, such as tests of normality and adjustment for multiple comparisons
☐	☒	A full description of the statistical parameters including central tendency (e.g. means) or other basic estimates (e.g. regression coefficient) AND variation (e.g. standard deviation) or associated estimates of uncertainty (e.g. confidence intervals)
☐	☒	For null hypothesis testing, the test statistic (e.g. *F*, *t*, *r*) with confidence intervals, effect sizes, degrees of freedom and *P* value noted<br>*Give P values as exact values whenever suitable.*
☒	☐	For Bayesian analysis, information on the choice of priors and Markov chain Monte Carlo settings
☒	☐	For hierarchical and complex designs, identification of the appropriate level for tests and full reporting of outcomes
☒	☐	Estimates of effect sizes (e.g. Cohen's *d*, Pearson's *r*), indicating how they were calculated

Our web collection on statistics for biologists contains articles on many of the points above.

Software and code

Policy information about availability of computer code

Data collection	Nikon Elements v5.30.02; TECAN SparkControl magellan 2.2
Data analysis	Microsoft Excel for Mac v16.92; R v4.4.0; RStudio v2024.04.0.735; GraphPad Prism v10.3.0; iTOL; Fiji (ImageJ) v2.1.0/1.53c with MicrobeJ v5.13l; CheckM v1.1.3; dRep v3.2.2; Prokka v1.14.6; GhostKOALA v2.2 and v3.0; eggNOG-mapper v2.1.5 and v2.1.12; KEGG Decoder v1.3; InterProScan v5.52.-86.0; DIAMOND blastp v2.0.9; fastANI v1.33; EzAAI v1.2.3; Quast v5.0.2; blastp v2.15.0+; Mauve snapshot_2015-02-25 build0; GToTree v1.6.20 and v1.8.6; Prodigal v2.6.3; HMMER3 v3.3.2; Muscle v5.1.linux64; TrimAl v1.4.rev15 and v1.4.1; IQ-TREE v1.6.12, v2.2.0, and v2.2.2.6; Barrnap v0.9; MAFFT L-INS-i and E-INS-i v7.407; PhyKIT v1.11.7; Clustal Omega; Jalview v2.11.4.1; EDirect v15.1; CD-HIT v4.8.1; FAMSA v2.2.2<br><br>Custom code:<br>https://github.com/jennahd/anno-utils<br>https://github.com/jennahd/tree-utils<br>https://github.com/j-hallgren/make-RBH

For manuscripts utilizing custom algorithms or software that are central to the research but not yet described in published literature, software must be made available to editors and reviewers. We strongly encourage code deposition in a community repository (e.g. GitHub). See the Nature Portfolio guidelines for submitting code & software for further information.

Data

Policy information about availability of data

All manuscripts must include a data availability statement. This statement should provide the following information, where applicable:

- Accession codes, unique identifiers, or web links for publicly available datasets
- A description of any restrictions on data availability
- For clinical datasets or third party data, please ensure that the statement adheres to our policy

Genome data used in this work is publicly available and was obtained from NCBI RefSeq (https://www.ncbi.nlm.nih.gov/refseq/), NCBI Genbank (https://www.ncbi.nlm.nih.gov/genbank/), the JGI Genome Portal (https://genome.jgi.doe.gov/portal/), Lake Erken model community MAGs (https://doi.org/10.17044/scilifelab.19923161.v1), and from sequenced Lake Erken Caulobacter isolates made available at Genbank (BioProject accession: PRJNA1228543; assemblies: GCA_048541825.1, GCA_048541895.1, and GCA_048541805.1). Additional data supporting the findings of this work is provided in the Supplementary Information, and as a tabular data file containing Supplementary Data 1–4, 6–7, and 10–18, as a PDF file containing Supplementary Data 5 and 8–9, and as three .avi files containing Supplementary Movies 1–3. Additional raw data files, including genome annotations, sequence alignments, and phylogenetic tree files have been made publicly available at Researchdata.se hosted by the Swedish National Data Service (https://doi.org/10.58141/0bz5-dc62). Source Data are provided with this paper for data underlying graphs and charts.

Research involving human participants, their data, or biological material

Policy information about studies with human participants or human data. See also policy information about sex, gender (identity/presentation), and sexual orientation and race, ethnicity and racism.

Reporting on sex and gender	no human research participants present in this study
Reporting on race, ethnicity, or other socially relevant groupings	see above
Population characteristics	see above
Recruitment	see above
Ethics oversight	see above

Note that full information on the approval of the study protocol must also be provided in the manuscript.

Field-specific reporting

Please select the one below that is the best fit for your research. If you are not sure, read the appropriate sections before making your selection.

☐ Life sciences ☐ Behavioural & social sciences ☒ Ecological, evolutionary & environmental sciences

For a reference copy of the document with all sections, see nature.com/documents/nr-reporting-summary-flat.pdf

Ecological, evolutionary & environmental sciences study design

All studies must disclose on these points even when the disclosure is negative.

Study description	Phylogenomics, comparative genomics, and cell biological analyses, as well as meta-analyses of Caulobacterales bacteria.
Research sample	Available genomes and genome metadata of Caulobacterales bacteria. Available strains of Caulobacterales bacteria. Published literature on Caulobacterales bacteria.
Sampling strategy	Genomes were selected based on their phylogenetic affiliation with Caulobacterales according to GTDB Release 07-RS207. Genomes with estimated completeness above 95% and contamination below 5% were kept for analysis. Species representative genomes were selected based on the highest quality genome scores per species cluster (95% average nucleotide identity), with a few exceptions made to keep species type strains instead as species representatives as outlined in Methods.
Data collection	Three new bacterial genomes were sequenced during this study using the Sequel (PacBio) long-read sequencing system. DNA extraction was done by J. Hallgren. Sequencing and genome assembly was done at the National Genomics Infrastructure in Uppsala (Sweden). Otherwise all genome data stem from other parties and were publicly available at the start of the study; these data were downloaded by J.E. Dharamshi. Timelapse microscopy experiments and image analysis were done for bacterial strains by J. Hallgren as outlined in Methods. Publicly available genome metadata were manually collected by J. Hallgren from databases such as NCBI Biosample (https://www.ncbi.nlm.nih.gov/biosample/) and Bioproject (https://www.ncbi.nlm.nih.gov/bioproject/), and JGI GOLD platform (https://gold.jgi.doe.gov/). Moreover, when relevant metadata were absent in the aforementioned public databases, the information was manually compiled by J. Hallgren from published articles as indicated in Supplementary Data S2.

Timing and spatial scale	Publicly available genomes were downloaded in April 2022. Genome metadata were collected between May and June 2024.
Data exclusions	Genomes with estimated completeness below 95% and estimated contamination above 5% (CheckM v1.3.3 'lineage_wf') were excluded. For timelapse microscopy experiments, cells that did not exhibit growth were excluded from the analyses.
Reproducibility	The results of the study can be reproduced given the same original source data/material and the methods outlined in the manuscript.
Randomization	N/A. Randomization was not required for the purposes of the study.
Blinding	N/A. Blinding was not required for the purposes of this study.

Did the study involve field work? ☐ Yes ☒ No

Reporting for specific materials, systems and methods

We require information from authors about some types of materials, experimental systems and methods used in many studies. Here, indicate whether each material, system or method listed is relevant to your study. If you are not sure if a list item applies to your research, read the appropriate section before selecting a response.

Materials & experimental systems

n/a	Involved in the study
☒	☐ Antibodies
☒	☐ Eukaryotic cell lines
☒	☐ Palaeontology and archaeology
☐	☒ Animals and other organisms
☒	☐ Clinical data
☒	☐ Dual use research of concern
☒	☐ Plants

Methods

n/a	Involved in the study
☒	☐ ChIP-seq
☒	☐ Flow cytometry
☒	☐ MRI-based neuroimaging

Animals and other research organisms

Policy information about studies involving animals; ARRIVE guidelines recommended for reporting animal research, and Sex and Gender in Research

Laboratory animals	The study did not involve laboratory animals.
Wild animals	The study did not involve wild animals.
Reporting on sex	Not applicable to this study.
Field-collected samples	The study did not involve samples collected from the field.
Ethics oversight	No ethical approval or guidance was required, since the study did not involve humans or other animals. All laboratory work involving organisms was done using bacteria.

Note that full information on the approval of the study protocol must also be provided in the manuscript.

Plants

Seed stocks	*Report on the source of all seed stocks or other plant material used. If applicable, state the seed stock centre and catalogue number. If plant specimens were collected from the field, describe the collection location, date and sampling procedures.*
Novel plant genotypes	*Describe the methods by which all novel plant genotypes were produced. This includes those generated by transgenic approaches, gene editing, chemical/radiation-based mutagenesis and hybridization. For transgenic lines, describe the transformation method, the number of independent lines analyzed and the generation upon which experiments were performed. For gene-edited lines, describe the editor used, the endogenous sequence targeted for editing, the targeting guide RNA sequence (if applicable) and how the editor was applied.*
Authentication	*Describe any authentication procedures for each seed stock used or novel genotype generated. Describe any experiments used to assess the effect of a mutation and, where applicable, how potential secondary effects (e.g. second site T-DNA insertions, mosiacism, off-target gene editing) were examined.*
